# Accuracy of prognostic serological biomarkers in predicting liver fibrosis severity in people with metabolic dysfunction-associated steatotic liver disease: a meta-analysis of over 40,000 participants

**DOI:** 10.3389/fnut.2024.1284509

**Published:** 2024-02-14

**Authors:** Sergio M. López Tórrez, Camila O. Ayala, Paula Bayer Ruggiro, Caroline Abud Drumond Costa, Mario B. Wagner, Alexandre Vontobel Padoin, Rita Mattiello

**Affiliations:** ^1^School of Medicine, Graduate Program in Medicine and Health Sciences, Pontifícia Universidade Católica de Rio Grande do Sul (PUCRS), Porto Alegre, Brazil; ^2^School of Medicine, Postgraduate Program in Pediatrics and Child Health, Pontifícia Universidade Católica de Rio Grande do Sul (PUCRS), Porto Alegre, Brazil; ^3^School of Medicine, Pontifícia Universidade Católica de Rio Grande do Sul (PUCRS), Porto Alegre, Brazil; ^4^School Medicine, Universidade Federal de Rio Grande do Sul (UFRGS), Porto Alegre, Brazil; ^5^School of Medicine, Postgraduate Program in Epidemiology, Universidade Federal de Rio Grande do Sul (UFRGS), Porto Alegre, Brazil

**Keywords:** prognosis, liver biopsy, metabolic dysfunction-associated steatotic liver disease, non-invasive tests, meta-analysis

## Abstract

**Introduction:**

A prognostic model to predict liver severity in people with metabolic dysfunction-associated steatotic liver disease (MASLD) is very important, but the accuracy of the most commonly used tools is not yet well established.

**Objective:**

The meta-analysis aimed to assess the accuracy of different prognostic serological biomarkers in predicting liver fibrosis severity in people with MASLD.

**Methods:**

Adults ≥18 years of age with MASLD were included, with the following: liver biopsy and aspartate aminotransferase-to-platelet ratio (APRI), fibrosis index-4 (FIB-4), non-alcoholic fatty liver disease fibrosis score (NFS), body mass index, aspartate aminotransferase/alanine aminotransferase ratio, diabetes score (BARD score), FibroMeter, FibroTest, enhanced liver fibrosis (ELF), Forns score, and Hepascore. Meta-analyses were performed using a random effects model based on the DerSimonian and Laird methods. The study’s risk of bias was assessed using the Quality Assessment of Diagnostic Accuracy Studies-2.

**Results:**

In total, 138 articles were included, of which 86 studies with 46,514 participants met the criteria for the meta-analysis. The results for the summary area under the receiver operating characteristic (sAUROC) curve, according to the prognostic models, were as follows: APRI: advanced fibrosis (AF): 0.78, any fibrosis (AnF): 0.76, significant fibrosis (SF): 0.76, cirrhosis: 0.72; FIB-4: cirrhosis: 0.83, AF: 0.81, AnF: 0.77, SF: 0.75; NFS: SF: 0.81, AF: 0.81, AnF: 0.71, cirrhosis: 0.69; BARD score: SF: 0.77, AF: 0.73; FibroMeter: SF: 0.88, AF: 0.84; FibroTest: SF: 0.86, AF: 0.78; and ELF: AF: 0.87.

**Conclusion:**

The results of this meta-analysis suggest that, when comparing the scores of serological biomarkers with liver biopsies, the following models showed better diagnostic accuracy in predicting liver fibrosis severity in people with MASLD: FIB-4 for any fibrosis, FibroMeter for significant fibrosis, ELF for advanced fibrosis, and FIB-4 for cirrhosis.

Clinical trial registration: [https://clinicaltrials.gov/], identifier [CRD 42020180525].

## 1 Introduction

Metabolic dysfunction-associated steatotic liver disease (MASLD) is defined as the presence of hepatic steatosis along with at least one of five cardiometabolic risk factors that correspond to the components of metabolic syndrome (MetS) (1). The scenario of MASLD is evolving rapidly; according to the Global Burden of Disease study, MASLD increased considerably in both adolescents and adults between 1990 and 2019 (2, 3). In adolescents, the increase was from 3.73% in 1990 to 4.71% in 2019—an increase of 26.27% (2). In adults, the incidence of MASLD cases increased by 95.4% from 88,177 (95% uncertainty interval (95% UI): 62,304–128,319) in 1990 to 172,330 (95% UI: 125,775–243,640) in 2019. Deaths from MASLD increased by 80.2% from 93,758 (95% UI: 71,657–119,097) per 100,000 population in 1990 to 168,969 (95% UI: 130,575–211,295) per 100,000 population in 2019 (3).

Due to the burden of this disease, early diagnosis of MASLD is an important clinical strategy to prevent its rapid progression to the most severe stages of the disease. According to different international guidelines, liver biopsy is still considered the gold standard for diagnosing liver fibrosis in MASLD (4, 5). However, it is an invasive test that is not free of complications and is not recommended for monitoring disease severity (6). Therefore, the clinical practice guidelines for the management of MASLD recommend the use of non-invasive tests as a resource before the need for liver biopsy in order to stage the disease of fibrosis. These are non-invasive methods that make it feasible to assess disease progression (7).

Different studies have evaluated the diagnostic performance of prognostic models using biomarkers in MASLD (8–10). A meta-analysis of 64 studies published until 2017 compared the diagnostic performance of aspartate aminotransferase-to-platelet ratio index (APRI), fibrosis index-4 (FIB-4), fibrosis score for non-alcoholic fatty liver disease score (NFS), body mass index (BMI), aspartate aminotransferase (AST)/alanine aminotransferase (ALT) ratio (AST/ALT ratio), diabetes score (BARD score), FibroScan M probe, FibroScan XL probe, shear wave elastography (SWE), and magnetic resonance elastography (MRE) for staging significant fibrosis (SF), advanced fibrosis (AF), and cirrhosis in MASLD. This study concluded that MRE and SWE may provide better diagnostic accuracy for staging fibrosis in patients with MASLD, with the following results for the area under the receiver operating characteristic (AUROC) curve: SF: MRE: 0.88 and SEW: 0.89;: MRE: 0.93 and SEW: 0.91; and cirrhosis: MRE: 0.92 and SEW: 0.97 (8).

Similarly, a systematic review of 38 studies aimed to evaluate the common non-invasive tests, NFS, enhanced liver fibrosis (ELF), transient elastography, and MRE, in obese patients with SF, AF, and cirrhosis. Evidence showed better accuracy of complex biomarker panels: NFS: summary receiver operator characteristic (SROC): 0.79–0.81 vs. ELF: 0.96; however, the search focused only on studies published until 2016, in English, in four databases, and in individuals with obesity (9). Finally, a recent meta-analysis of 37 studies evaluated the individual diagnostic performance of liver stiffness measurement by vibration-controlled transient elastography (LSM-VCTE), FIB-4, and NFS to derive diagnostic strategies that could reduce the need for liver biopsies. The AUROC results of individual LSM-VCTE, FIB-4, and NFS for AF were 0.85, 0.76, and 0.73, respectively. However, only two invasive tests were included in just one stage of liver fibrosis (10).

Considering the growing body of evidence and lack of consensus on the diagnostic performance of clinical scores, this systematic review and meta-analysis aimed to assess the accuracy prognostic serological biomarkers (APRI, FIB-4, NFS, BARD score, FibroMeter, FibroTest, ELF, Forns score, and Hepascore) in predicting liver fibrosis severity in people with MASLD.

## 2 Materials and methods

This systematic review and meta-analysis followed the Preferred Reporting Items for Systematic Reviews and Meta-Analyses of Diagnostic Test Accuracy Studies (PRISMA-DTA) guidelines (Supplementary Table S1) (11). The protocol for this meta-analysis was registered in the International Prospective Register of Systematic Reviews database (PROSPERO) under the number CRD42020180525.

### 2.1 Literature search strategy

This systematic review aimed to answer the following research questions: What is the diagnostic accuracy of the most clinically used serological biomarkers in predicting liver fibrosis severity in people with MASLD? The strategy was based on the participants, index tests, and target condition (PIT) criteria: P: adults ≥18 years with MASLD; I: APRI, FIB-4, NFS, BARD score, FibroMeter, FibroTest, ELF, Forns score, and Hepascore; and T: liver fibrosis. Liver biopsy was used as the reference standard.

We searched the following databases from their inception through December 2021: The Cochrane Hepato-Biliary Group Diagnostic Test Accuracy Studies Register; Medical Literature Analysis and Retrieval System Online (MEDLINE) [via Public/Publisher MEDLINE (PUBMED)]; Excerpt Medical dataBASE (EMBASE); Scientific Electronic Library Online (SciELO); Latin American and Caribbean Health Sciences Literature (LILACS); Cumulative Index to Nursing and Allied Health Literature (CINAHL); and Web of Science (WOS). The reference lists from eligible studies were manually searched to identify additional potentially relevant studies. In addition, we manually searched the abstracts of books from the American Association for the Study of Liver Diseases (AASLD) meetings and European Association for the Study of the Liver (EASL) meetings from the last 10 years. The MEDLINE search strategy was created and adapted for the other databases. There was no language or year of publication restrictions (Supplementary Text S1).

### 2.2 Eligibility criteria

The eligibility criteria were the PIT criteria described above. Studies were included if they defined liver fibrosis according to the histological classification of the Clinical Research Network (12), included at least 20 adult patients, and provided sensitivity (Sen), specificity (Spe), sample size, or enough information to obtain true positives (TP), false positives (FP), true negatives (TN), and false negatives (FN).

Studies were excluded if participants had viral, autoimmune, or hepatic diseases and chronic hepatitis. Case series, experimental models, replies to letters, editorials, and duplicate publications were also excluded. Studies were considered duplicates if they belonged to the same study group and reported the same inclusion date and individual characteristics. In the case of duplicate studies, the one with the largest sample size was considered.

### 2.3 Selection of studies

Three review authors (SLT, PBR, and COA) independently selected the articles according to the eligibility criteria in two stages. The first selection stage consisted of screening the titles and abstracts of the articles identified through database searches. In the second stage, full-text articles were assessed using the same methodology. In the case of disagreement between the reviewers, a fourth reviewer (RM) assessed the articles according to the eligibility criteria to resolve any discrepancies.

### 2.4 Data extraction

Three authors (SLT, PBR, and COA) independently extracted the following data from the selected articles: first author; year of publication; type of paper; study design; study period; country; institution; number of participants; age (years); sex (percentage of males); race (percentages); BMI [kilograms (kg)/meters^2^ (m^2^)]; hypertension (percentage of participants); diabetes (percentage of participants); dyslipidemia (percentage of participants); MetS (percentage of participants); laboratory tests (AST, ALT, AST/ALT ratio, platelets, glycosylated hemoglobin (HbA1C), glycemia, triglycerides, and cholesterol); and score models (APRI, FIB-4, NFS, BARD score, FibroMeter, FibroTest, ELF, Forns score, and Hepascore). For diagnostic parameters, we considered cutoff values, AUROC, Sen, Spe, TP, FP, TN, and FN. When the authors did not describe TP, FP, TN, or FN, these were calculated based on the Sen and Spe and the number of participants in each study to obtain the values for each model.

### 2.5 Risk of bias assessment

Three authors (SLT, PBR, and COA) independently assessed the risk of bias in the primary studies using the Quality Assessment of Diagnostic Accuracy Studies-2 (QUADAS-2) (13). QUADAS-2 is a tool for evaluating the quality of primary diagnostic studies by examining quality separately in terms of “risk of bias” and “concerns regarding applicability.” Risk of bias assessment items were organized into four domains: patient selection, index test, reference standard, and flow and timing. The applicability of a study was evaluated for the first three key domains and rated as “yes,” “no,” or “unclear,” where “yes” indicated a low risk of bias, “no” indicated a high risk of bias, and “unclear” indicated a lack of sufficient information (13). Disagreements were resolved by consulting a fourth reviewer (RM) to establish a consensus. The methodological quality of individual studies was visualized using the *robvis* web app, which depicts the plots obtained from these analyses (14).

### 2.6 Data synthesis and analysis

For inclusion in the meta-analysis, the score model should have been used in at least three studies in predicting liver fibrosis severity in people with MASLD. Diagnostic performance statistics were obtained for each study, including Sen, Spe, diagnostic odds ratio (DOR), positive likelihood ratio (LR+), and negative likelihood ratio (LR-), with their respective 95% confidence interval (95% CI). Then, for the DOR, LR+, and LR-, summarized meta-analytical estimates were obtained using a random effects model based on obtaining the variance between studies using the DerSimonian and Laird methods. Heterogeneity was evaluated using Cochran’s Q (Q) statistic and I^2^ statistic. The Cochran’s Q statistic of homogeneity was measured based on the null hypothesis that all eligible studies have the same underlying effect size. The I^2^ statistic, which represents the variability between studies, was 0–40%, 40–70%, and 70–100%, indicating low, moderate, and high variance, respectively (15, 16). In addition, summary area under the receiver operating characteristic (sAUROC) curve was obtained using a mixed linear model with known variance estimates according to Reitsma’s method. The area under curve (AUC) values were interpreted as follows: <0.5 indicated low accuracy, 0.6 to 0.79 indicated moderate accuracy, 0.8–0.90 showed good accuracy, and > 0.90 represented excellent accuracy (17). A sensitivity analysis was performed to assess whether the results changed when only studies that included the most frequently found scores, FIB-4, APRI, and NFS, and without any fibrosis severity (AF, SF and cirrhosis) were used. All calculations were performed with R version 4.1.3 and Rstudio version 2022.02.1 (Build 461) using the Meta-Analysis of Diagnostic Accuracy (MADA) version 0.5.10 package.^1^

The TP, FP, FN, and TN numbers were extracted to construct the 2×2 tables, and the values for each reported test cutoff were calculated. In some studies that did not have the numbers, the prevalence, sensitivity, specificity, and sample size were calculated.^2^

The diagnostic accuracy of the index tests was evaluated in the following dichotomized groups: any fibrosis (AnF) (F0 vs. F1-4), SF (F0-1 vs. F2-4), AF (F0-2 vs. F3-4), cirrhosis (F0-3 vs. F4).

## 3 Results

### 3.1 Identification and selection of studies

**Figure 1:**
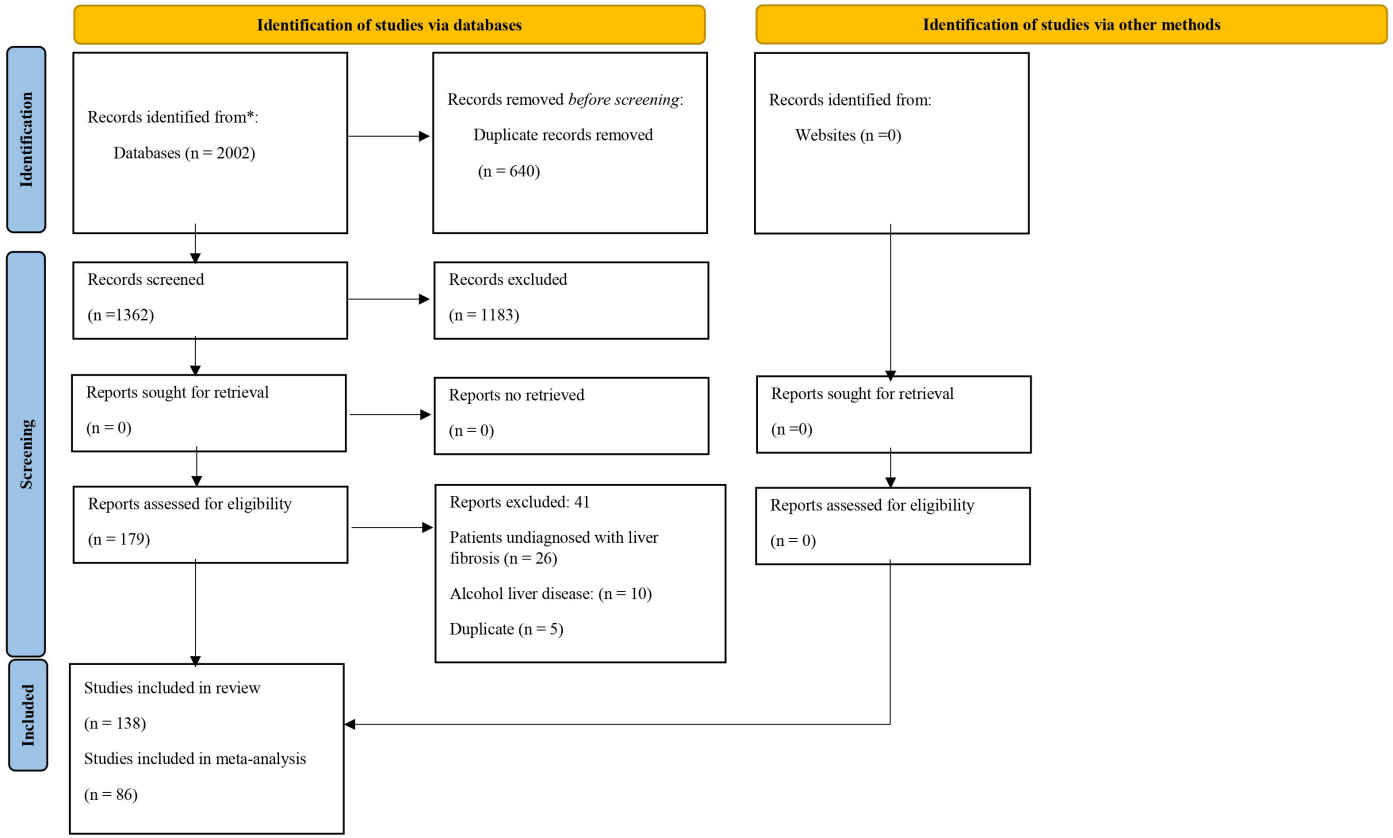
PRISMA 2020 flowchart of the study selection process.

The search strategy identified 2002 articles. Of these, 640 articles were duplicates, leaving 1,362 for title and abstract assessment. At this stage, 1,183 articles were excluded: 353 on other populations with chronic hepatitis; 130 on patients on autoimmune medication; 74 on animal studies; 198 on alcoholic liver disease; and 428 that did not involve the evaluation or validation of model performance. One hundred and seventy-nine studies were read in full, of which 41 studies were excluded: 26 studies did not include patients diagnosed with hepatic fibrosis; 10 on alcoholic liver disease; and 5 duplicates. Thus, 138 articles were included in this systematic review, of which 86 were included in the meta-analysis and met the eligibility criteria in Figure 1.

### 3.2 Characteristics of the included studies

The characteristics of the studies included in the systematic review are described in Table 1. The articles were published between 2004 (123) and 2021 (29, 33, 153). The majority were cross-sectional (68%) (20–22, 27–29, 31–35, 40–44, 46–48, 50–55, 58, 59, 62, 65, 69, 70, 75, 78, 79, 83, 84, 86, 88, 90–93, 95, 97–100, 103–105, 108, 109, 111–114, 116, 117, 119–122, 124, 126–128, 130–136, 138, 140–143, 146, 147, 149–151, 156). Regarding the type of publication, 70.3% of the studies were full-text articles (18, 20, 22, 25, 26, 28–31, 33, 35, 37, 39–42, 44–50, 52, 53, 55, 57, 58, 60, 62, 63, 65, 66, 68, 70, 74, 75, 77–79, 82–88, 90–100, 103, 106, 108, 109, 111–122, 124, 126, 128, 130–135, 138, 140–143, 146, 147, 149–154), and the remaining 29.7% were conference abstracts (19, 21, 23, 24, 32, 34, 36, 38, 43, 51, 54, 56, 60, 61, 64, 67, 69, 71–73, 76, 80, 81, 89, 101, 102, 104, 105, 107, 110, 118, 123, 125, 127, 129, 137, 139, 144, 145, 155). Regarding the geographical origin of the studies, most studies were conducted in Europe (41%) (20, 25, 27, 28, 31, 35–37, 40–44, 47, 51–55, 58, 61, 62, 66, 69, 72, 73, 84–86, 88, 94, 97–100, 102, 103, 105–107, 110, 114, 117, 120, 122, 123, 125, 126, 138–141, 144, 153) and Asia (30%) (22, 29, 46, 59, 67, 70, 71, 74–76, 78, 79, 83, 91, 93, 95, 109, 111, 112, 118, 121, 130–132, 142, 143, 146–152, 154). The total study population consisted of 46,514 participants. The sample size ranged from 29 (46) to 3022 (28) patients. The mean age of the participants ranged from 30 to 67 years old. In 48% of the studies, the majority of participants were male (19, 20, 25, 27, 29–31, 35, 37, 40–42, 44, 46–48, 52–57, 61, 62, 66, 75, 78, 80, 83–86, 91, 93, 95, 97, 98, 103, 106, 107, 112, 114, 120–122, 124, 126, 132, 138, 140–143, 149–151, 153, 154, 156). The mean BMI ranged from 25 (46, 146, 151) to 52.9 (101) kg/m^2^.

**Table 1:** Characteristics of studies included in the systematic review.

References	Country/Region	Type of publication	No. patients	Age (SD)	Male %	BMI (SD)	Stage system	Fibrosis 0/1	F1	F0-F1-F2%	F2	F2-F3-F4%	F3	F3-F4%	F4	Serological biomarkers
Abe et al. (18)	Japan	Article	289	54.8 ± 14	55	27.6 ± 4.7	Brunt	12.1	39.1	68.1	16.9	49.3	14.8	32.4	17.6	FIB-4, APRI, NFS
Adams et al. (19)	Australia	Abstract	119	48.7 ± 13	54	?	Kleiner and Brunt	41.0	?	?	?	?	?	?	?	APRI, Hepa score, FibroTest
Adams et al. (20)	Australia/Italy	Article	242	46.8 ± 12	60.3	30.2 ± 6	Kleiner and Brunt	35.9	23.9	78.0	18.1	40.1	12.3	22.0	9.5	FIB-4, APRI, Hepa score, FibroTest, BARD score
Ahmed et al. (21)	United States	Abstract	771	?	?	?	Batts Ludwig	?	?	?	?	?	?	?	?	FIB-4, APRI
Aida et al. (22)	Japan	Article	148	61 ± 12	36	26.9 ± 1.25	Kleiner and Brunt	18.9	34.4	71.5	18.2	46.4	16.8	28.2	11.4	FIB-4, APRI
Alkhouri et al. (23)	United States	Abstract	78	30 ± 9	32	?	?	35	42	80	13	23		10		FIB-4, APRI, NFS
Anam et al. (24)	?	Abstract	40	?	?	?	Kleiner and Brunt	40.9	27	80	12.1	32.1	10.7	20	9.3	FIB-4, APRI, NFS, FibroMeter, BARD score
Angelidi et al. (25)	Greece	Article	110	60.1 ± 9.5	52.7	?	?	?	?	?	?	?	?	?	?	FIB-4, APRI, NFS, BARD score
Angulo et al. (26)	United States/United Kingdom/Italy/ Australia	Article	1,014	46.9 ± 0.4	58	31.3 ± 0.2	Kleiner and Brunt	34.6	24.7	73.2	13.9	40.5	15.8	26.6	10.8	FIB-4, APRI, NFS, BARD score
Angulo et al. (27)	United States/United Kingdom/Italy/ Australia	Article	733	47.7 ± 13.2	52.2	32.3 ± 0.	Kleiner and Brunt	?	26.0	72.9	13.6	40.7	13.0	27.1	14.1	NFS
Anstee et al. (28)	United States/Europe	Article	3,202	57.5 ± 5.6	47	?	?	26	29	100	45	145	43	100	57	FIB-4, NFS, ELF
Amernia et al. (29)	Iran	Article	205	42.9 ± 10.9	70.2	?	?	?	45.9	78.6	32.7	54.1	14.1	21.4	7.3	FIB-4, APRI
Arora et al. (30)	United States	Article	141	56 ± 4.3	65	?	?	?	?	?	?	?	?	?	?	FIB-4, APRI, NFS, BARD score
Aykut et al. (31)	Turkey	Article	88	46 ± 9	56	30.3 ± 4.6	Kleiner and Brunt	26.0	24.0	69.0	19.0	50.0	21.0	31.0	10.0	NFS, FibroMeter
Balakrishnan et al. (32)	United States	Abstract	122	47 ± 9	20	34 ± 7.5	Kleiner and Brunt	?	?	?	?	?	?	?	?	FIB-4, APRI, NFS, BARD score
Balakrishnan et al.(33)	United States	Article	99	46.8 ± 11.5	26.3	32.4 ± 6.8	Brunt	46.3	38.3	90.7	44.4	63.6	8.1	19.2	11.1	BARD score, FIB-4, APRI, NFS
Barritt et al. (34)	United States	Abstract	859	57 ± 9	38	?	?	?	?	?	?	?	?	?	?	APRI, NFS
Boursier et al. (35)	France	Article	588	55.9 ± 12	57.3	31.7 ± 5.8	Kleiner and Brunt	9	25.9	61.5	26.5	63.3	24.8	38.6	13.8	FIB-4, APRI, NFS, FibroMeter, Hepa score, FibroTest, BARD score
Boursier et al. (36)	France	Abstract	618	?	?	?	?	?	?	?	?	?	?	?	?	NFS, FibroMeter
Boursier et al. (37)	France	Article	938	56.5 ± 12.1	58.5	31.8 ± 5.8	?	9.5	22.8	69.2	26.9	57.7	27.4	30.8	13.4	FIB-4, NFS, FibroTest, FibroMeter, Hepascore
Brandman et al. (38)	United States	Abstract	1,483	50 ± 10	36	?	?	?	?	?	?	?	?	10	?	FIB-4, APRI, NFS, BARD score
Bril et al. (39)	United States	Article	162	57 ± 9	82	34.7 ± 4.6	Kleiner and Brunt	25.1	41.7	83.5	16.5	33.1	12.5	16.5	3.9	FibroTest
Broussier et al. (40)	France	Article	283	56.5 ± 10	53.4	32.9 ± 6.6	?	?	?	?	?	?	?	54.8	?	FIB-4, FibroMeter
Cales et al. (41)	France	Article	235	51.1 ± 11	74.5	28.7 ± 4.9		?	28.9	81.2	8.9	27.7	8.1	18.7	10.6	APRI, NFS, FibroMeter
Cales et al. (42)	France	Article	226	50.9 ± 10.8	75.2	28.7 ± 4.9	Kleiner and Brunt	26.1	29.7	77.5	21.6	44.5	16.2	22.5	6.3	NFS, FibroMeter
Cebreiros et al. (43)	Spain	Abstract	55	43.9 ± 12	24.6	49.9	Metavir	?	?	?	?	?	?	?	?	FibroMeter, ELF
Cengiz et al. (44)	Turkey	Article	123	49 ± 11	56.1	29.5 ± 0.58	Kleiner and Brunt	64.2		86.2	22	35.8	8.9	13.8	4.9	FIB-4, APRI
Chan et al. (45)	Malaysia	Article	147	50.5 ± 11	54.4	29.3 ± 4.5	Kleiner and Brunt	29.3	41.5	79	8.2	29.2	19	21	2	NFS
Chowdhury et al. (46)	India	Article	29	43 ± 4.9	75.8	25.1 ± 2.6	Kleiner and Brunt	41.3	20.6	77.5	10.3	37.9	6.8	27.5	20.6	APRI
Cichoz-Lach et al. (47)	Poland	Article	126	42.7 ± 13	57.9	28.5 ± 2.6	Kleiner and Brunt	26.1	35.7	78.5	16.6	38.0	19.0	21.0	2.3	NFS, BARD score
Cui et al. (48)	United States	Article	102	51.3 ± 14	58.8	31.7 ± 5.5	Kleiner and Brunt	47.1	25.5	81.4	8.8	21.5	12.7	18.6	5.9	FIB-4, APRI, NFS, BARD score
de Carli et al. (49)	Brazil	Article	324	38.7 ± 10.7	34.5	43.8 ± 4.8	Kleiner and Brunt	?	40.8	91.1	4.3	13.2	8.6	8.9	0.3	FIB-4, APRI, NFS, BARD Score
de Cleva et al. (50)	Brazil	Article	131	45.8 ± 11	?	47.8 ± 6.3	Kleiner and Brunt	56.5	29	92.3	6.8	14.4	3.8	7.6	3.8	APRI
Demir et al. (51)	Germany	Abstract	323	?	?	?	?	?	?	?	?	?	?	?	?	NFS, BARD score
Demir et al. (52)	Germany	Article	165	44.8 ± 12	60	28.6 ± 4.3	Kleiner and Brunt	3.6	49.0	87.6	35.1	47.1	9.6	12.0	2.4	FIB-4, NFS, BARD score
Dincses et al. (53)	Turkey	Article	52	45 ± 9	57.6	30.8 ± 5.4	Kleiner and Brunt	?	?	81	?	38	?	19	?	NFS, FibroMeter
Drolz et al. (54)	Germany	Abstract	101	54 ± 10	54	29 ± 1.8	?	?	25.7	45.5	19.8	53.4	13.8	33.6	19.8	FIB-4, APRI, NFS, BARD Score
Dvorak et al. (55)	Czech Republic	Article	56	44.1 ± 15	70	30 ± 3.7	Matteoni	?		51.7	17.8	48.0	16	30.2	14.2	FIB-4, APRI, NFS, ELF, BARD score
Eddowes et al. (56)	?	Abstract	356	53 ± 12	57	34.4 ± 6.5	Kleiner and Brunt	?	?	?	?	?	?	?	?	FIB-4, NFS, FibroMeter
Fagan et al. (57)	Australia	Article	329	45.9 ± 11	64.1	?	Metavir	?	?	?	?	?	?	23.7	?	ELF
Francque et al. (58)	Belgium	Article	542	43.5 ± 12.7	28.6	38.2 ± 6.4	Kleiner and Brunt	64.2	16.3	?	12.1	?	7.0	?	0.2	FIB-4, APRI, NFS, Forns score, BARD score
Fujii et al. (59)	Japan	Article	50	55.8 ± 15.2	26	27.1 ± 3.8	Kleiner and Brunt	?	28.0	56.0	28.0	54.0	26.0	44.0	18.0	APRI
Fujii et al. (60)	Japan	Abstract	122	59 ± 15.3	39	?	Kleiner and Brunt	?	?	55.0	?	?	?	38.0	?	BARD score
Gallego-Duran et al. (61)	Spain	Abstract	49	49 ± 13	61	?	Kleiner and Brunt	?	?	?	?	79.0	?	?	?	NFS, FibroTest
Guha et al. (62)	United Kingdom	Article	192	48.7 ± 12.5	64	32.4 ± 5.7	Kleiner and Brunt	16.1	19.0	77.0	17.0	40.0	13.0	23.0	10.0	ELF
Guillaume et al. (63)	France	Article	417	56.1 ± 1,211	59.2	33.3 ± 6.6	Kleiner and Brunt	29	23.5	67.4	27.3	?	32.4	40.1	7.7	FibroMeter, ELF
Guturu et al. (64)	United States	Abstract	118	?	?	?	Batts Ludwig	?	39.8	75.3	19.4	43.9	8.4	24.5	16.1	APRI, BARD score
Harrison et al. (65)	United States	Article	827	49 ± 5.6	49	33	Kleiner and Brunt	?	24.0	?	80.8	?	?	?	?	BARD score
Hagström et al. (66)	Sweden	Article	646	50 ± 14.8	62	28 ± 3.7	Kleiner	65	40	88	23	35	9	11	3	NFS, BARD score, APRI, FIB-4
Huang et al. (67)	Singapore	Abstract	161	60 ± 14	?	26.8 ± 4.6	?	?	?	?	?	?	?	?	?	FIB-4, APRI, NFS, BARD score
Inadomi et al. (68)	Japan	Article	200	595 ± 17	48	28.1 ± 6.8	Kleiner and Brunt	?	37.5	76	22	58.5	32	36.5	4.5	FIB-4, ELF
Isgro et al. (69)	Italy	Abstract	74	44.3 ± 4.9	?	?	?	8.1	45.8	93.2	39.2	46	5.4	6.8	1.4	ELF
Itoh et al. (70)	Japan	Article	400	56 ± 20	48.7	27.3 ± 9.8	Kleiner and Brunt	16.7	45.7	76.1	13.7	37.5	15.7	23.7	8	ELF
Joo et al. (71)	Korea	Abstract	315	?	?	?	?	?	?	?	?	?	?	?	?	FIB-4, NFS, BARD score
Joo et al. (72)	United Kingdom	Abstract	116	54.3 ± 10.7	?	?	?	?	?	?	?	?	?	?	?	FIB-4
Jouness et al. (73)	Italian	Abstract	254	?	?	?	?	?	?	?	?	?	?	?	?	FIB-4, NFS
Kao et al. (74)	Taiwan	Article	73	35.2 ± 7.7	31.5	41.2 ± 5.6	?	?	?	?	?	22.8	?	11.4	?	FIB-4, APRI, NFS
Kawamur et al. (75)	Japan	Article	90	51.2 ± 5.9	55.5	26, 1	Kleiner and Brunt	?	47.7	61	13.3	52.1	33.3	38.8	5.5	FIB-4, APRI
Kim et al. (76)	Korea	Abstract	481	?	?	?	Metavir	?	?	?	?	?	?	?	?	FIB-4, APRI, NFS, BARD score
Kim et al. (77)	United States	Article	142	52.8 ± 12	26.8	36.3 ± 7.4	Kleiner and Brunt	?	?	?	?	?	?	?	?	FIB-4, APRI, NFS, BARD Score
Kobayashi et al. (78)	Japan	Article	140	56 ± 6.8	54.3	27.1 ± 4	Matteoni	7.1	44.3	74.3	22.9	48.6	21.4	25.7	4.3	FIB-4, APRI
Kolhe et al. (79)	India	Article	100	47 ± 12.3	49	?	Metavir	?	?	73	?	?	?	27	?	FIB-4, APRI
Kosick et al. (80)	Canada	Abstract	541	50.5 ± 13	56.5	32.3 ± 5.5	?	?	?	?	?	?	?	?	45.5	FIB-4, APRI, NFS, BARD score
Kruger et al. (81)	United States	Abstract	111	?	?	?	Kleiner and Brunt	50.0	?	?	?	?	?	?	?	APRI, NFS
Kruger et al. (82)	South Africa	Article	111	52 ± 10	?	?	Kleiner and Brunt	?	?	?	?	?	?	?	17.0	APRI, NFS
Kumar et al. (83)	India	Article	120	39.1 ± 12	75	26.1 ± 3.6	Kleiner and Brunt	26.6	28.3	77.4	22.5	44.8	14.1	22.3	8.3	FIB-4, APRI, NFS, BARD score
Labenz et al. (84)	Germany	Article	261	51 ± 18.5	52.5	30.9 ± 6.9	Kleiner and Brunt	15.5	43.6	84.3	40.9	?	?	?	15.7	FIB-4, APRI, NFS
Lambrecht et al. (85)	Germany	Article	2088	54.5 ± 11.5	64.5	28.6 ± 5.2	?	?	?	?	?	?	?	?	?	FIB-4, APRI
Lang et al. (86)	Germany	Article	96	57 ± 14.6	53	31 ± 6.9	Kleiner and Brunt	?	30.8	?	67.7	130.4	44.4	63.1	18.7	FIB-4, NFS
Lardi et al. (87)	Brazil	Article	73	?	636	?	?	?	?	?	?	?	?	?	?	FibroTest
Lassailly et al. (88)	France	Article	288	41.6 ± 12	33.6	48.6 ± 8	Metavir	59.0	34.0	97.5	4.5	6.9	0.7	2.4	1.7	FibroTest
Le et al. (89)	?	Abstract	254	50.3 ± 10.5	35.4	34.2 ± 6	Metavir	?	?	?	?	44	?	23	?	FIB-4, APRI, BARD Score
Lee et al. (90)	United States	Article	107	48.9 ± 23	38.3	35.9 ± 3.7	?	20.5	18.6	68.0	28.9	48.14	16.8	32.0	14.9	FIB-4, NFS, FibroMeter, BARD Score
Liu et al. (91)	China	Article	349	40.2 ± 12.5	76.5	26.8 ± 3.3	Kleiner and Brunt	?	?	?	?	?	?	?	?	FIB-4
Loaeza-del-Castill et al. (92)	Mexico	Article	30	43 ± 12	43	?	Metavir	26.0	33.0	71.5	40.0	51.5	10.0	10.0	0.0	APRI
Loong et al. (93)	China	Article	215	52 ± 4	55.3	26.8 ± 1.3	?	?	?	?	40.9	27	80	12.1	32.1	FibroMeter
Luger et al. (94)	Austria	Article	46	42 ± 13	20	43.8 ± 4.3	Kleiner and Brunt	?	?	?	?	30	?	13	?	FIB-4, NFS
Mahadeva et al. (95)	Malaysia	Article	131	49.9 ± 12	52.7	?	Kleiner and Brunt	40.8	?	?	35.1	?	35.1	?	6.1	APRI, NFS
Marella et al. (96)	United States	Article	907	46.7 ± 12	32.6	39.9 ± 6 9	Kleiner and Brunt	32.9	36.4	87.2	17.9	30.7	6.9	12.8	5.9	FIB-4, APRI, NFS
McPherson et al. (97)	United Kingdom	Article	145	51 ± 12	61	35 ± 5	Kleiner and Brunt	25.0	43.0	78.0	13.0	29.0	10.0	19.0	9.0	FIB-4, APRI, NFS, BARD score
McPherson et al. (98)	United Kingdom/ Belgium/France	Article	634	49.8	54.8	34 ± 4.5	Kleiner and Brunt	37.4	23.2	?	14.2	?	17	?	8.2	FIB-4, APRI, NFS
McPherso et al. (99)	United Kingdom	Article	305	51 ± 12	60	33.6 ± 4.7	Kleiner and Brunt	?	?	80.5	?	37.5	?	20.5	?	FIB-4, NFS
Meneses et al. (100)	Spain	Article	50	49 ± 8	30	44.3 ± 5	Kleiner and Brunt	60	22	94	12	18	6	6	0	FIB-4, APRI, NFS, Forns score, BARD score
Miao et al. (101)	United States	Abstract	686	?	?	52.9 ± 9.7	?	?	?	?	?	12.3	?	3.1	?	FIB-4, NFS, BARD score
Miele et al. (102)	Italy	Abstract	82	46 ± 12	?	?	?	7.3	39	81.7	35.4	53.7	?	18.3	?	ELF
Miele et al. (103)	Italy	Article	82	46 ± 9	62	28 ± 22–38	?	7.3	39	82.7	35.4	53.7	6.1	18.3	12.2	ELF
Miller et al. (104)	United States	Abstract	354	50 ± 13	42.7	33.9 ± 8.5	?	?	?	?	73.7	?	?		26.3	FIB-4, APRI, NFS
Miller et al. (105)	United Kingdom	Abstract	42	?	?	?	?	?	?	?	?	?	?	?	?	FIB-4, NFS, BARD score
Munteanu et al. (106)	France/Italy/Brazil/ United Kingdom/Austria/Greece/ Spain	Article	600	53.2 ± 24	63.3	29.7 ± 0.25	Kleiner and Brunt	20.3	30.8	?	23.3	?	20.2	?	5.5	FIB-4, NFS, FibroTest, BARD score
Nascimben et al. (107)	France	Abstract	884	55 ± 12	61	30 ± 5	Kleiner and Brunt	?	?	?	?	?	?	?	?	FIB-4, APRI, NFS, BARD score
Nassif et al. (108)	Brazil	Article	298	40.1 ± 8	11.1	43.6 ± 10	?	?	?	?	?	?	7.3	?	?	BARD score
Okajima et al. (109)	Japan	Article	163	55.8 ± 14	49.5	27.2 ± 4.3	?	38	34.4	86.5	14.1	26.5	8	12.5	5.5	FIB-4, APRI
Pastor-Ramire et al. (110)	Spain	Abstract	1,256	54.1 ± 14	46	?	?	?	?	?	57.7	?	?	?	?	FIB-4, APRI, NFS, BARD score
Pathik et al. (111)	India	Article	110	42.3 ± 3.2	?	29.1	?	?	?	?	?	?	?	34.5	?	APRI, NFS
Peleg et al. (112)	Israel	Article	153	51.8 ± 17	55.5	29.9 ± 1.6	Metavir	?	?	79.1	?	?	?	20.9	?	FIB-4, APRI
Pérez-Gutiérrez et al. (113)	Mexico/Chile	Article	228	48.6 ± 12	49	?	Kleiner and Brunt	81.6	25.0	88.2	6.6	18.4	7.0	11.8	4.8	FIB-4, APRI, NFS, BARD score
Petta et al. (114)	Italy	Article	321	44.6 ± 12	67.5	29.3	Kleiner and Brunt	?	?	?	?	?	?	22.9	?	FIB-4, NFS
Petta et al. (115)	Italy. Hong Kong. France	Article	741	50.9 ± 12.7	60.2	29.6 ± 4.9	Kleiner and Brunt	?	?	?	?	34.3	?	30.9	?	FIB-4, NFS
Pimentel et al. (116)	Brazil	Article	158	36 ± 10	22.7	41 ± 5	?	?	7.5	30.3	85.9	48.1	61.9	12.0	13.8	NFS
Polyzos et al. (117)	Greece	Article	31	53.3 ± 2.7	25.8	32.2 ± 1.4	Kleiner and Brunt	?	?	?	?	?	?	22.5		APRI, NFS, ELF, FIB-4
Prasad et al. (118)	India	Abstract	240	39.3 ± 10	?	?	?	?	?	?	?	?	?	4	?	FIB-4, APRI, NFS
Qureshi et al. (119)	United States	Article	401	40.5 ± 8.5	17	48.4 ± 7.2	Kleiner and Brunt	43.4	40.0	35.9	86.5	13.8	27.3	11.4	13.5	NFS
Raszeja-Wyszomirska et al. (120)	Poland	Article	104	48 ± 12	65.4	29.6 ± 3	Kleiner and Brunt	?	?	84.6	?	?	?	14.4		BARD score
Rath et al. (121)	India	Article	60	39.7 ± 9.6	85	26.4 ± 3.3	Kleiner and Brunt	31.6	28.3	96.7	36.6	66	3.3	3.3	0	APRI, NFS, BARD score
Ratziu et al. (122)	France	Article	267	50.75 ± 9.4	58	> 27	Kleiner and Brunt	58.2	36.0	79.0	19.0	28.0	5.0	5.0	0	FibroTest
Ratziu et al. (123)	France	Abstract	89	?	?	?	Kleiner and Brunt	36.0	?	?	?	45, 0	?	11, 0	?	FibroTest
Ruffillo et al. (124)	Argentina	Article	138	49 ± 5.6	67	30, 3	Kleiner and Brunt	5.0	6.5	76.9	61.5	88.4	23.1	26.8	3.6	NFS, BARD score
Saez et al. (125)	Spain	Abstract	78	54.2 ± 11	39.7	?	?	?	?	?	?	55, 1	?	?	?	APRI, NFS, BARD score
Sebastiani et al. (126)	France/Italy	Article	190	51.2 ± 13	74.7	28.9 ± 5	Kleiner and Brunt	49.0	36.3	74.7	26.3	51.6	11.6	25.3	13.7	APRI, FibroTest
Seth et al. (127)	United States	Abstract	137	47 ± 11	22	32 ± 6.7	?	?	?	?	?	?	?	40	?	FIB-4, APRI, NFS, BARD score
Shah et al. (128)	United States	Article	541	47.5 ± 12	40	34.7 ± 6.5	Kleiner and Brunt	?	?	76.8	?	?	?	23.1	?	FIB-4
Shaheen et al. (129)	Canada	Abstract	44	51.5 ± 6.6	?	?	?	?	?	?	?	?	?	32	?	FIB-4, APRI, NFS
Shima et al. (130)	Japan	Article	278	57.8 ± 14.8	48.2	27.5 ± 4.7	Kleiner and Brunt	34.1	23.3	72.1	14.7	42.4	23	27.6	4.6	FIB-4, APRI
Shoji et al. (131)	Japan	Article	197	60 ± 14	45.1	27.5 ± 6.2	Kleiner and Brunt	40.6	?	63.9	23.3	59.3	20.8	36	15.2	FIB-4, APRI, NFS, BARD score
Shukla et al. (132)	India	Article	51	50.4 ± 11	53	?	Kleiner and Brunt	?	?	78.4	?	?	?	21.6	?	FIB-4
Siddiqui et al. (133)	United States	Article	145	52.9 ± 11	37.7	35.8 ± 19	Kleiner and Brunt	29	29	64.9	?	?	?	35.2	7.6	FIB-4, APRI, NFS, FibroMeter, BARD score
Siddiqui et al. (134)	United States	Article	1904	50.3 ± 12.2	47	34.4 ± 6.4	Kleiner and Brunt	24	28	72	20	48	20	28	8	FIB-4, APRI, NFS
Simo et al. (135)	United States	Article	225	43.2 ± 9.6	14.7	44.6 ± 5.4	Kleiner and Brunt	?	58.2	21.8	93.4	13.3	19.9	6.2	6.6	NFS
Singh et al. (136)	United States	Article	1,157	51.1 ± 11.5	35.4	35.5 ± 8.1	Kleiner and Brunt	?	?	68.2	?	?	?	38.1	?	FIB-4, APRI, NFS
Singh et al. (137)	?	Abstract	1969	?	?	?	?	?	?	?	?	?	?	7	?	FIB-4, APRI, NFS, BARD score
Sjowall et al. (138)	Sweden	Article	82	59.8 ± 11	67	28.9 ± 4.4	Kleiner and Brunt	?	?	?	?	?	?	17	?	APRI, NFS, BARD score
Stauber et al. (139)	Austria	Abstract	122	?	?	?	?	?	?	?	?	?	?	28	?	ELF
Staufer et al. (140)	Austria	Article	186	52 ± 5.2	57	30.5 ± 2.7	Kleiner and Brunt	?	?	61.8		55		27	?	FIB-4, FibroMeter, ELF
Subasi et al. (141)	Turkey	Article	142	45 ± 9	52.8	30.9 ± 5	Kleiner and Brunt	28.2	35.2	78.9	15.5	36.6	14.1	21.1	7	FIB-4, APRI, NFS, FibroMeter, BARD Score
Sumida et al. (142)	Japan	Article	576	52.3 ± 15	51	27.9 ± 4.9	Kleiner and Brunt	45.6	29.3	?	13.8	24.9	7.8	11.1	3.2	FIB-4, APRI, NFS, BARD score
Takeuchi et al. (143)	Japan	Article	71	50.8 ± 15.7	64.8	29.1 ± 5.1	Kleiner and Brunt	8	17	39	14	46	27	32	5	FIB-4
Tanwar et al. (144)	United Kingdom	Abstract	177	?	?	?	Kleiner and Brunt	59.0	19.2	75.7	17.5	23.8	13.6	23.8	10.2	FIB-4, APRI, NFS, ELF, BARD score
Thanapirom et al. (145)	?	Abstract	92	49.6 ± 13.7	44.9	27.4 ± 5.1	?	97.8	?	100	2, 2	?	?	?	?	FIB-4, APRI
Tomeno et al. (146)	Japan	Article	106	67 ± 7.8	41.5	25.8 ± 3.1	?	?	52.8	10.3	21.6	36.6	11.3	15	3.7	FIB-4
Treeprasertsuk et al. (147)	Thailand	Article	139	40.9 ± 13	47	36.1 ± 14.7	?	?	?	93.5	?	?	?	6.4	?	FIB-4, NFS, BARD score
Uy et al. (148)	Philippines	Abstract	61	46 ± 11	46	29.1 ± 4.3	?	?	?	?	?	?	?	9, 8	?	FIB-4, APRI, BARD Score
Wong et al. (149)	China	Article	246	51 ± 11	54.9	28 ± 4.5	Kleiner and Brunt	28.4	30.4	77.3	18.2	40.9	12.6	22.7	10.1	FIB-4, APRI, NFS, BARD score
Xun et al. (150)	China	Article	152	37.1 ± 9.7	79.6	26.1 ± 3.3	Kleiner and Brunt	31.6	33.5	84.0	19.1	34.9	13.8	15.8	1.9	FIB-4, APRI, NFS, BARD score
Yang et al. (151)	China	Article	453	36.5 ± 16.7	58.9	25.9 ± 3.6	Kleiner and Brunt	?	?	72, 2	?	?	?	27.8	?	FIB-4, APRI, NFS, FibroMeter, Forns score, BARD score
Yoneda et al. (152)	Japan	Article	235	59.9 ± 12	?	26.9 ± 4	Kleiner and Brunt	38.7	27.6	83.8	17.4	33.6	8.9	16.2	7.2	FIB-4, NFS, BARD Score
Younes et al. (153)	Italy, United Kingdom, and Spain	Article	1,173	40 ± 14.1	64.7	29.4 ± 7.5	Kleiner and Brunt									APRI, NFS, FIB-4, BARD score, Hepascore
Zhou et al. (154)	China	Article	207	41.8	73.4	?	?	?	47.8	38.2	96.1	10.1	14	3.9	3.9	FIB-4, APRI, NFS, BARD score
Zou et al. (155)	China	Abstract	107	?	?	?	?	?	?	?	?	?	?	?	28	FIB-4, APRI, NFS, BARD score

APRI, aspartate aminotransferase-to-platelet ratio index; ELF, enhanced liver fibrosis; FIB-4, fibrosis index-4; LR+, positive likelihood ratio; LR-, negative likelihood ratio; NFS, non-alcoholic fatty liver disease score; SD, standard deviation;?, not responded.

### 3.3 Serological biomarkers

The 138 included studies evaluated the nine serological biomarkers (FIB-4; FibroMeter; ELF; NFS; BARD; Hepascore; APRI; FibroTest; Forns score) for liver fibrosis. The most described was the FIB-4, in 89 studies (20–26, 28, 29, 32, 33, 35, 37, 38, 40, 44, 48, 49, 52, 54–56, 58, 66, 67, 71–75, 77–80, 83–86, 89–91, 94, 97–101, 104–107, 109, 110, 112–115, 118, 127–134, 136, 137, 140, 141, 143–146, 148–154), followed by the NFS score in 87 studies (22–27, 30–38, 41, 42, 47–49, 51–56, 58, 61, 66, 67, 71, 73, 74, 76, 77, 80–84, 86, 90, 95, 97–101, 104–107, 110, 111, 113–119, 121, 124, 125, 127, 129, 131, 133–138, 141, 144, 147, 149–154) and the APRI in 80 studies (19–21, 23–26, 29, 30, 32–35, 37, 38, 41, 42, 44, 46, 48, 49, 54, 55, 58, 59, 64, 66, 67, 74, 75, 77, 79–85, 89, 92, 95, 97, 100, 104, 105, 107, 110–113, 118, 121, 125–127, 129–131, 133, 134, 136–138, 141, 144, 145, 148–151, 153, 154). The least used were the ELF in 14 studies (46, 58, 60, 65, 66, 71–73, 105, 106, 120, 142, 143, 147), the Forns score in three studies (58, 100, 151), and the Hepascore in four studies (19, 20, 35, 153). The stage system used to perform the biopsy in most studies was the Kleiner and Brunt system in 55% of the studies (19, 20, 22, 24, 26, 27, 31, 32, 35, 39, 42, 44–50, 52, 53, 56, 58–63, 65, 68, 70, 75, 76, 81–84, 86, 91, 94–100, 106, 107, 113, 114, 117, 119–124, 126, 128, 130–136, 138, 140–144, 149–153). Regarding the severity of fibrosis, AF was the most diagnosed, with 182 studies (20–26, 28, 29, 32, 33, 35, 37, 38, 40, 44, 48, 49, 52, 54–56, 58, 66, 67, 71–75, 77–80, 83–86, 89–91, 94, 97–101, 104–107, 109, 110, 112–115, 118, 127–134, 136, 137, 140, 141, 143–154), followed by SF, with 140 studies (22–25, 30, 35, 36, 38, 41, 42, 47–49, 51, 52, 54, 55, 58, 61, 71, 73, 74, 76, 77, 81–84, 86, 90, 95, 98–101, 105–107, 110, 113, 115, 116, 119, 121, 124, 127, 129, 131, 134–136, 138, 144, 147, 150–152), then by any type of liver fibrosis (107, 112, 114, 120–122, 124, 126, 132, 138, 140–143, 149–151, 153, 154) and cirrhosis (8, 18, 19, 22, 26, 40, 47, 52, 92, 105, 111, 113, 116, 119, 128, 131, 154) in 18 and 16 studies, respectively (Supplementary Text S2 and Supplementary Tables S2, S3). The serological biomarker cutoff values for each severity level have been described in more detail in (Supplementary Table S4).

Table 1. Characteristics of the studies included in the systematic review.

### 3.4 Analysis of the quality and risk of bias in the included studies

**Figure 2:**
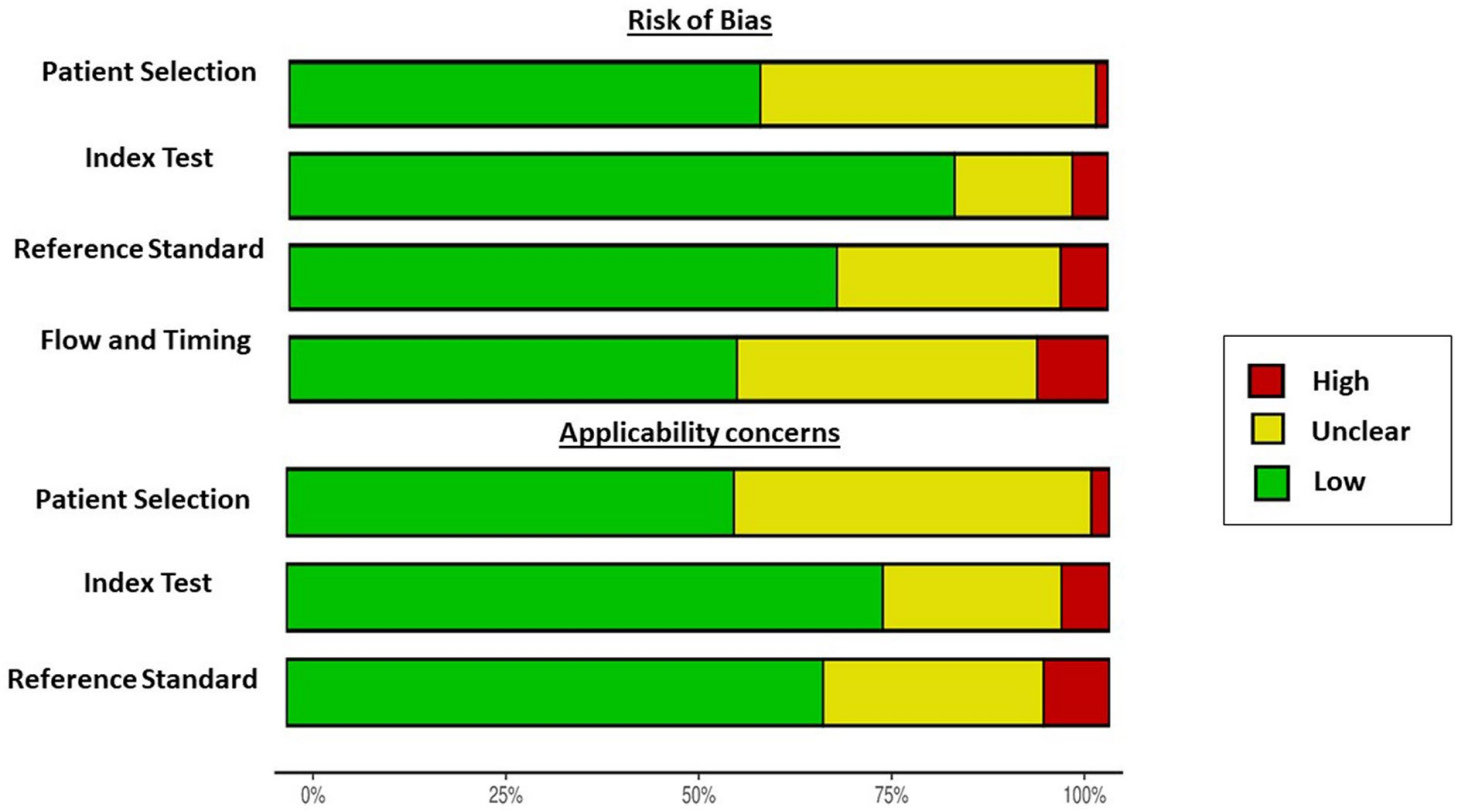
Graphical summary of the risk of bias of the included studies using the QUADAS-2 tool.

The quality assessment was performed using the QUADAS-2 tool as shown in Figure 2. Studies with patients with MASLD and other morbid conditions were considered a high applicability concern due to the consecutive or random sample of patients enrolled, a case–control design, and inappropriate inclusions such as populations with diabetes, obesity, high levels of transaminases, and selected age.

The risk of bias was unclear in 41% of the studies regarding patient selection (18, 19, 21, 22, 24, 31, 36–38, 51, 56, 59, 61, 72, 73, 76, 83, 85, 88–90, 94, 105, 107, 111, 119, 120, 125, 139, 142, 148, 152, 155, 157). Concerning the reference standard of the studies, several studies did not describe whether all patients received the reference standard and whether all patients were included in the studies, and therefore, 27% of the studies were unclear about the risk of bias (20, 25, 30, 34, 35, 46, 49, 57, 59, 66, 74, 79, 80, 86, 91, 93, 98, 100, 106–109, 115, 116, 118, 123, 129, 130, 138, 142–145, 149, 150, 154, 158). Most of the studies described the pre-specified thresholds (Supplementary Tables S5, S6).

### 3.5 Meta-analysis results

For inclusion in the meta-analysis, the score model should have been used in at least three studies in predicting liver fibrosis severity in people with MASLD. Only seven scores (APRI, FIB-4, NFS, BARD score, FibroMeter, FibroTest, and ELF) were used in at least three studies to evaluate the four degrees of liver fibrosis severity (AnF, SF, AF, and cirrhosis) and were therefore meta-analyzed (Supplementary Figure S1).

### 3.6 APRI

The APRI serological biomarker was evaluated for diagnostic accuracy in detecting AnF (> F1) (3 studies), SF (≥ F2–F4) (14 studies), AF (≥ F3) (33 studies), and cirrhosis (F4) (3 studies) (Supplementary Table S7).

#### 3.6.1 Diagnosis of AnF (F0 vs. F1–F4)

**Figure 3:**
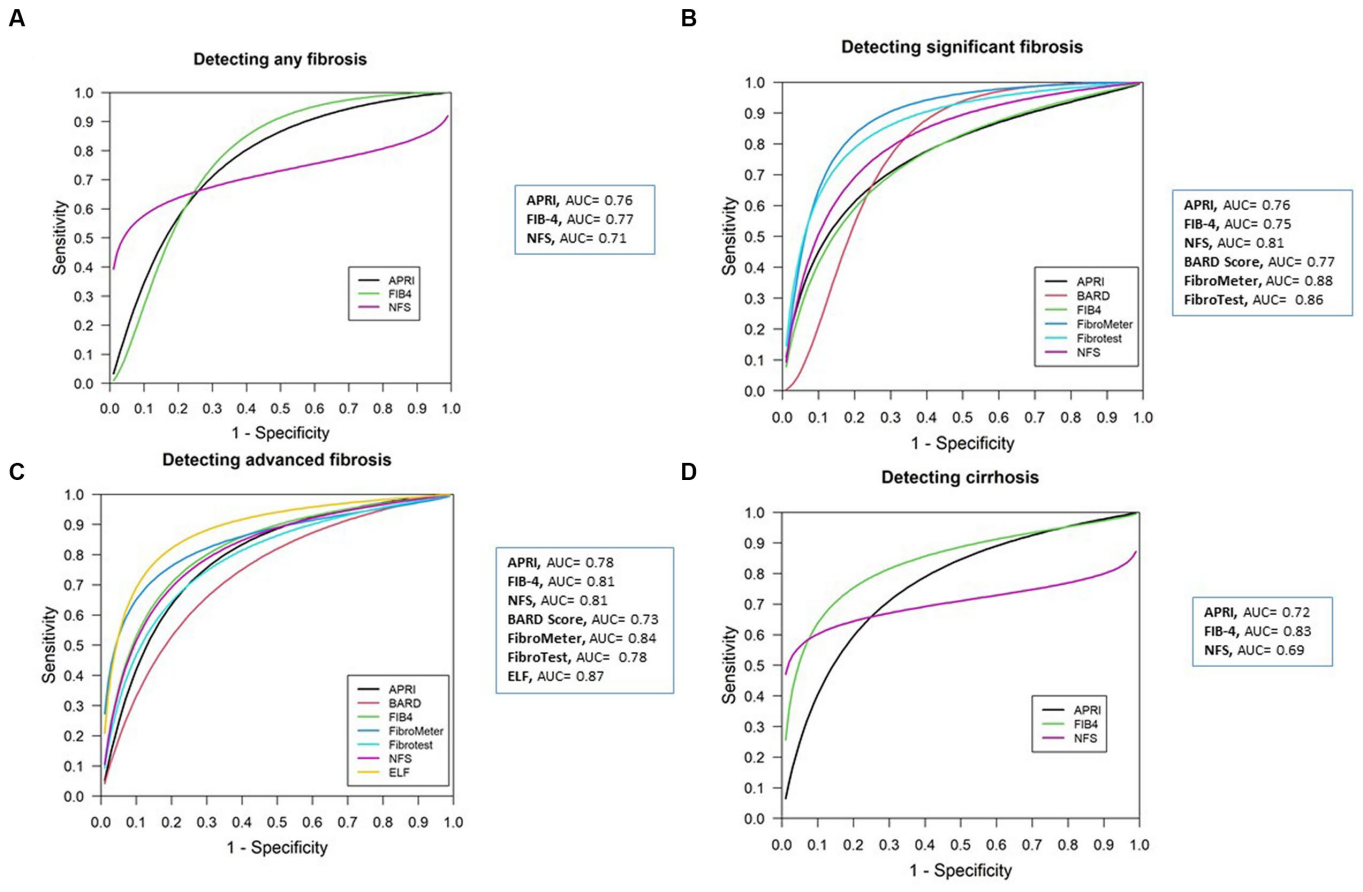
Summary AUROC plot of tests. **(A)** APRI, FIB-4, and NFS in detecting any fibrosis. **(B)** APRI, FIB-4, NFS, BARD score, FibroMeter, and FibroTest in detecting significant fibrosis. **(C)** APRI, FIB-4, NFS, BARD score, FibroMeter, FibroTest, and ELF in detecting advanced fibrosis. **(D)** APRI, FIB-4, and NFS in detecting cirrhosis.

The DOR of the APRI in the diagnosis of AnF was 5.61 (95% CI 4.61–6.82), the LR+ was 2.18 (95% CI 1.63–2.91), the LR- was 0.35 (95% CI 0.22–0.56), and moderate heterogeneity was detected (Q = 1.04, *p* = 0.59, I^2^ = 64.35%) (Table 2; Supplementary Figures S2, S3). The sAUROC had a moderate diagnostic accuracy of 0.76, Sen of 77% (95% CI 61–88%), and Spe of 64% (95% CI 48–78%) (Figure 3A, Supplementary Table S7, and Supplementary Figure S1).

**Table 2:** Comparison of serological biomarkers in predicting liver fibrosis severity in people with MASLD: DOR; LR+, and LR−.

	DOR	(95% CI)	Cochran’s Q	*p*	*I* ^2^	LR+	(95% CI)	LR-	(95% CI)
APRI									
Any fibrosis	5.61	(4.61–6.82)	1.04	0.59	64.35	2.18	(1.63–2.91)	0.35	(0.22–0.56)
Significant fibrosis	6.29	(4.47–8.92)	16.13	0.24	19.4	2.69	(2.23–3.23)	0.48	(0.40–0.58)
Advanced fibrosis	6.45	(4.83–8.60)	42.78	0.009	25.21	2.96	(2.49–3.52)	0.50	(0.43–0.57)
Cirrhosis	6.21	(4.34–8.89)	1.71	0.42	0	3.11	(2.15–4.50)	0.53	(0.31–0.89)
FIB-4									
Any fibrosis	6.57	(4.56–9.48)	5.35	0.25	25.24	2.32	(1.94–2.77)	0.38	(0.29–0.49)
Significant fibrosis	5.75	(4.11–8.05)	18.26	0.19	23.33	2.51	(2.07–3.05)	0.50	(0.43–0.59)
Advanced fibrosis	10.43	(7.25–15.02)	33.1	0.83	0	4.09	(3.33–5.02)	0.45	(0.39–0.52)
Cirrhosis	14.95	(9.96–22.44)	4.16	0.24	27.88	4.66	(2.41–9.02)	0.38	(0.19–0.78)
NFS									
Any fibrosis	4.85	(3.32–7.09)	6.63	0.15	39.66	2.27	(1.86–2.78)	0.49	(0.42–0.57)
Significant fibrosis	9.45	(5.17–17.5)	13.53	0.40	3.91	3.35	(2.42–4.63)	0.42	(0.33–0.54)
Advanced fibrosis	9.74	(6.69–14.17)	37.99	0.64	0	3.56	(2.93–4.32)	0.44	(0.38–0.51)
Cirrhosis	9.13	(4.25–19.62)	1.72	0.42	0	3.88	(2.35–6.39)	0.43	(0.32–0.58)
BARD score									
Significant fibrosis	5.98	(2.62–13.66)	4.11	0.53	0	2.49	(1.72–3.61)	0.46	(0.30–0.70)
Advanced fibrosis	4.34	(3.40–5.55)	26.11	0.16	23.4	1.88	(1.65–2.14)	0.48	(0.41–0.56)
FibroMeter									
Significant fibrosis	17.82	(4.91–64.7)	2.69	0.44	0	6.00	(2.72–13.23)	0.35	(0.18–0.67)
Advanced fibrosis	13.72	(7.51–25.07)	9.42	0.58	0	4.16	(2.89–5.99)	0.31	(0.24–0.40)
FibroTest									
Significant fibrosis	5.19	(1.77–15.18)	12.21	0.007	75.42	2.10	(1.36–3.25)	0.56	(0.36–0.85)
Advanced fibrosis	7.45	(5.15–10.77)	4.48	0.48	0	3.81	(2.18–6.64)	0.58	(0.43–0.79)
ELF									
Advanced fibrosis	18.82	(9.52–37.18)	7.05	0.21	29.08	4.42	(3.12–6.25)	0.29	(0.23–0.38)

APRI, aspartate aminotransferase-to-platelet ratio index; CI, confidence interval; DOR, diagnostic odds ratio; ELF, enhanced liver fibrosis; FIB-4, fibrosis index-4; I^2^, heterogeneity; LR+, positive likelihood ratio; LR-, negative likelihood ratio; NFS, non-alcoholic fatty liver disease fibrosis score; p, statistically significant value, MASLD, metabolic dysfunction-associated steatotic liver disease.

#### 3.6.2 Diagnosis of SF (F0–F1 vs. F2–F4)

The DOR of the APRI in the diagnosis of SF was 6.29 (95% CI 4.47–8.92), the LR+ was 2.69 (95% CI 2.23–3.23), the LR- was 0.48 (95% CI 0.40–0.58), and low heterogeneity was detected (Q = 16.13, *p* = 0.24, I^2^ = 19.40%) (Table 2; Supplementary Figures S4, S5). The sAUROC had a moderate diagnostic accuracy of 0.76, Sen of 63% (95% CI 53–72%), and Spe of 79% (95% CI 69–86%) (Figure 3B, Supplementary Table S7, and Supplementary Figure S1).

#### 3.6.3 Diagnosis of AF (F0–F2 vs. F3–F4)

The DOR of the APRI in the diagnosis of AF was 6.45 (95% CI 4.83–8.60), the LR+ was 2.96 (95% CI 2.49–3.52), the LR- was 0.50 (95% CI 0.43–0.57), and low heterogeneity was detected (Q = 42.78, *p* = 0.009, I^2^ = 19.40%) (Table 2; Supplementary Figures S6, S7). The sAUROC had a moderate diagnostic accuracy of 0.78, Sen of 60% (95% CI 50–69%), and Spe of 82% (95% CI 76–87%) (Figure 3C, Supplementary Table S7, and Supplementary Figure S1).

#### 3.6.4 Diagnosis of cirrhosis (F0–F3 vs. F4)

The DOR of the APRI in the diagnosis of cirrhosis was 6.21 (95% CI 4.34–8.89), the LR+ was 3.11 (95% CI 2.15–4.50), the LR- was 0.53 (95% CI 0.43–0.57), and no heterogeneity was detected (Q = 1.71, *p* = 0.42, I^2^ = 0%) (Table 2; Supplementary Figures S8, S9). The sAUROC had a moderate diagnostic accuracy of 0.72, Sen of 47% (95% CI 3–84%), and Spe of 87% (95% CI 50–98%) (Figure 3D, Supplementary Table S7, and Supplementary Figure S1).

### 3.7 FIB-4

The FIB-4 serological biomarker was evaluated for diagnostic accuracy in detecting AnF (> F1) (5 studies), SF (≥ F2–F4) (15 studies), AF (≥ F3) (43 studies), and cirrhosis (F4) (4 studies) (Supplementary Table S7).

#### 3.7.1 Diagnosis of AnF (F0 vs. F1–F4)

The DOR of the FIB-4 in the diagnosis of AnF was 6.57 (95% CI 4.56–9.48), the LR+ was 2.32 (95% CI 1.94–2.77), the LR- was 0.38 (95% CI 0.29–0.49), and low heterogeneity was detected (Q = 5.35, *p* = 0.25, I^2^ = 25.24%) (Table 2; Supplementary Figures S10, S11). The sAUROC had a moderate diagnostic accuracy of 0.77, Sen of 77% (95% CI 61–87%), and Spe of 68% (95% CI 57–78%) (Figure 3A, Supplementary Table S7, and Supplementary Figure S1).

#### 3.7.2 Diagnosis of SF (F0–F1 vs. F2–F4)

The DOR of the FIB-4 in the diagnosis of SF was 5.75 (95% CI 4.11–8.05), the LR+ was 2.51 (95% CI 2.07–3.05), the LR- was 0.50 (95% CI 0.43–0.59), and low heterogeneity was detected (Q = 18.26, *p* = 0.19, I^2^ = 23.33%) (Table 2; Supplementary Figures S12, S13). The sAUROC had a moderate diagnostic accuracy of 0.75, Sen of 64% (95% CI 52–74%), and Spe of 76% (95% CI 66–84%) (Figure 3B, Supplementary Table S7, and Supplementary Figure S1).

#### 3.7.3 Diagnosis of AF (F0–F2 vs. F3–F4)

The DOR of the FIB-4 in the diagnosis of AF was 10.43 (95% CI 7.25–15.02), the LR+ was 4.09 (95% CI 3.33–5.02), the LR- was 0.45 (95% CI 0.39–0.52), and no heterogeneity was detected (Q = 33.1, *p* = 0.83, I^2^ = 0%) (Table 2; Supplementary Figures 14, S15). The sAUROC had a good diagnostic accuracy of 0.81, Sen of 60% (95% CI 52–68%), and Spe of 87% (95% CI 82–91%) (Figure 3C, Supplementary Table S7, and Supplementary Figure S1).

#### 3.7.4 Diagnosis of cirrhosis (F0–F3 vs. F4)

The DOR of the FIB-4 in the diagnosis of cirrhosis was 14.95 (95% CI 9.96–22.44), the LR+ was 4.66 (95% CI 2.41–9.02), the LR- was 0.38 (95% CI 0.19–0.78), and low heterogeneity was detected (Q = 4.16, *p* = 0.24, *I*^2^ = 27.88%) (Table 2; Supplementary Figures S16, S17). The sAUROC had a good diagnostic accuracy of 0.83, Sen of 69% (95% CI 43–86%), and Spe of 87% (95% CI 57–97%) (Figure 3D, Supplementary Table 7, and Supplementary Figure S1).

### 3.8 NFS

The NFS serological biomarker was evaluated for diagnostic accuracy in detecting AnF (> F1) (5 studies), SF (≥ F2–F4) (14 studies), AF (≥ F3) (43 studies), and cirrhosis (F4) (3 studies) (Supplementary Table S7).

#### 3.8.1 Diagnosis of AnF (F0 vs. F1–F4)

The DOR of the NFS in the diagnosis of AnF was 4.85 (95% CI 3.32–7.09), the LR+ was 2.27 (95% CI 1.86–2.78), the LR- was 0.49 (95% CI 0.42–0.57), and moderate heterogeneity was detected (Q = 6.63, *p* = 0.15, I^2^ = 39.66%) (Table 2; Supplementary Figures 18, 19). The sAUROC had a moderate diagnostic accuracy of 0.71, Sen of 66% (95% CI 62–70%), and Spe of 73% (95% CI 64–81%) (Figure 3A and Supplementary Table S7, and Supplementary Figure S1).

#### 3.8.2 Diagnosis of SF (F0–F1 vs. F2–F4)

The DOR of the NFS in the diagnosis of SF was 9.45 (95% CI 5.17–17.5), the LR+ was 3.35 (95% CI 2.42–4.63), the LR- was 0.42 (95% CI 0.33–0.54), and low heterogeneity was detected (Q = 13.53, *p* = 0.40, I^2^ = 3.91%) (Table 2; Supplementary Figures S20, S21). The sAUROC had a good diagnostic accuracy of 0.81, Sen of 69% (95% CI 56–79%), and Spe of 80% (95% CI 71–88%) (Figure 3B, Supplementary Table S7, and Supplementary Figure S1).

#### 3.8.3 Diagnosis of AF (F0–F2 vs. F3–F4)

The DOR of the NFS in the diagnosis of AF was 9.74 (95% CI 6.69–14.17), the LR+ was 3.56 (95% CI 2.93–4.32), the LR- was 0.44 (95% CI 0.38–0.51), and no heterogeneity was detected (Q = 37.99, *p* = 0.64, I^2^ = 0%) (Table 2; Supplementary Figures S22, S23). The sAUROC had a good diagnostic accuracy of 0.81, Sen of 62% (95% CI 53–70%), and Spe of 85% (95% CI 79–90%) (Figure 3C, Supplementary Table S7, and Supplementary Figure S1).

#### 3.8.4 Diagnosis of cirrhosis (F0–F3 vs. F4)

The DOR of the NFS in the diagnosis of cirrhosis was 9.13 (95% CI 4.25–19.62), the LR+ was 3.88 (95% CI 2.35–6.39), the LR- was 0.43 (95% CI 0.32–0.58), and no heterogeneity was detected (Q = 1.72, *p* = 0.42, I^2^ = 0%) (Table 2; Supplementary Figures S24, S25). The sAUROC had a moderate diagnostic accuracy of 0.69, Sen of 63% (95% CI 58–68%), and Spe of 84% (95% CI 73–91%) (Figure 3D, Supplementary Table S7, and Supplementary Figure S1).

### 3.9 BARD score

The BARD score serological biomarker was evaluated for diagnostic accuracy in detecting SF (≥ F2–F4) (6 studies) and AF (≥ F3) (21 studies) (Supplementary Table S6).

#### 3.9.1 Diagnosis of SF (F0–F1 vs. F2–F4)

The DOR of the BARD score in the diagnosis of SF was 5.98 (95% CI 2.62–13.66), the LR+ was 2.49 (95% CI 1.72–3.61), the LR- was 0.46 (95% CI 0.30–0.70), and no heterogeneity was detected (Q = 4.11, *p* = 0.53, I^2^ = 0%) (Table 2; Supplementary Figures S26, S27). The sAUROC had a moderate diagnostic accuracy of 0.76, Sen of 63% (95% CI 45–82%), and Spe of 79% (95% CI 65–83%) (Figure 3B, Supplementary Table S7, and Supplementary Figure S1).

#### 3.9.2 Diagnosis of AF (F0–F2 vs. F3–F4)

The DOR of the BARD score in the diagnosis of AF was 4.34 (95% CI 3.40–5.55), the LR+ was 1.88 (95% CI 1.65–2.14), the LR- was 0.48 (95% CI 0.41–0.56), and low heterogeneity was detected (Q = 26.11, *p* = 0.16, I^2^ = 23.4%) (Table 2; Supplementary Figures S28, S29). The sAUROC had a moderate diagnostic accuracy of 0.73, Sen of 72% (95% CI 64–79%), and Spe of 63% (95% CI 54–71%) (Figure 3C, Supplementary Table S7, and Supplementary Figure S1).

### 3.10 FibroMeter

The FibroMeter serological biomarker was evaluated for diagnostic accuracy in detecting SF (≥ F2–F4) (4 studies) and AF (≥ F3) (12 studies) (Supplementary Table S7).

#### 3.10.1 Diagnosis of SF (F0–F1 vs. F2–F4)

The DOR of the FibroMeter in the diagnosis of SF was 17.82 (95% CI 4.91–64.7), the LR+ was 6.00 (95% CI 2.07–3.05), the LR- was 0.35 (95% CI 0.18–0.67), and no heterogeneity was detected (Q = 2.69, *p* = 0.44, I^2^ = 0%) (Table 2; Supplementary Figures S30, S31). The sAUROC had a good diagnostic accuracy of 0.88, Sen of 68% (95% CI 48–82%), and Spe of 89% (95% CI 80–95%) (Figure 3B, Supplementary Table 7, and Supplementary Figure S1).

#### 3.10.2 Diagnosis of AF (F0–F2 vs. F3–F4)

The DOR of the FibroMeter in the diagnosis of AF was 13.72 (95% CI 7.51–25.07), the LR+ was 4.16 (95% CI 2.89–5.99), the LR- was 0.31 (95% CI 0.24–0.40), and no heterogeneity was detected (Q = 9.42, *p* = 0.58, I^2^ = 0%) (Table 2; Supplementary Figures 32, 33). The sAUROC had a good diagnostic accuracy of 0.84, Sen of 74% (95% CI 68–79%), and Spe of 82% (95% CI 76–87%) (Figure 3C, Supplementary Table S7, and Supplementary Figure S1).

### 3.11 FibroTest

The FibroTest serological biomarker was evaluated for diagnostic accuracy in detecting SF (≥ F2–F4) (4 studies) and AF (≥ F3) (6 studies) (Supplementary Table S7).

#### 3.11.1 Diagnosis of SF (F0–F1 vs. F2–F4)

The DOR of the FibroTest in the diagnosis of SF was 5.19 (95% CI 1.77–15.18), the LR+ was 2.10 (95% CI 1.36–3.25), the LR- was 0.56 (95% CI 0.36–0.85), and high heterogeneity was detected (Q = 12.21, *p* = 0.007, I^2^ = 75.42%) (Table 2; Supplementary Figures 34, S35). The sAUROC had a good diagnostic accuracy of 0.86, Sen of 72% (95% CI 28–94%), and Spe of 85% (95% CI 45–98%) (Figure 3B, Supplementary Table S7, and Supplementary Figure S1).

#### 3.11.2 Diagnosis of AF (F0–F2 vs. F3–F4)

The DOR of the FibroTest in the diagnosis of AF was 7.45 (95% CI 5.15–10.77), the LR+ was 3.81 (95% CI 2.18–6.64), the LR- was 0.58 (95% CI 0.43–0.79), and no heterogeneity was detected (Q = 4.48, *p* = 0.48, I^2^ = 0%) (Table 2; Supplementary Figures S36, S37). The sAUROC had a moderate diagnostic accuracy of 0.78, Sen of 40% (95% CI 15–72%), and Spe of 93% (95% CI 73–99%) (Figure 3C, Supplementary Table S7, and Supplementary Figure S1).

### 3.12 ELF

The ELF serological biomarker was evaluated for diagnostic accuracy in detecting AF (≥ F3) (6 studies) (Supplementary Table S7).

#### 3.12.1 Diagnosis of AF (F0–F2 vs. F3–F4)

The DOR of the ELF in the diagnosis of AF was 18.82 (95% CI 9.52–37.18), the LR+ was 4.42 (95% CI 3.12–6.25), the LR- was 0.29 (95% CI 0.23–0.38), and low heterogeneity was detected (Q = 7.05, *p* = 0.21, I^2^ = 29.08%) (Table 2; Supplementary Figures S38, S39). The sAUROC had a good diagnostic accuracy of 0.87, Sen of 79% (95% CI 68–87%), and Spe of 84% (95% CI 75–90%) (Figure 3C, Supplementary Table S7, and Supplementary Figure S1).

### 3.13 Sensitivity analysis

The sensitivity analysis showed that there were no changes in the results when only tests with more than 40% of participants (APRI, FIB-4, NFS, and BARD score) and severities (SF, AF, and cirrhosis) were included (Supplementary Figures S40–S58; Supplementary Table S8).

## 4 Discussion

This systematic review and meta-analysis aimed to assess the accuracy of different prognostic serological biomarkers in predicting liver fibrosis severity in people with MASLD. The serological biomarkers varied according to the different degrees of severity of liver fibrosis. For any type of fibrosis, all the models had moderate precision. For significant fibrosis, the FibroMeter, FibroTest, and NFS models had high precision, and APRI, FIB-4, and BARD score had moderate precision. For advanced fibrosis, the ELF, FibroMeter, FIB-4, and NFS models had high precision, and BARD score, FibroTest, and APRI presented moderate precision. Finally, for cirrhosis, only FIB-4 showed high precision, while APRI and NFS had moderate diagnostic precision in the evaluation of this severity.

The APRI showed moderate diagnostic accuracy across all degrees of liver fibrosis severity, from AnF to cirrhosis, the results that are consistent with previous meta-analyses reporting moderate accuracy in assessing AF with this prognostic model. In addition, different studies have reported inconsistencies in predicting liver fibrosis using this score (8, 96). Therefore, due to conflicting results regarding the effectiveness of the APRI score, the MASLD practice guideline of the AASLD, American College of Gastroenterology, and American Gastroenterological Association recommends using the FIB-4 or NFS score to identify patients with MASLD with stage 3 or 4 fibrosis (6). Our results support this recommendation as FIB-4 and NFS showed good diagnostic accuracy in the assessment of liver fibrosis severity, for SF and AF, and AF and cirrhosis, respectively.

As science has advanced, several serum tests have been developed using either direct biomarkers (reflecting the pathophysiology of hepatic fibrogenesis) or indirect biomarkers (reflecting functional changes in the liver) alone or in combination (57). Complex panels (such as FibroMeter and ELF) have been shown to be more accurate and reproducible for detecting AF than simple panels (159). Our results support these findings, suggesting that both models have good diagnostic accuracy for AF, whereas simple panels such as APRI and BARD score, although cheaper, easier to calculate, and widely available, are not as accurate as complex panels (159).

Different studies have consistently reported that the ELF model provides good results in the assessment of AF, including the 2021 National Institute of Health and Care Excellence guidelines, which established that for the assessment and treatment of people with MASLD, the ELF score is considered “the most cost-effective and appropriate test for AF in adults with MASLD” (160). However, the reality of clinical practice is different as the ELF score is not accessible to frontline health professionals, which may represent a barrier to the detection of liver fibrosis (9, 57).

The FibroTest also showed good diagnostic performance for the assessment of SF in this review. FibroTest and FibroMeter are models that include the analysis of extracellular matrix substances directly involved in the progression of fibrosis and have better Sen and Spe, suggesting that the inclusion of a direct marker of liver fibrosis in a non-invasive test can improve its diagnostic accuracy (8, 9).

Another relevant result was that only three models detected AnF: APRI, FIB-4, and NFS. These models are considered simple scores, that is, none of the complex models analyzed in this review identified this severity. Therefore, there is still a lack of studies evaluating any of these models in the assessment of AnF as most scores have focused on the importance of histological determinants of severe fibrosis and its relevance in the development of future disease. However, the identification of AnF in community settings will allow for the implementation of early lifestyle interventions and consequently inform the decision to refer to secondary care in severe cases (62, 134).

MASLD is also strongly correlated with MetS. Of the 138 included studies, 54.6% reported at least some component of this syndrome. Two recent reviews have suggested that MASLD is both a cause and a consequence of MetS (161, 162). This is because liver fat is presented as a marker of metabolic abnormalities that characterize MetS, and the possibility of MASLD should be considered in all patients diagnosed with MetS with any of the different sets of criteria (161, 162). In the present review, the mean values for both transaminases were above normal, indicating that the studies were conducted in populations with at least some alteration in the serological tests of the liver. In people with MASLD with normal transaminase levels, 16–24% of them may have AF, with the sAUROC for the BARD score, FIB-4, and NFS ranging from 0.71 to 0.85 (99, 152).

In this review, we found a mean BMI of 32.8 kg/m^2^ in the total study population, which is considered grade-I obesity. The findings of a meta-analysis suggest that there is evidence of a high predictive value of abdominal obesity as an indicator of increased risk of metabolic disorders and cardiovascular disease, as well as evidence supporting the cause-and-effect relationship between abdominal obesity and MASLD (163). A recent review showed that there is less evidence when evaluating the tests in populations of patients with obesity, and non-invasive tests tend to be less favorable in these populations due to differences in terms of BMI and alanine aminotransferase levels, which may mean that serum-based scores derived from the liver clinical setting in groups with different hepatic risk profiles do not adequately reflect the accuracy of these tests in the obese population (9).

Conversely, the present results of prognostic models showing moderate diagnostic accuracy may also be related to the fact that this meta-analysis included a larger number of studies, heterogeneous populations and their variables, and all degrees of fibrosis severity compared to previous meta-analyses (9, 10). Although the objective of non-invasive models is not to replace the biopsy, our results highlight the importance of using these models in the evaluation of MALSD patients with suspected liver fibrosis, which determines the prognosis of the disease, as well as the usefulness and feasibility of performing these tests, given the lack of other methods in primary care for these patients (159).

## 5 Limitations

However, our meta-analysis has limitations. First of all, there was no stratification of the different models by age, race, weight, and morbidities, only by stages of fibrosis, since few studies were conducted in clinical trials to compare homogeneous populations. Another limitation of the present study is the non-inclusion of imaging biomarkers such as MRE. The decision not to include these biomarkers was made to focus on the serological biomarkers recommended by the guidelines to provide a more comprehensive assessment of their performance. However, this is a study with a large sample of participants, with low heterogeneity between the different studies, which aims to contribute to the generalization of results based on possible limitations in health services.

## 6 Conclusion

The findings of this meta-analysis suggest that when comparing the scores of serological biomarkers with liver biopsies for predicting liver fibrosis severity in people with MASLD, the FIB-4 has good predictive diagnostic accuracy for any fibrosis, the FibroMeter has good predictive diagnostic accuracy for significant fibrosis, the ELF has good predictive diagnostic accuracy for advanced fibrosis, and the FIB-4 has good diagnostic accuracy for cirrhosis. These non-invasive serological biomarkers can thus be considered as an alternative to determine the prognosis of this disease.

## Data availability statement

The original contributions presented in the study are included in the article/Supplementary material, further inquiries can be directed to the corresponding author.

## Author contributions

SL: Conceptualization, Formal analysis, Methodology, Writing – original draft, Writing – review & editing. CA: Data curation, Formal analysis, Methodology, Writing – original draft, Writing – review & editing. PR: Data curation, Writing – original draft. CC: Writing – review & editing, Writing – original draft. MW: Formal analysis, Software, Writing – review & editing, Writing – original draft. AP: Conceptualization, Writing – review & editing, Funding acquisition, Writing – original draft. RM: Conceptualization, Formal analysis, Investigation, Methodology, Writing – original draft, Writing – review & editing.

## Figures and Tables

**Table:** 

95% CI	95% confidence interval
95% UI	95% uncertainty interval
AASLD	American Association for the Study of Liver Diseases
AF	Advanced fibrosis
ALT	Alanine transaminase
AnF	Any fibrosis
APRI	Aspartate aminotransferase-to-platelet ratio
AST	Aspartate aminotransferase
AST/ALT ratio	Aspartate aminotransferase/alanine aminotransferase ratio
AUC	Area under curve
AUROC	Area under the receiver operating characteristic
BARD score	Body mass index, aspartate aminotransferase/alanine aminotransferase ratio, diabetes score
BMI	Body mass index
CINAHL	Cumulative Index to Nursing and Allied Health Literature
DOR	Diagnostic odds ratio
EASL	European Association for the Study of the Liver
ELF	Enhanced liver fibrosis
EMBASE	Excerpt Medical dataBASE
FN	False negatives
FP	False positives
HbA1C	Glycosylated hemoglobin
kg	Kilograms
LILACS	Latin American and Caribbean Health Sciences Literature
LR-	Negative likelihood ratio
LR+	Positive likelihood ratio
LSM-VCTE	Liver stiffness measurement by vibration-controlled transient elastography
m^2^	Meters^2^
MADA	Meta-analysis of diagnostic accuracy
MASLD	Metabolic dysfunction-associated steatotic liver disease
MEDLINE	Medical Literature Analysis and Retrieval System Online
MetS	Metabolic syndrome
MRE	Magnetic resonance elastography
NFS	Non-alcoholic fatty liver disease fibrosis score
PIT	Participants, index tests, and target condition
PRISMA-DTA	Preferred Reporting Items for Systematic Reviews and Meta-Analyses of Diagnostic Test Accuracy Studies
PROSPERO	International Prospective Register of Systematic Reviews database
PUBMED	Public/Publisher MEDLINE
Q	Cochran’s Q
QUADAS-2	Quality Assessment of Diagnostic Accuracy Studies-2
sAUROC	Summary area under the receiver operating characteristic
SciELO	Scientific Electronic Library Online
Sen	Sensitivity
SF	Significant fibrosis
Spe	Specificity
SROC	Summary receiver operator characteristic
SWE	Shear wave elastography
TN	True negatives
TP	True positives
WOS	Web of Science

## Footnotes

^1^https://CRAN.R-project.org/package=mada

^2^http://araw.mede.uic.edu/cgi-bin/testcalc.pl
