# Supplementary material for: Accuracy of prognostic serological biomarkers in predicting liver fibrosis severity in people with metabolic dysfunction-associated steatotic liver disease: a meta-analysis of over 40,000 participants
Source: Front Nutr. 2024 Feb 14;11:1284509. doi: 10.3389/fnut.2024.1284509 (PMC10899345; doi:10.3389/fnut.2024.1284509)
Supplement: Supplementary file 1 [file Data_Sheet_1.docx]

**Accuracy of prognostic serological biomarkers in predicting liver fibrosis severity in people with** **metabolic dysfunction-associated steatotic liver disease: a meta-analysis of over 40,000 participants.**

**Sergio M. López Tórrez, Camila O. Ayala, Paula Bayer Ruggiro, Caroline Abud Drumond Costa, Mario B Wagner, Alexandre Vontobel Padoin, Rita Mattiello***

***Correspondence:** Rita Mattiello: [rita.mattiello@ufrgs.br](mailto:rita.mattiello@ufrgs.br)

| **Supplementary Table 1. PRISMA-DTA Checklist Item** | | | |
| --- | --- | --- | --- |
| **Section/topic** | **#** | **PRISMA-DTA Checklist Item** | **Reported on page #** |
| **TITLE / ABSTRACT** | | |  |
| Title | 1 | Identify the report as a systematic review (+/- meta-analysis) of diagnostic test accuracy (DTA) studies. | 1 |
| Abstract | 2 | Abstract: See PRISMA-DTA for abstracts. | 2 to 3 |
| **INTRODUCTION** | | |  |
| Rationale | 3 | Describe the rationale for the review in the context of what is already known. | 3 |
| Clinical role of index test | D1 | State the scientific and clinical background, including the intended use and clinical role of the index test, and if applicable, the rationale for minimally acceptable test accuracy (or minimum difference in accuracy for comparative design). | 3 to 5 |
| Objectives | 4 | Provide an explicit statement of question(s) being addressed in terms of participants, index test(s), and target condition(s). | 5 |
| **METHODS** | | |  |
| Protocol and registration | 5 | Indicate if a review protocol exists, if and where it can be accessed (e.g., Web address), and, if available, provide registration information including registration number. | 5 |
| Eligibility criteria | 6 | Specify study characteristics (participants, setting, index test(s), reference standard(s), target condition(s), and study design) and report characteristics (e.g., years considered, language, publication status) used as criteria for eligibility, giving rationale. | 5 to 6 |
| Information sources | 7 | Describe all information sources (e.g., databases with dates of coverage, contact with study authors to identify additional studies) in the search and date last searched. | 6 |
| Search | 8 | Present full search strategies for all electronic databases and other sources searched, including any limits used, such that they could be repeated. | 6 |
| Study selection | 9 | State the process for selecting studies (i.e., screening, eligibility, included in systematic review, and, if applicable, included in the meta-analysis). | 7 |
| Data collection process | 10 | Describe method of data extraction from reports (e.g., piloted forms, independently, in duplicate) and any processes for obtaining and confirming data from investigators. | 7 |
| Definitions for data extraction | 11 | Provide definitions used in data extraction and classifications of target condition(s), index test(s), reference standard(s) and other characteristics (e.g. study design, clinical setting). | 7 |
| Risk of bias and applicability | 12 | Describe methods used for assessing risk of bias in individual studies and concerns regarding the applicability to the review question. | 7 to 8 |
| Diagnostic accuracy measures | 13 | State the principal diagnostic accuracy measure(s) reported (e.g. sensitivity, specificity) and state the unit of assessment (e.g. per-patient, per-lesion). | 5 to 8 |
| Synthesis of results | 14 | Describe methods of handling data, combining results of studies and describing variability between studies. This could include, but is not limited to: a) handling of multiple definitions of target condition. b) handling of multiple thresholds of test positivity, c) handling multiple index test readers, d) handling of indeterminate test results, e) grouping and comparing tests, f) handling of different reference standards | 8 to 9 |

| **Section/topic** | **#** | **PRISMA-DTA Checklist Item** | **Reported on page #** |
| --- | --- | --- | --- |
| Meta-analysis | D2 | Report the statistical methods used for meta-analyses, if performed. | 8 to 9 |
| Additional analyses | 16 | Describe methods of additional analyses (e.g., sensitivity or subgroup analyses, meta-regression), if done, indicating which were pre-specified. | 8 to 9 |
| **RESULTS** | | |  |
| Study selection | 17 | Provide numbers of studies screened, assessed for eligibility, included in the review (and included in meta-analysis, if applicable) with reasons for exclusions at each stage, ideally with a flow diagram. | 9 |
| Study characteristics | 18 | For each included study provide citations and present key characteristics including: a) participant characteristics (presentation, prior testing), b) clinical setting, c) study design, d) target condition definition, e) index test, f) reference standard, g) sample size, h) funding sources | 10 to 12 |
| Risk of bias and applicability | 19 | Present evaluation of risk of bias and concerns regarding applicability for each study. | 12 |
| Results of individual studies | 20 | For each analysis in each study (e.g. unique combination of index test, reference standard, and positivity threshold) report 2x2 data (TP, FP, FN, TN) with estimates of diagnostic accuracy and confidence intervals, ideally with a forest or receiver operator characteristic (ROC) plot. | 13 to 18 |
| Synthesis of results | 21 | Describe test accuracy, including variability; if meta-analysis was done, include results and confidence intervals. | 13 to 18 |
| Additional analysis | 23 | Give results of additional analyses, if done (e.g., sensitivity or subgroup analyses, meta-regression; analysis of index test: failure rates, proportion of inconclusive results, adverse events). | 19 |
| **DISCUSSION** | | |  |
| Summary of evidence | 24 | Summarize the main findings including the strength of evidence. | 19 to 22 |
| Limitations | 25 | Discuss limitations from included studies (e.g. risk of bias and concerns regarding applicability) and from the review process (e.g. incomplete retrieval of identified research). | 22 |
| Conclusions | 26 | Provide a general interpretation of the results in the context of other evidence. Discuss implications for future research and clinical practice (e.g. the intended use and clinical role of the index test). | 23 |
| **FUNDING** | | |  |
| Funding | 27 | For the systematic review, describe the sources of funding and other support and the role of the funders. | 25 |

# **Supplementary Text 1.** Electronic search strategy.

**MEDLINE/PubMed**

#1. “Fatty liver” [MeSH]

#2. liver, Fatty***** [Title/Abstract]

#3. Steatohepatitis***** [Title/Abstract]

#4. Steatohepatitides***** [Title/Abstract]

#5. Steatosis of Liver*****[Title/Abstract]

#6. Liver Steatosis*****[Title/Abstract]

#7. Liver Steatoses***** [Title/Abstract]

#8. Steatoses, Liver***** [Title/Abstract]

#9. Steatosis, Liver***** [Title/Abstract]

#10. Hepatosteatosis***** [Title/Abstract]

#11. #1 OR #2 OR #3 OR #4 OR #5 OR #6 OR #7 OR #8 OR #9 OR #10

#12. “Non-alcoholic Fatty Liver Disease” [MeSH]

#13. Non alcoholic Fatty Liver Disease***** [Title/Abstract]

#14. Nonalcoholic Fatty Liver Disease***** [Title/Abstract]

#15. Fatty Liver, Nonalcoholic***** [Title/Abstract]

#16. Fatty Livers, Nonalcoholic***** [Title/Abstract]

#17. Liver, Nonalcoholic Fatty***** [Title/Abstract]

#18. Livers, Nonalcoholic Fatty***** [Title/Abstract]

#19. Nonalcoholic Fatty Liver***** [Title/Abstract]

#20. Nonalcoholic Fatty Livers***** [Title/Abstract]

#21. Nonalcoholic Steatohepatitis***** [Title/Abstract]

#22. Steatohepatitis, Nonalcoholic***** [Title/Abstract]

#23. NALFD***** [Title/Abstract]

#24. NASH***** [Title/Abstract]

#25. Metabolic-Associated Fatty Liver Disease***** [Title/Abstract]

#26. MAFLD***** [Title/Abstract]

#27. #12 OR #13 OR #14 OR #15 OR #16 OR #17 OR #18 #19 OR #20 OR #21

OR #22 OR #23 OR #24 OR #25 OR #26

#28. #11 OR #27

#29. “Biopsy” [MeSH]

#30. Biopsies***** [Title/Abstract]

#31. #29 OR #30

#32. “Liver Cirrhosis” [MeSH]

#33. Hepatic Cirrhosis***** [Title/Abstract]

#34. Hepatic Cirrhoses***** [Title/Abstract]

#35. Cirrhosis, Hepatic***** [Title/Abstract]

#36. Cirrhosis, Liver***** [Title/Abstract]

#37. Cirrhoses, Liver***** [Title/Abstract]

#38. Fibrosis, Liver***** [Title/Abstract]

#39. Fibroses, Liver***** [Title/Abstract]

#40. Liver Fibrosis***** [Title/Abstract]

#41. Liver Fibroses***** [Title/Abstract]

#42. Cirrhosis***** [Title/Abstract]

#43. #32 OR #33 OR #34 OR #35 OR #36 OR #37 OR #38 OR #39 OR #40 OR #41 OR #42

#44. “Prognosis” [MeSH]

#45. Prognoses***** [Title/Abstract]

#46. Predict***** [Title/Abstract]

#47. Course***** [Title/Abstract]

#48. “ROC curve” [MeSH]

#49. Curves, ROC***** [Title/Abstract]

#50. Receiver Operating Characteristic***** [Title/Abstract]

#51. Stratification***** [Title/Abstract]

#52. Discrimination***** [Title/Abstract]

#53. c-statistic***** [Title/Abstract]

#54. Area under the curve***** [Title/Abstract]

#55. Curve, Area Under***** [Title/Abstract]

#56. AUC***** [Title/Abstract]

#57. “Calibration” [MeSH]

#58. Indices***** [Title/Abstract]

#59. Algorithm***** [Title/Abstract]

#60. #44 OR #45 OR #46 OR #47 OR #48 OR #49 #50 OR #51 OR #52 OR #53 OR #54 OR #55 OR #56 OR #57 OR #58 OR #59

#61 FibroTest

#62. FibroMeter

#63. Enhanced liver fatty

#64. ELF

#65. NAFLD fibrosis score

#66. FIB-4 index

#67. BARD score

#68. APRI

#69. Hepascore

#70. FORN score

#71. FIBROSpect test

#72. #61 OR #62 OR #63 OR #64 OR #65 OR #65 OR # 66 OR #67 OR #68 OR #69 OR #71 OR #72

#73. #28 AND #31 AND #43 AND #60 AND #72

**EMBASE**

#1. ‘Fatty liver’

#2. ‘liver, Fatty’

#3. ‘Steatohepatitis’

#4. ‘Steatohepatitides’

#5. ‘Liver Steatosis’

#6. ‘Liver Steatoses’

#7. ‘Steatosis, Liver’

#8. ‘Hepatosteatosis’

#9. OR/ #1 - #9

#10. ‘nonalcoholic fatty liver’

#11. ‘NAFLD (nonalcoholic fatty liver disease)’

#12. ‘non alcoholic fatty liver disease’

#13. ‘non alcoholic hepato-steatosis’

#14. ‘non alcoholic liver steatosis’

#15. ‘non alcoholic steatotic hepatopathy’

#16. ‘nonalcoholic hepatosteatosis’

#17. ‘non-alcoholic FLD’

#18. ‘nonalcoholic FLD’

#19. ‘metabolic fatty liver’

#20. ‘MAFLD (metabolic associated fatty liver disease)’

#21. OR/ #10 - #20

#22. OR/ #9 - #21

#23. ‘Biopsy’

#24. ‘Bioptic diagnosis’

#25. ‘Bioptical diagnosis’

#26. OR/ #23 – #25

#27.  ‘Liver cirrhosis’

#28. ‘Cirrhosis’

#29. ‘Cirrhosis hepatis’

#30. ‘Cirrhosis, liver’

#31. ‘Hepatic cirrhosis’

#32. OR/ #27 – #31

#33. ‘Prognosis’

#34. ‘Prognoses’

#35. ‘Predict’

#36. ‘Course’

#37. ‘receiver operating characteristic’

#38. ‘receiver operating characteristic curve’

#39. ‘ROC curve’

#40. ‘ROC analysis’

#41. ‘Stratification’

#42. ‘Discrimination’

#43. ‘area under the curve’

#44. ‘area under curve’

#45. ‘AUC’

#46. ‘AUC (area under the curve)’

#47. ‘Calibration’

#48. ‘Indices’

#49. ‘Algorithm’

#50. OR/ #33 – #49

#51. ‘FibroTest’

#52. ‘FibroMeter’

#53. ‘Enhanced liver fatty’

#54. ‘ELF’

#55. ‘NAFLD fibrosis score’

#56. ‘FIB-4 index’

#57. ‘BARD score’

#58. ‘APRI’

#59. ‘Hepascore’

#60. ‘FORN score’

#61. ‘FIBROSpect test’

#62. OR/#51 - #61

#63. #22 AND #26 AND #32 AND #50 AND #62

**LILACS**

Fatty liver OR liver, Fatty OR Steatohepatitis OR Steatohepatitides OR Steatosis of Liver OR Liver Steatosis OR Steatoses, Liver OR Steatosis, Liver OR Hepatosteatosis OR Non-alcoholic Fatty Liver Disease OR Nonalcoholic Fatty Liver Disease OR Nonalcoholic Fatty Liver Disease Nonalcoholic Steatohepatitis OR Steatohepatitis, Nonalcoholic OR NALFD OR NASH OR Metabolic-Associated Fatty Liver Disease OR MAFLD

**AND**

Biopsy OR Biopsies

**AND**

Liver Cirrhosis OR Hepatic Cirrhosis OR Cirrhosis, Hepatic OR Cirrhosis, Liver OR Fibrosis, Liver OR Liver Fibrosis OR Liver Fibroses OR Cirrhosis

**AND**

Prognosis OR Prognoses OR Predict OR Course OR Receiver Operating Characteristic OR ROC curve OR Curves, ROC OR Stratification OR Discrimination OR c-statistic OR Area under the curve OR Curve, Area Under OR Calibration OR Indices OR Algorithm

**AND**

FibroTest OR FibroMeter OR Enhanced liver fatty OR ELF OR NAFLD fibrosis score OR FIB-4 index OR BARD score OR APRI OR Hepascore OR FORN score OR FIBROSpect test

**CINAHL**

Fatty liver

OR

liver, Fatty

OR

Steatohepatitis

OR

Steatohepatitides

OR

Steatosis of Liver

OR

Liver Steatosis

OR

Liver Steatoses

OR

Steatoses, Liver

OR

Steatosis, Liver

OR

Hepatosteatosis

AND

Non-alcoholic Fatty Liver Disease

OR

Non alcoholic Fatty Liver Disease

OR

Nonalcoholic Fatty Liver Disease

OR

Fatty Liver, Nonalcoholic

OR

Fatty Livers, Nonalcoholic

OR

Liver, Nonalcoholic Fatty

OR

Livers, Nonalcoholic Fatty

OR

Nonalcoholic Fatty Liver

OR

Nonalcoholic Fatty Livers

OR

Nonalcoholic Steatohepatitis

OR

Steatohepatitis, Nonalcoholic

OR

NALFD

OR

NASH

Metabolic-Associated Fatty Liver Disease

OR

MAFLD

AND

Biopsy

OR

Biopsies

AND

Liver Cirrhosis

OR

Hepatic Cirrhosis

OR

Cirrhosis, Hepatic

OR

Cirrhosis, Liver

OR

Fibrosis, Liver

OR

Liver Fibrosis

OR

Liver Fibroses

OR

Cirrhosis

AND

Prognosis

OR

Prognoses

OR

Predict

OR

Course

OR

Receiver Operating Characteristic

OR

ROC curve

OR

Curves, ROC

OR

Stratification

OR

Discrimination

OR

c-statistic

OR

Area under the curve

OR

Curve, Area Under

OR

AUC

OR

Calibration

OR

Indices

OR

Algorithm

AND

FibroTest

OR

FibroMeter

OR

Enhanced liver fatty

OR

ELF

OR

NAFLD fibrosis score

OR

FIB-4 index

OR

BARD score

OR

APRI

OR

Hepascore

OR

FORN score

OR

FIBROSpect test

**Cochrane Hepato-Biliary Group Diagnostic Test Accuracy Studies Register**

**Title, abstract, keyword:** Fatty liver OR liver, Fatty OR Steatohepatitis OR Steatohepatitides OR Steatosis of Liver OR Liver Steatosis OR Steatoses, Liver OR Steatosis, Liver OR Hepatosteatosis OR Non-alcoholic Fatty Liver Disease OR Nonalcoholic Fatty Liver Disease OR Nonalcoholic Fatty Liver Disease OR Fatty Liver, Nonalcoholic OR Fatty Livers, Nonalcoholic ORLiver, Nonalcoholic Fatty OR Livers, Nonalcoholic Fatty OR Nonalcoholic Fatty Liver OR Nonalcoholic Fatty Livers OR Nonalcoholic Steatohepatitis OR Steatohepatitis, Nonalcoholic OR NALFD OR NASH Metabolic-Associated Fatty Liver Disease OR MAFLD

**AND**

**Title, abstract, keyword:** Biopsy OR Biopsies

**AND**

**Title, abstract, keyword:** Liver Cirrhosis OR Hepatic Cirrhosis OR Cirrhosis, Hepatic OR Cirrhosis, Liver OR Fibrosis, Liver OR Liver Fibrosis OR Liver Fibroses OR Cirrhosis AND prognosis OR Prognoses OR Predict OR Course OR Receiver Operating Characteristic OR ROC curve OR Curves, ROC OR Stratification OR Discrimination OR c-statistic OR Area under the curve OR Curve, Area Under OR Calibration OR Indices OR Algorithm

**AND**

**Title, abstract, keyword:** FibroTest OR FibroMeter OR Enhanced liver fatty OR ELF OR NAFLD fibrosis score OR FIB-4 index OR BARD score OR APRI OR Hepascore OR FORN score OR FIBROSpect test

**Web of Science**

1. TS= (Fatty liver)
2. TS= (liver, Fatty)
3. TS= (Steatohepatitis)
4. TS= (Steatohepatitides)
5. TS= (Steatosis of Liver)
6. TS= (Liver Steatosis)
7. TS= (Liver Steatoses)
8. TS= (Steatoses, Liver)
9. TS= (Steatosis, Liver)
10. TS= (Hepatosteatosis)
11. OR/ 1 – 10
12. TS= (Non-alcoholic Fatty Liver Disease)
13. TS= (Non alcoholic Fatty Liver Disease)
14. TS= (Nonalcoholic Fatty Liver Disease)
15. TS= (Fatty Liver, Nonalcoholic)
16. TS= (Fatty Livers, Nonalcoholic)
17. TS= (Liver, Nonalcoholic Fatty)
18. TS= (Livers, Nonalcoholic Fatty)
19. TS= (Nonalcoholic Fatty Liver)
20. TS= (Nonalcoholic Fatty Livers)
21. TS= (Nonalcoholic Steatohepatitis)
22. TS= 9Steatohepatitis, Nonalcoholic)
23. TS= (NALFD)
24. TS= (NASH)
25. TS= (Metabolic-Associated Fatty Liver Disease)
26. TS= (MAFLD)
27. OR/ 12 – 26
28. OR/ 11 – 27
29. TS= (Biopsy)
30. TS= (Biopsies)
31. OR/ 29 – 30
32. TS= (Liver Cirrhosis)
33. TS= (Hepatic Cirrhosis)
34. TS= (Cirrhosis, Hepatic)
35. TS= (Cirrhosis, Liver)
36. TS= (Fibrosis, Liver)
37. TS= (Liver Fibrosis)
38. TS= (Liver Fibroses)
39. TS= (Cirrhosis)
40. OR/ 32 – 39
41. TS= (Prognosis)
42. TS= (Prognoses)
43. TS= (Predict)
44. TS= (Course)
45. TS= (Receiver Operating Characteristic)
46. TS= (ROC curve)
47. TS= (Curves, ROC)
48. TS= (Stratification)
49. TS= (Discrimination)
50. TS= (c-statistic)
51. TS= (Area under the curve)
52. TS= (Curve, Area Under)
53. TS= (AUC)
54. TS= (Calibration)
55. TS= (Indices)
56. TS= (Algorithm)
57. OR/ 41 – 56
58. TS= (FibroTest)
59. TS= (FibroMeter)
60. TS= (Enhanced liver fatty)
61. TS= (ELF)
62. TS= (NAFLD fibrosis score)
63. TS= (FIB-4 index)
64. TS= (BARD score)
65. TS= (APRI)
66. TS= (Hepascore)
67. TS= (FORN score)
68. TS= (FIBROSpect test)
69. OR/ 58-68
70. 28 AND 31 AND 40 AND 57 AND 69

**SciELO**

Fatty liver OR liver, Fatty OR Steatohepatitis OR Steatohepatitides OR Steatosis of Liver OR Liver Steatosis OR Steatoses, Liver OR Steatosis, Liver OR Hepatosteatosis OR Non-alcoholic Fatty Liver Disease OR Nonalcoholic Fatty Liver Disease OR Nonalcoholic Fatty Liver Disease OR Fatty Liver, Nonalcoholic OR Fatty Livers, Nonalcoholic ORLiver, Nonalcoholic Fatty OR Livers, Nonalcoholic Fatty OR Nonalcoholic Fatty Liver OR Nonalcoholic Fatty Livers OR Nonalcoholic Steatohepatitis OR Steatohepatitis, Nonalcoholic OR NALFD OR NASH OR Metabolic-Associated Fatty Liver Disease OR MAFLD

**AND**

Biopsy OR Biopsies

**AND**

Liver Cirrhosis OR Hepatic Cirrhosis OR Cirrhosis, Hepatic OR Cirrhosis, Liver OR Fibrosis, Liver OR Liver Fibrosis OR Liver Fibroses OR Cirrhosis AND prognosis OR Prognoses OR Predict OR Course OR Receiver Operating Characteristic OR ROC curve OR Curves, ROC OR Stratification OR Discrimination OR c-statistic OR Area under the curve OR Curve, Area Under OR Calibration OR Indices OR Algorithm

**AND**

FibroTest OR FibroMeter OR Enhanced liver fatty OR ELF OR NAFLD fibrosis score OR FIB-4 index OR BARD score OR APRI OR Hepascore OR FORN score OR FIBROSpect test

# **Supplementary Text 2.** Characteristic description of the studies in Table 1

The characteristics of the included studies of the systematic review were published between 2004(1) and 2021 (2–4). Of these, the majority were cross-sectional (68%) (2,3,5–86). The period of execution of the study varied from 1995 (4) to 2019 (84), regarding the number of institutions involved, 71% of the studies were carried out in only one intuition (1–3,6–10,12,13,15,17,18,21–25,27–31,33–37,39–44,46,47,49,50,52,53,55–58,60,62–66,68,69,72–74,77,80–86,88,89,92,94–96,100,101,103–113,115–122) and the remaining 39% were varied institutions (4,5,11,14,16,19,20,26,32,38,48,51,54,59,61,67,70,71,75,76,78,87,90,91,93,97–99,102,114,123–126).

In relation to race, most of the studies were carried out in the Caucasian population 56.11% (1,4,5,7–9,11,13–17,19,21–24,26,28,31,34–36,38,40–42,45,47–51,55,57,59,61,62,65,67,70,71,73,75,76,80,82,83,85,86,88–102,113,115–117,119–121,123,124,127–130) followed in the Asian population 28% (6,14,20,25,27,30,33,39,43,44,46,53,54,56,58,60,64,66,69,71,72,74,77,78,84,103–110,131–133) Hispanic 10.7% (3,10,12,18,32,41,52,63,68,70,71,75,81,134) African 1.43 % (125,129).

Regarding the components of the metabolic syndrome, at least one of these was registered in 55.40% of the studies (6,7,9,11–14,16–18,20–22,25,26,28–35,38,41,42,44,48,49,51–53,55,56,58–62,64–67,70,71,74–78,80,83,85,86,88,90,91,95,97,99,105,108–110,113,118,123,127,129,130,132,134–137), systemic arterial hypertension in 40% of the studies in the range was (15.8% (33)-89.1% (132)), (6,7,13,14,17,20,21,28–30,33,35,44–46,48,52,53,55,58,60,62,64,66,67,70,74,75,77,78,82–87,95,110,113,115,118,127,132,134–138) diabetes mellitus type 2 in 61.80% in the range was (8.4 (23)-100% (85,88)), (3,4,6,7,9,11–14,16–18,21–23,25,26,28–35,38,41–49,51–53,55,56,58,60–62,65–68,70,71,74–78,80,82–88,90,95,97,99,105,108–110,112,113,115,118,123,127,129,132,134,136–138) and dyslipidemia in 26.60% in the ranged was (9 to 96%) (6,21,28,34,48,52,58,66,70,87,105,113,115,127,130,132,135,136). AST was reported in 60.40% (84) of the studies, (2,3,6,7,9,12,13,16,19,21–23,26–29,31–34,36,38–43,45,46,48,51–55,58,60–62,64–67,70–75,80–84,86,87,90,94,95,105,118,123,127,129–132,134,136–138) mean and standard deviation values ranged from (22.3±9 (52) to 79.6±58 (19)), alanine transaminase was evaluated in 62.50% of the studies, (2–4,6,7,9,12–14,16,18,19,21–23,25,27–29,31–34,36,38–43,45–49,51–56,59–62,64–67,69–78,80,82,84–87,90,94,95,105,109,110,118,122,123,127,129–132,134,136–138) with mean and standard deviation values ranged from (23.9±12 (52) to 135.5±96 (36)), the aspartate aminotransferase/alanine aminotransferase ratio was evaluated in 46% of the studies, (2,3,6,7,13,16,18,20–23,25,26,28,29,31,33,34,36,38–42,45,47–49,51,55,56,58,60–62,64–66,71–78,80,82,84–87,94,105,110,118,127,130,132,136–138) with mean and standard deviation values ranged from (0.48±0.37 (118) to 1±0.9 (109)), the platelets was evaluated in 56.80% of the studies, (2,3,6,16,21,22,27,28,33,34,39–43,48,51,52,58,66,67,71,72,80,87,90,105,118,127,130,131,134,136,137) with mean and standard deviation values ranged from (153±65 (27) to 305±90 (86)), in the glycoxylated hemoglobin registered in the 7.20% of the studies,(7,13,23,28,39,41,42,56,64,65,67–71,82–85,95,109,123,129,132,136) with mean and standard deviation values ranged from (0.4±0.37 (118) to 14 ±5(7)), glycemia was reported in the 28.70% of the studies, (6,13,14,16,21,23,28,32,33,38,39,41,42,45,48,49,55,56,59,62,69,73–75,78,82–87,95,105,110,123,127,131,132,136,138) with mean and standard deviation values ranged from (85.4±20 (23) to 148±42 (123)), in the triglycerides was reported in the 40% of the studies, (6,7,9,14,16,21,23,27,29,31,33,36,38,41–46,48,49,53,56,58,59,62,64,65,69,73,78,81–87,95,105,109,110,127,129,131,132,134,136–138) with mean and standard deviation values ranged from (85.07±123 (14) to 239±1.94 (9)), and finally cholesterol was evaluated in the 31.60% of the studies, (6,7,9,14,21,23,27,29,31,33,38,39,41–46,48,49,54,56,58,62,65,69,74,78,82–87,105,109,110,127,129,131,132,136–138) with mean and standard deviation values ranged from (228±58 (87) to 92.6±58 (110)).

| **Supplementary Table 2.** Characteristics of studies included | | | | | | | | | | | | | | | | |
| --- | --- | --- | --- | --- | --- | --- | --- | --- | --- | --- | --- | --- | --- | --- | --- | --- |
| **First author (year of publication) ^ref^** | **Study design** | **Period of Study** | **Institution** | **Race %** | **HTN%** | **DM (%)** | **DLD (%)** | **MetS** | **AST(SD)** | **ALT(SD)** | **AST/ALT ratio (SD)** | **Platelet**  **(SD)** | **HbA1C (%)** | **Glycemia (SD)** | **Triglycerides** | **Cholesterol** |
| Abe M, 2014(131) | Cross-sectional | 2003-2013 | Multiple | Asian | ? | ? | ? | ? | 61.4±48 | 85.5±68 | ? | 189±68 | ? | 115±38 | 144.4±77 | 195.4±41 |
| Adams L, 2008(112) | ? | ? | One | ? | ? | 32 | ? | ? | ? | ? | ? | ? | ? | ? | ? | ? |
| Adams L, 2011(16) | Cross-sectional | ? | Multiple | Caucasian | ? | 24.8 | ? | Yes | 37±54 | 66.5±88 | 0.55 | 233.6±76 | ? | 97±79 | 141±20 | ? |
| Ahmed Z, 2016(37) | Cross-sectional | ? | One | ? | ? | ? | ? | ? | ? | ? | ? | ? | ? | ? | ? | ? |
| Aida Y, 2015(39) | Cross-sectional | 2008-2013 | One | Asian | ? | ? | ? | ? | 42±8.5 | 52±12.5 | 0.8 | 200±24 | 6.2±1 | 106.5±7.8 | ? | 197±41 |
| Alkhouri N, 2015(111) | ? | ? | One | ? | ? | ? | ? | ? | ? | ? | ? | ? | ? | ? | ? | ? |
| Anam M, 2017(118) | ? | ? | One | ? | 49.8 | 54.9 | ? | Yes | 28±5.5 | 58±12 | 0.48 | 238±21 | ? | ? | ? | ? |
| Angelidi A, 2017(88) | ? | ? | One | Caucasian | ? | 100 | ? | Yes | ? | ? | ? | ? | ? | ? | ? | ? |
| Angulo P, 2014(127) | Cross-sectional | 2003-2011 | Multiple | Caucasian 91.6 | 35.7 | 29.3 | 53.4 | Yes | 55±1 | 81±2 | 0.8±0.02 | 237±3 | ? | 114±2 | 194±4 | 209±2 |
| Angulo P, 2007(48) | Cross-sectional | 2000-2003 | Multiple | Caucasian | 30 | 30.5 | 60 | Yes | 60±50 | 87±72 | 0.94±0.9 | 235±84 | ? | 116±50 | 211±161 | 209±50 |
| Anstee Q, 2019(71) | Cross-sectional | ? | Multiple | Caucasian 67, Asian 26, Hispanic 27 | ? | 54.5 | ? | Yes | 37.5±9.7 | 42±10.5 | 0.79 | 227±38 | 6±1.8 | ? | ? | ? |
| Amernia B, 2021(2) | Cross-sectional | 2019 | One | ? | ? | ? | ? | ? | 44.7±22.5 | 51.5±43.3 | 0.92±0.25 | 236.6±91.8 | ? | ? | ? | ? |
| Arora S, 2016(113) | ? | ? | One | Caucasian 81 | 58 | 48 | 37 | Yes | ? | ? | ? | ? | ? | ? | ? | ? |
| Aykut U, 2014(34) | Cross-sectional | ? | One | Caucasian | ? | 19.3 | 24 | Yes | 53±31 | 84±56 | 0.63 | 224±194 | ? | 121±55 | ? | ? |
| Balakrishnan M, 2018(10) | Cross-sectional | 2015-2018 | One | Hispanic | ? | ? | ? | ? | ? | ? | ? | ? | ? | ? | ? | ? |
| Balakrishnan M, 2021(3) | Cross-sectional | 2010-2014 | One | Hispanic | ? | 53.5 | ? | ? | 68.8±57.6 | 102.6±80 | 0.75±0.44 | 235.9±72.5 | ? | ? | ? | ? |
| Barritt A, 2019(11) | Cross-sectional | ? | Multiple | Caucasian 87 | ? | 60 | ? | Yes | ? | ? | ? | ? | ? | ? | ? | ? |
| Boursier J, 2016(51) | Cross-sectional | 2003-2014 | Multiple | Caucasian | ? | 48 | ? | Yes | 48±30 | 69±49 | 0.69 | 217±70 | ? | ? | ? | ? |
| Boursier J, 2017(89) | ? | ? | One | Caucasian | ? | ? | ? | ? | ? | ? | ? | ? | ? | ? | ? | ? |
| Boursier J, 2019(90) | ? | 2004-2017 | Multiple | Caucasian | ? | 51.1 | ? | Yes | 39±6.2 | 56±11.3 | ? | 222±70 | ? | ? | ? | ? |
| Brandman D, 2017(124) | Longitunal | ? | Multiple | Caucasian 83 | ? | ? | ? | ? | ? | ? | ? | ? | ? | ? | ? | ? |
| Bril F, 2020(123) | Cross-sectional | ? | Multiple | Caucasian 58 | ? | 100 | ? | Yes | 41±26 | 57±390 | ? | ? | 7.1±1.1 | 148±42 | ? | ? |
| Broussier T, 2020(80) | Cross-sectional | 2014-2017 | One | Caucasian | ? | 38.1 | ? | Yes | 33±7.5 | 42±10 | 0.76 | 238±43 | ? | ? | ? | ? |
| Cales P, 2009(5) | Cross-sectional | 2001-2006 | Multiple | Caucasian | ? | ? | ? | ? | ? | ? | ? | ? | ? | ? | ? | ? |
| Cales P, 2010(8) | Cross-sectional | 2001-2006 | One | Caucasian | ? | ? | ? | ? | ? | ? | ? | ? | ? | ? | ? | ? |
| Cebreiros I, 2014(21) | Cross-sectional | 2012-2013 | One | Caucasian | 47.4 | 24.6 | 64.9 | Yes | 24.1±12.3 | 31.9±26 | 0.75 | 248±60.8 | ? | 87 | 141±55.5 | 175.1±41.3 |
| Cengiz M, 2015(40) | Cross-sectional | ? | One | Caucasian | ? | ? | ? | ? | 38.5±3.6 | 53±5.8 | 0.72 | 230±76.8 | ? | ? | ? | ? |
| Chan W, 2014(132) | Cross-sectional | 2012-2014 | One | Asian | 89.1 | 52.4 | 94.6 | Yes | 41±9.2 | 71±9.2 | 0.57 | ? | 6.5±1.5 | 108 | 153.2±62 | 192.5±420 |
| Chowdhury S, 2013(27) | Cross-sectional | 2008-2010 | One | Asian | ? | ? | ? | ? | 57±38 | 74±44 | ? | 153±65 | ? | ? | 146±94 | 153±50 |
| Cichoz-Lach H, 2012(22) | Cross-sectional | ? | One | Caucasian | ? | 23 | ? | Yes | 68±51 | 97±61 | 0.72±0.3 | 276±80 | ? | ? | ? | ? |
| Cui J, 2015(41) | Cross-sectional | 2012-2014 | One | Caucasian 52, Hispanic 28.4 | ? | 25.5 | ? | Yes | 42.3±35 | 58±56 | 0.82±0.31 | 246±62 | 6.1±0.9 | 108±33 | 153.9±75 | 181±38 |
| de Carli M, 2020(134) | Cross-sectional | 2013-2015 | One | Hispanic | 45.9 | 27.6 | ? | Yes | 33.8±17.4 | 50.1±33.7 | ? | 264.5±66 | 6.3±1.6 | ? | 164.5±79.9 | ? |
| de Cleva R, 2016(52) | Cross-sectional | 2005-2013 | One | Hispanic | 64.2 | 36.4 | 23.8 | Yes | 22.3±9 | 23.9±12 | ? | 271±71 | ? | ? | ? | ? |
| Demir M, 2011(24) | Cross-sectional | 1998-2009 | One | Caucasian | ? | ? | ? | ? | ? | ? | ? | ? | ? | ? | ? | ? |
| Demir M,2013(28) | Cross-sectional | 1998-2009 | One | Caucasian | 41 | 10 | 25 | Yes | 43.1±38 | 69±64 | 0.62 | 247±71 | ? | 103±30 | ? | ? |
| Dincses E, 2015(42) | Cross-sectional | ? | One | Caucasian | ? | 36.5 | ? | Yes | 61±38 | 89±58 | 0.7 | 229±91 | ? | 128±42 | 199±91 | 221±59 |
| Drolz A, 2017(35) | Cross-sectional | ? | One | Caucasian | 53 | 30 | ? | Yes | ? | ? | ? | ? | ? | ? | ? | ? |
| Dvorak K, 2014(36) | Cross-sectional | 2010-2013 | One | Caucasian | ? | ? | ? | ? | 77.1±42 | 135.5±96 | 0.65±0.2 | ? | ? | ? | 159.4 | ? |
| Eddowes P, 2019(114) | ? | ? | Multiple | ? | ? | ? | ? | ? | ? | ? | ? | ? | ? | ? | ? | ? |
| Fagan K, 2015(115) | Cohort | 1999-2013 | One | Caucasian 72.6 | 16.1 | 10.3 | 12.8 |  | ? | ? | ? | ? | ? | ? | ? | ? |
| Francque S, 2012(23) | Cross-sectional | 2005-2010 | One | Caucasian | ? | 8.4 | ? | ? | 31±14 | 43.3±22 | 0.75±0.18 | ? | 5.6±0.5 | 85.4±20 | 148±83 | 203±40 |
| Fujii H, 2009(6) | Cross-sectional | 1998-2007 | One | Asian | 46 | 44 | 62 | Yes | 72±74 | 106±86 | 0.67 | 192±85 | ? | 124±45 | 123±121 | 209±42 |
| Fujii H, 2009(135) | Cross-sectional | 2009 | One | Asian | 32.5 | 41 | 68 | Yes | ? | ? | ? | ? | ? | ? | ? | ? |
| Gallego-Duran R, 2012(91) | ? | ? | Multiple | Caucasian | ? | ? | ? | Yes | ? | ? | ? | ? | ? | ? | ? | ? |
| Guha I, 2008(59) | Cross-sectional | 2002-2006 | Multiple | Caucasian | ? | ? | ? | Yes | ? | 77.3 | ? | 239 | ? | 113 | 221.4 | ? |
| Guillaume M, 2019(130) | Cross-sectional | 2010-2017 | Multiple | Caucasian | 60.7 | 48.2 | 37.6 | Yes | 45±33 | 63±38 | 0.71 | 228±67 | ? | ? | ? | ? |
| Guturu P, 2008(119) | ? | 1998-2006 | One | Caucasian | ? | ? | ? | ? | ? | ? | ? | ? | ? | ? | ? | ? |
| Harrison S, 2008(70) | Cross-sectional | 2001-2005 | Multiple | Caucasian 68, Hispanic 24, African | 60 | 35 | 42 | Yes | 48 | 69 | 0.7 | ? | 5.9 | ? | ? | ? |
| Hagström H, 2019(87) | Retrospective cohort | 1971 - 2009 | Multiple | ? | 30 | 14 | 9 | ? | 40±20.7 | 73±46.6 | 0.58±0.20 | 243±62.9 | ? | 95±26 | 177±98.5 | 228±58 |
| Huang C, 2019(103) | ? | ? | ? | Asian | ? | ? | ? | ? | ? | ? | ? | ? | ? | ? | ? | ? |
| Inadomi C, 2020(136) | ? | 2016-2019 | Multiple | Asian | 64.5 | 50.3 | 66.5 | Yes | 46±23 | 59.5±32.5 | 0.77 | 204 ±81 | 6.3±1.6 | 113±32 | 131±101 | 188±61 |
| Isgro M, 2014(50) | Cross-sectional | ? | One | Caucasian | ? | ? | ? | ? | ? | ? | ? | ? | ? | ? | ? | ? |
| Itoh Y, 2018(66) | Cross-sectional | 2013-2015 | One | Asian | 42 | 38 | 47 | Yes | 46 | 63 | 0.73 | 206 | ? | ? | ? | ? |
| Joo S, 2017(104) | ? | ? | One | Asian | ? | ? | ? | ? | ? | ? | ? | ? | ? | ? | ? | ? |
| Joo, S 2015(92) | ? | 2013-2014 | One | Caucasian | ? | ? | ? | ? | ? | ? | ? | ? | ? | ? | ? | ? |
| Jouness R, 2016(93) | ? | ? | Multiple | Caucasian | ? | ? | ? | ? | ? | ? | ? | ? | ? | ? | ? | ? |
| Kao W, 2020(105) | Prospective cohort | 2016-2018 | One | Asian | ? | 26.8 | 16.9 | Yes | 38.2 ± 32.2 | 55.0 ± 42.2 | 0.69 | 292.0±71.4 | ? | 119.4 ± 57.8 | 161.9 ± 112.5 | 190.0 ± 37.6 |
| Kawamura, 2015(43) | Cross-sectional | 1990-2011 | One | Asian | ? | 27.7 | ? | ? | 65.7±61 | 108.1±65 | ? | 196.3±86 | ? | ? | 131.8±42 | 200.8 |
| Kim D, 2011(106) | ? | 2002-2009 | One | Asian | ? | ? | ? | ? | ? | ? | ? | ? | ? | ? | ? | ? |
| Kim D, 2013(137) | Cross-sectional | 2007-2010 | One | ? | 45.1 | 27.5 | ? | Yes | 47.2±31 | 60.4±55 | 0.78 | 216±79 | ? | ? | 143.4±77 | 172.3±40 |
| Kobayashi N, 2017(58) | Cross-sectional | 1999-2013 | One | Asian | 45 | 46.4 | 51.4 | Yes | 60 | 85 | 0.7 | 235 | ? | ? | 152.6 | 203.3 |
| Kolhe K, 2019(72) | Cross-sectional | 2012-2013 | One | Asian | ? | ? | ? | ? | 46.1±28 | 52.1±24.1 | 0.88 | 220±75 | ? | ? | ? | ? |
| Kosick H, 2019(116) | ? | 2010-2018 | One | Caucasian | ? | ? | ? | ? | ? | ? | ? | ? | ? | ? | ? | ? |
| Kruger F, 2008(120) | ? | ? | One | Caucasian | ? | ? | ? | ? | ? | ? | ? | ? | ? | ? | ? | ? |
| Kruger F, 2011(125) | ? |  | Multiple | African 69% | ? | ? | ? | ? | ? | ? | ? | ? | ? | ? | ? | ? |
| Kumar R, 2013(33) | Cross-sectional | 2009-2011 | One | Asian | 15.8 | 16.6 | ? | Yes | 45±93 | 62.5±74 | 0.72 | 186±111 | ? | 99.1 | 165.6±77 | 180±48 |
| Labenz C, 2018(67) | Cross-sectional | ? | Multiple | Caucasian | 54.4 | 29.9 | 37.5 | Yes | 48±55 | 60±75 | ? | 234±104 | 7.1 | ? | ? | ? |
| Lambrecht J, 2019(94) | ? | ? | One | Caucasian | ? | ? | ? | ? | 37±10.7 | 45.5±9 | 0.79±0.27 | ? | ? | ? | ? | ? |
| Lang S, 2020(83) | Cross-sectional | 2015-2018 | One | Caucasian | 74.6 | 39 | ? | Yes | 46.5±23 | 47.8±38.5 | ? | 193±81 | 5.7 | 111.5±35.6 | 154±125 | 176±51.7 |
| Lardi L, 2020(139) | ? | ? | One | Hispanic | ? | ? | ? | ? | ? | ? | ? | ? | ? | ? | ? | ? |
| Lassailly G, 2011(17) | Cross-sectional | 2006-2009 | One | Caucasian | 60.4 | 31.9 | 58 | Yes | ? | 30.8 | ? | ? | ? | ? | ? | ? |
| Le P, 2018(128) | ? | 2004-2008 | Multiple | Caucasian 80 | ? | ? | ? | ? | ? | ? | ? | ? | ? | ? | ? | ? |
| Lee T, 2013(29) | Cross-sectional | 2002-2006 | One | ? | 49 | 32.7 | 28.9 | Yes | 55±11.2 | 63±19 | 0.90±0.62 | ? | ? | ? | 157±35 | 183.5±21 |
| Liu W, 2020(84) | Cross-sectional | 2017-2019 | One | Asian | 35 | 31.5 | ? | ? | 47.9±31.8 | 80.5±76.4 | 0.59 | 242.1 ± 58.8 | 6.1 ± 1.2 | 100±27 | 201±131 | 194±73 |
| Loaeza-del-Castillo, 2008(81) | Cross-sectional | ? | One | Hispanic | ? | ? | ? | ? | 72 ±171 | ? | ? | 262 ±65 | ? | ? | 150 | ? |
| Loong T, 2017(60) | Cross-sectional | 2006-2013 | One | Asian | 49.8 | 54.9 | ? | Yes | 28±5.5 | 58±12 | 0.48 | 238±21 | ? | ? | ? | ? |
| Luger M, 2016(95) | Randomized controlled trial | 2014-2015 | One | Caucasian | 52 | 26 | 48 | Yes | 28±13 | 36.4±20 | ? | 281±64 | 6±1.3 | 108±36.6 | 198±46 | ? |
| Mahadeva S, 2013(30) | Cross-sectional | 2009-2010 | One | Asian | 48.1 | 47.3 | 60.3 | Yes | ? | ? | ? | ? | ? | ? | ? | ? |
| Marella H, 2019(129) | Cross-sectional | 2006-2016 | One | Caucasian, Africanamerican | ? | 34.1 | ? | Yes | 40±37 | 52±51 | ? | 266±88 | 6.4±1.9 | ? | 184.4±202.3 | 181.1±52.8 |
| McPherson S, 2010(9) | Cross-sectional | 2003-2009 | One | Caucasian | ? | 50.3 | ? | Yes | 63±44 | 94±63 | ? | 255±91 | ? | ? | 239.1±194 | 208.8±54 |
| McPherson S, 2017(61) | Cross-sectional | ? | Multiple | Caucasian | ? | 45.6 | ? | Yes | 49.8±4.5 | 73.4±9.8 | 0.67 | 246 | ? | ? | ? | ? |
| McPherson, 2013(31) | Cross-sectional | 1999-2009 | One | Caucasian | ? | 43 | ? | Yes | 43.5 | 61.5 | 0.7 | 244 | ? | ? | 203.5 | 208.4 |
| Meneses D, 2020(82) | Cross-sectional | ? | One | Caucasian | 52 | 26 | 28 | Yes | 21±3 | 25±4.25 | 0.84 | 274±65 | 5.7 | 101±5 | 140±25 | 183.7±42 |
| Miao C, 2010(117) | ? | ? | One | Caucasian | ? | ? | ? | ? | ? | ? | ? | ? | ? | ? | ? | ? |
| Miele L, 2015(96) | ? | ? | One | Caucasian | ? | ? | ? | ? | ? | ? | ? | ? | ? | ? | ? | ? |
| Miele L, 2017(62) | Prospective | ? | One | Caucasian | 34.2 | 15.8 | ? | Yes | 38±19.5 | 57±27.7 | 0.66 | 222±61 | ? | 91±26.5 | 126±67.5 | 201±43.5 |
| Miller A, 2019(57) | Cross-sectional | ? | One | Caucasian 87 | ? | ? | ? | ? | ? | ? | ? | ? | ? | ? | ? | ? |
| Miller M, 2010(15) | Cross-sectional | ? | One | Caucasian | ? | ? | ? | ? | ? | ? | ? | ? | ? | ? | ? | ? |
| Munteanu M, 2016(97) | Cohort | 2005-2014 | Multiple | Caucasian | ? | 22.7 | ? | Yes | ? | ? | ? | ? | ? | ? | ? | ? |
| Nascimbeni, 2015(98) | Cohort | ? | Multiple | Caucasian | ? | ? | ? | ? | ? | ? | ? | ? | ? | ? | ? | ? |
| Nassif AT, 2017(63) | Cross-sectional | 2012-2013 | One | Hispanic | ? | ? | ? | ? | ? | ? | ? | ? | ? | ? | ? | ? |
| Okajima A, 2017(64) | Cross-sectional | 2013-2015 | One | Asian | 36.8 | ? | 57.1 | Yes | 53±34 | 73±52 | 0.72 | 222±66 | 6.4±1.1 | ? | 180±132 | ? |
| Pastor-Ramirez, 2017(99) | ? | ? | Multiple | Caucasian | ? | 30 | ? | Yes | ? | ? | ? | ? | ? | ? | ? | ? |
| Pathik P, 2015(44) | Cross-sectional | 2011-2012 | One | Asian | 16.9 | 49 | ? | Yes | ? | ? | ? | 243±8.2 | ? | ? | 220±11 | 223±7 |
| Peleg N, 2017(65) | Cross-sectional | 2005-2012 | One | Caucasian | ? | 34.6 | ? | Yes | 59.7 | 69.8 | 0.85 | 188.8 | 6.59 | ? | 150 | 171 |
| Pérez-Gutiérrez O, 2013(32) | Cross-sectional | 2005-2011 | Multiple | Hispanic | ? | 21.5 | ? | Yes | 57.6±58 | 73±82 | ? | 238±95 | ? | 107±25 | ? | ? |
| Petta S, 2015(45) | Cross-sectional | ? | One | Caucasian | 24 | 19.5 | ? | ? | 43.9±28 | 78±49 | 0.56 | 225±65 | ? | 98.5±26 | 151±93 | 205±46 |
| Petta S, 2017(138) | Cross-sectional | 2009 | Multiple | ? | 43.4 | 54.7 | ? | ? | 47.4±40.2 | 77.3±58.8 | 0.61 | 229±70.3 | ? | 111±41.3 | 161.2±102.4 | 201.8±46.9 |
| Pimentel S, 2010(12) | Cross-sectional | ? | One | Hispanic | ? | 16 | ? | Yes | 30±17 | 38±29 | ? | 279±73 | ? | ? | ? | ? |
| Polyzos S, 2019(73) | Cross-sectional | 2008-2010 | One | Caucasian | ? | ? | ? | ? | 38.3±5.6 | 56.2±10.5 | 0.68 | 226±16.5 | ? | 104±6 | 180.5±24 | ? |
| Prasad S, 2020(107) | ? | ? | One | Asian | ? | ? | ? | ? | ? | ? | ? | ? | ? | ? | ? | ? |
| Qureshi K, 2008(86) | Cross-sectional | 2002-2007 | One | Caucasian 86 | 67 | 35 | 50 | Yes | 25±11 | 29±16 | 0.86 | 305 | ? | 115±38 | 156±92 | 198±39 |
| Raszeja-Wyszomirska J, 2010(13) | Cross-sectional | 2006-2009 | One | Caucasian | 29.7 | 17.8 | 68.9 | Yes | 51.5±34 | 80.8±55 | 0.79 | 223±62 | 6.2±1 | 102.4±23 | ? | ? |
| Rath M, 2016(53) | Cross-sectional | 2011-2013 | One | Asian | 26.6 | 16.7 | 62 | Yes | 41.5±18 | 60±43.5 | ? | 215±42 | ? | ? | 170±113 | ? |
| Ratziu C, 2006(26) | Cross-sectional | 2001-2004 | Multiple | Caucasian | 23.5 | 34 | 39 | Yes | 47.5 | 75 | 0.63 | ? | ? | ? | ? | ? |
| Ratziu V, 2004(1) | ? | ? | One | Caucasian | ? | ? | ? | ? | ? | ? | ? | ? | ? | ? | ? | ? |
| Ruffillo G, 2011(18) | Cross-sectional | ? | One | Hispanic | ? | 23.1 | 65.9 | Yes | ? | 69 | 0.57 | 235 | ? | ? | ? | ? |
| Saez E, 2017(100) | ? | ? | One | Caucasian | ? | ? | ? | ? | ? | ? | ? | ? | ? | ? | ? | ? |
| Sebastiani G, 2011(19) | Cross-sectional | 2003-2008 | Multiple | Caucasian | ? | ? | ? | ? | 79.6±58 | 72.3±47 | ? | 251±81 | ? | ? | ? | ? |
| Seth A, 2016(68) | Cross-sectional | 2010-2014 | One | Hispanic 77 | ? | 50 | ? | ? | ? | ? | ? | ? | 7.5 | ? | ? | ? |
| Shah A, 2009(7) | Cross-sectional | ? | One | Caucasian 74 | 44 | 19 | ? | Yes | 43 | 63 | 0.68 | 241 | 14 | ? | 148 | 120 |
| Shaheen A, 2016(121) | Cohort | ? | One | Caucasian | ? | ? | ? | ? | ? | ? | ? | ? | ? | ? | ? | ? |
| Shima T, 2019(74) | Cross-sectional | 2013-2016 | One | Asian | 60.4 | 58.6 | 76.6 | Yes | 56.4±35.4 | 81.7±56.8 | 0.69 | 210±64 | ? | 113.3 ± 39.1 | ? | 200.5 ± 38.8 |
| Shoji H, 2016(54) | Cross-sectional | ? | Multiple | Asian | ? | ? | ? | ? | 48±57 | 66±12 | ? | 198±83 | ? | ? | ? | 193±20 |
| Shukla A, 2015(46) | Cross-sectional | ? | One | Asian | 17.6 | 19.6 | ? | ? | 59.3±46 | 51.5±38 | ? | 216±70 | ? | ? | 154.2±70 | 175±51 |
| Siddiqui M, 2016(55) | Cross-sectional | 2006-2013 | One | Caucasian 86 | 58 | 39.3 | 49.3 | Yes | 62.3±43 | 80.7±53 | 0.77 | 254±96 | ? | 122±53 | ? | ? |
| Siddiqui M, 2019(75) | Cross-sectional | 2004-2009 | Multiple | Caucasian 73, Hispanic 13 | 58 | 39 | 62 | Yes | 51.2±36.7 | 69.8±50.6 | 0.73 | 236±69.8 | ? | 110.6±38.4 | ? | ? |
| Simo K, 2014(38) | Cross-sectional | 2005-2011 | Multiple | Caucasian 67.1 | ? | 29.3 | ? | Yes | 27.8±17.3 | 31.2±24.3 | 0.98±0.28 | 298.9±66.8 | ? | 111.2±46.9 | 163.3±97.5 | 196.5±41.7 |
| Singh A. 2020(85) | Cross-sectional | 2000-2015 | One | Caucasian | 75.9 | 100 | 70.8 | Yes | 27±5.5 | 28±18-45 | 0.96 | 217.6±83 | 6.7±1.5 | 110±12 | 124±24 | 167.8±43.6 |
| Singh T, 2018(126) | Cohort | 2006-2015 | Multiple | ? | ? | ? | ? | ? | ? | ? | ? | ? | ? | ? | ? | ? |
| Sjowall C, 2015(47) | Cross-sectional | ? | One | Caucasian | ? | 51 | ? | ? | 39±16 | 66.2±40 | 0.59 | 231±66 | ? | ? | ? | ? |
| Stauber R, 2018(101) | Cohort | ? | One | Caucasian | ? | ? | ? | ? | ? | ? | ? | ? | ? | ? | ? | ? |
| Staufer K, 2019(76) | Cross-sectional | 2011-2016 | Multiple | Caucasian | ? | 30 | ? | Yes | 41±7.2 | 55±12 | 0.74 | 223±89 | ? | ? | ? | ? |
| Subasi C, 2015(49) | Cross-sectional | ? | One | Caucasian | ? | 16.9 | ? | Yes | 58±36 | 91±61 | 0.63 | 226±84 | ? | 124±47 | 194±95 | 211±53 |
| Sumida Y, 2012(20) | Cross-sectional | 2002-2008 | Multiple | Asian | 32 | 42 | ? | Yes | 43±9.5 | 69±17 | 0.62±0.7 | 227±67 | ? | ? | 147±25 | 209±40 |
| Takeuchi H, 2018(69) | Cross-sectional | 2010-2015 | One | Asian | ? | ? | ? | ? | 59.5±52.70 | 87.9±90.3 | ? | 223±82.4 | 7.1±1.8 | 127.4±51.2 | 155±81 | 185.5±39.3 |
| Tanwar S, 2006(102) | ? | ? | Multiple | Caucasian | ? | ? | ? | ? | ? | ? | ? | ? | ? | ? | ? | ? |
| Thanapirom K, 2017(122) | ? | 2016 | One | ? | ? | ? | ? | ? | 32.9±23.2 | 50.4±45.2 | ? | 272±62 | ? | ? | ? | ? |
| Tomeno W, 2019(77) | Cross-sectional | 2014-2017 | One | Asian | 47.1 | 31.1 | 56.1 | Yes | 29±20.3 | 30±30.3 | 0.94±0.33 | 188±39 | ? | ? | ? | ? |
| Treeprasertsuk S, 2016(56) | Cross-sectional | 2009-2012 | One | Asian | ? | 38 | ? | Yes | 38±39 | 56±47 | 0.67 | 267±64 | 7.3±8.7 | 112±33 | 146±60 | 198±46 |
| Uy D, 2011(108) | ? | 2007-2010 | One | Asian | ? | 16.4 | ? | Yes | ? | ? | ? | ? | ? | ? | ? | ? |
| Wong V, 2010(14) | Cross-sectional | 2003-2009 | Multiple | Caucasian 52, Chinese 48 | 40.2 | 36.2 | ? | Yes | ? | 75±54 | ? | ? | ? | 115±46 | 85.07±123 | 208.8±50.2 |
| Xun Y, 2012(25) | Cross-sectional | 2005-2010 | One | Asian | ? | 32.2 | ? | Yes | 61±41 | 100±74 | 0.61 | 200±56 | ? | ? | ? | ? |
| Yang M, 2019(78) | Cross-sectional | 2012-2017 | Multiple | Asian | 34.8 | 30.2 | ? | Yes | 74.1±35.6 | 135.1±48.9 | 0.69±0.37 | 240.1±66.5 | ? | 96.5±24.1 | 188.1±81.3 | 191.8±80 |
| Yoneda M, 2013(109) | Cross-sectional | 2002-2011 | One | Asian | ? | 46 | 63.8 | Yes | 24.7±10 | 23.7±7 | 1 | 211±69 | 5.9±0.9 | ? | 140±72 | 202.6 |
| Younes R, 2021(4) | Multicenter Cohort | 1995 - 2015 | Multiple | Caucasian | ? | 28.2 | ? | ? | 37±26 | 59±34.8 | ? | 225±83 | ? | ? | ? | ? |
| Zhou Y, 2019(110) | ? | 2016-2018 | One | Asian | 42 | 51 | 96 | Yes | 45.7±32.3 | 49±20.8 | 0.8±0.4 | 249.9±58 | ? | 110±30 | 442±106 | 92.6±58 |
| Zou C, 2019(133) | ? | ? | One | Asian | ? | ? | ? | ? | ? | ? | ? | ? | ? | ? | ? | ? |

*ALT Alanine aminotransferase AST; Aspartate aminotransferase; DM Diabetes Mellitus; DLD Dyslipidemia; HTN Hypertension; HbA1c glycated hemoglobin; MetS Metabolic syndrome, SD Standards Deviation, ? not responded*

**Supplementary Table 3.** Characteristics of the studies included in the systematic review according to the serological biomarkers in predicting liver fibrosis severity in people with MASLD

|  | **Number studies from severity** | | | | | | |
| --- | --- | --- | --- | --- | --- | --- | --- |
| **Score Models** | | **Number of participants** | **Any fibrosis** | **Significant fibrosis** | **Advanced fibrosis** | **Cirrhosis** | **Total** |
| **APRI** | | 29 136 | 3 | 34 | 40 | 3 | 80 |
| **FIB-4** | | 36 357 | 5 | 37 | 43 | 4 | 89 |
| **NFS** | | 31 538 | 5 | 36 | 43 | 3 | 87 |
| **BARD score** | | 18 869 | 1 | 20 | 29 | 2 | 52 |
| **FibroMeter** | | 4 206 | 2 | 4 | 12 | 1 | 17 |
| **FibroTest** | | 2 079 | 0 | 4 | 6 | 2 | 11 |
| **ELF** | | 2 403 | 2 | 2 | 6 | 0 | 14 |
| **Forns score** | | 1 045 | 0 | 1 | 1 | 1 | 3 |
| **Hepa score** | | 2 122 | 0 | 2 | 2 | 0 | 4 |

*APRI aspartate aminotransferase to platelet ratio index; ELF enhanced liver fibrosis; FIB-4 fibrosis index-4; NFS Non-alcoholic fatty liver disease fibrosis score.*

**Supplementary Table 4.** Cut-off values of serological biomarkers from the studies included in predicting liver fibrosis severity in people with MASLD

| **First author (year of publication) ^ref^** | **FIB-4** | **APRI** | **NFS** | **BARD Score** | **FibroMeter** | **FibroTest** | **ELF** | **Hepascore** | **Forns score** |
| --- | --- | --- | --- | --- | --- | --- | --- | --- | --- |
| Abe M, 2014^20^ | ? | ? | ? | NA | NA | NA | NA | NA | NA |
| Adams L, 2008^21^ | NA | ? | NA | NA | NA | ? | NA | ? | NA |
| Adams L, 2011^57^ | SF: 1.45  AF: 1.54  Cirrhosis: 1.92 | SF: 0.43  AF: 0.54  Cirrhosis: 1.92 | NA | SF: 2.00  AF: 2.00  Cirrhosis: 3.00 | SF: 0.34  AF: 0.47  Cirrhosis: 0.57 | SF: 0.34  AF: 0.47  Cirrhosis: 0.57 | NA | SF: 0.44  AF: 0.37  Cirrhosis: 0.70 | NA |
| Ahmed Z, 2016^35^ | ? | ? | NA | NA | NA | NA | NA | NA | NA |
| Aida Y, 2015^31^ | AF: 2.09 | AF: 0.67 | NA | NA | NA | NA | NA | NA | NA |
| Alkhouri N, 2015^95^ | ? | ? | ? | NA | NA | NA | NA | NA | NA |
| Anam M, 2017^38^ | ? | ? | ? | ? | ? | NA | NA | NA | NA |
| Angelidi A, 2017^66^ | SF: 1.00 | SF: >0.23 | SF: >-1.44 | SF: 2.50 | NA | NA | NA | NA | NA |
| Angulo P, 2014^96^ | ? | ? | AF: >0.67 | ? | NA | NA | NA | NA | NA |
| Angulo P, 2007^97^ | NA | NA | AF: 0.67 | NA | NA | NA | NA | NA | NA |
| Anstee Q, 2019^98^ | AF: 2.67 | NA | AF: 0.68 | NA | NA | NA | AF: 11.30 | NA | NA |
| Amernia B, 2021^99^ | AF: 1.19 | AF: 0.70 | NA | NA | NA | NA | NA | NA | NA |
| Arora S, 2016^62^ | AF: 1.10 | ? | ? | ? | NA | NA | NA | NA | NA |
| Aykut U, 2014^30^ | NA | NA | ? | NA | AF: 0.82 | NA | NA | NA | NA |
| Balakrishnan M, 2018^100^ | AF: 1.45 | AF: 1.00 | SF: 1.43  AF: 1.43 | AF: 2.00 | NA | NA | NA | NA | NA |
| Balakrishnan M, 2021^101^ | AF: 1.30 | AF: 1.00 | AF: 0.67  AF: -1.45 | AF: 2.00 | NA | NA | NA | NA | NA |
| Barritt A, 2019^74^ | NA | AF: 1.30 | AF: 0.67 | NA | NA | NA | NA | NA | NA |
| Boursier J, 2016^63^ | AF: 1.52 | AF: 0.56 | AF: 1.04 | AF: 2.00 | AF: 0.31 | AF: 0.32 | NA | AF: 0.32 | NA |
| Boursier J, 2017^39^ | NA | NA | ? | NA | ? | NA | NA | NA | NA |
| Boursier J, 2019^44^ | AF: <1.30 | NA | AF: <-1.45 | NA | AF: <0.46 | ? | NA | ? | NA |
| Brandman D, 2017^40^ | Cirrhosis: 1.67 | Cirrhosis: 0.54 | Cirrhosis: 0.28 | Cirrhosis: 3.00 | NA | NA | NA | NA | NA |
| Bril F, 2020^102^ | NA | NA | NA | NA | NA | AF: 0.35 | NA | NA | NA |
| Broussier T, 2020^103^ | AF: <1.30 | NA | NA | NA | AF: <0.46 | NA | NA | NA | NA |
| Cales P, 2009^104^ | NA | ? | ? | NA | SF: 0.29 | NA | NA | NA | NA |
| Cales P, 2010^105^ | NA | NA | ? | NA | NA | ? | NA | NA | NA |
| Cebreiros I, 2014^106^ | NA | NA | NA | NA | AnF: 2.75 | NA | AF: 8.72 | NA | NA |
| Cengiz M, 2015^107^ | ? | ? | NA | NA | NA | NA | NA | NA | NA |
| Chan W, 2014^108^ | NA | NA | AF: >-1.45 | NA | NA | NA | NA | NA | NA |
| Chowdhury S, 2013^59^ | NA | AF: 1.00 | NA | NA | NA | NA | NA | NA | NA |
| Cichoz-Lach H, 2012^109^ | NA | NA | AF: >0.67 | AF: ≥0.8 | NA | NA | NA | NA | NA |
| Cui J, 2015^110^ | AF: 1.30 | ? | ? | ? | NA | NA | NA | NA | NA |
| de Carli M, 2020^75^ | AF: 2.67 | AF: >0.98 | AF: >0.67 | AF: ≥ 2 | NA | NA | NA | NA | NA |
| de Cleva R, 2016^111^ | NA | AF: 0.44 | NA | NA | NA | NA | NA | NA | NA |
| Demir M, 2011^24^ | NA | NA | AF: 0.67 | ? | NA | NA | NA | NA | NA |
| Demir M,2013^112^ | SF: 1.45  AF: 3.25 | NA | SF: 1.46  AF: 0.67 | SF: 0-1  AF: 2-4 | NA | NA | NA | NA | NA |
| Dincses E, 2015^113^ | NA | NA | ? | NA | ? | NA | NA | NA | NA |
| Drolz A, 2017^114^ | ? | ? | ? | ? | NA | NA | NA | NA | NA |
| Dvorak K, 2014^115^ | SF: 1.24 | SF: 0.65 | SF: 2.16 | AF: 0.71 | NA | NA | ? | NA | NA |
| Eddowes P, 2019^45^ | AF: 3.25 | NA | AF: 0.67 | NA | AF: 0.71 | NA | NA | NA | NA |
| Fagan K, 2015^60^ | NA | NA | NA | NA | NA | NA | AF: ≥9.8 | NA | NA |
| Francque S, 2012^116^ | ? | ? | AF: >2.14 | ? | NA | NA | NA | NA | ? |
| Fujii H, 2009^47^ | NA | AF: >2.00 | NA | NA | NA | NA | NA | NA | NA |
| Fujii H, 2009^117^ | NA | NA | NA | ? | NA | NA | NA | NA | NA |
| Gallego-Duran R, 2012^27^ | NA | NA | ? | NA | NA | AF: <0.58 | NA | NA | NA |
| Guha I, 2008^89^ | NA | NA | NA | NA | NA | NA | AF: -1.24 | NA | NA |
| Guillaume M, 2019^118^ | NA | NA | NA | NA | AF: 0.43 | NA | AF: 9.3 | NA | NA |
| Guturu P, 2008^119^ | NA | SF: <0.44 | NA | ? | NA | NA | NA | NA | NA |
| Harrison S, 2008^120^ | NA | NA | NA | AF: >2.00 | NA | NA | NA | NA | NA |
| Hagström H, 2019^80^ | ? | ? | AF: 1.00 | AF: 1.45 | NA | NA | NA | NA | NA |
| Huang C, 2019^121^ | ? | ? | ? | ? | ? | NA | NA | NA | NA |
| Inadomi C, 2020^122^ | SF: 2.67  AF: 2.67 | NA | NA | NA | NA | NA | SF: 9.86  AF: 10.36 | NA | NA |
| Isgro M, 2014^123^ | NA | NA | NA | NA | NA | NA | ? | NA | NA |
| Itoh Y, 2018^124^ | NA | NA | NA | NA | NA | NA | AF: 9.2 | NA | NA |
| Joo S, 2017^125^ | ? | NA | ? | ? | NA | NA | NA | NA | NA |
| Joo, S 2015^32^ | ? | NA | NA | NA | NA | NA | NA | NA | NA |
| Jouness R, 2016^36^ | ? | NA | ? | NA | NA | NA | NA | NA | NA |
| Kao W, 2020^81^ | SF: 0.66 | AF: 0.40 | SF: 1.90 | NA | NA | NA |  | NA | NA |
| Kawamura, 2015^126^ |  |  | NA | NA | NA | NA | NA | NA | NA |
| Kim D, 2011^49^ | ? | ? | ? | ? | NA | NA | NA | NA | NA |
| Kim D, 2013^127^ | AF: 3.25 | AF: 1.50 | AF: 0.68 | AF: 2.00 | NA | NA | NA | NA | NA |
| Kobayashi N, 2017^128^ | SF: 0.85 | AF: 0.45 | NA | NA | NA | NA | NA | NA | NA |
| Kolhe K, 2019^76^ | SF: 1.45 | SF: 0.45 | NA | NA | NA | NA | NA | NA | NA |
| Kosick H, 2019^77^ | ? | ? | ? | ? | NA | NA | NA | NA | NA |
| Kruger F, 2008^129^ | NA | AF: 0.98 | AF: -1.30 | NA | NA | NA | NA | NA | NA |
| Kruger F, 2011^130^ | NA |  | AF:1.31 | NA | NA | NA | NA | NA | NA |
| Kumar R, 2013^28^ | ? | ? | ? | ? | NA | NA | NA | NA | NA |
| Labenz C, 2018^131^ | ? | ? | ? | NA | NA | NA | NA | NA | NA |
| Lambrecht J, 2019^46^ | SF: 1.50 | SF: 0.49 | NA | NA | NA | NA | NA | NA | NA |
| Lang S, 2020^83^ | AnF: 1.3 | NA | AnF: 0.68 | NA | NA | NA | NA | NA | NA |
| Lardi L, 2020^132^ | NA | NA | NA | NA | NA | ? | NA | NA | NA |
| Lassailly G, 2011^25^ | NA | NA | NA | NA | NA | ? | NA | NA | NA |
| Le P, 2018^42^ | AF: 1.64 | AF: 0.59 | NA | ? | NA | NA | NA | NA | NA |
| Lee T, 2013^51^ | ? | NA | ? | ? | ? | NA | NA | NA | NA |
| Liu W, 2020^84^ | SF: 0.87 | NA | NA | SF: 1.00 | NA | NA | NA | NA | NA |
| Loaeza-del-Castillo, 2008^133^ | NA | SF: <0.2  Cirrhosis: <0.5 | NA | NA | NA | NA | NA | NA | NA |
| Loong T, 2017^67^ | NA | NA | NA | NA | AF: 0.82 | NA | NA | NA | NA |
| Luger M, 2016^37^ | ? | NA | ? | NA | NA | NA | NA | NA | NA |
| Mahadeva S, 2013^134^ | NA | ? | ? | NA | NA | NA | NA | NA | NA |
| Marella H, 2019^86^ | AF: 2.67 | AF: 1.50  Cirrhosis: 1.50 | AF:0.67  Cirrhosis: 0.67 | AF: 2.00 | NA | NA | NA | NA | NA |
| McPherson S, 2010^135^ | AF: 1.30 | AF: 1.00 | AF: 1.45 | ? | NA | NA | NA | NA | NA |
| McPherson S, 2017^68^ | AF: 2.67 | ? | AF: 0.68 | NA | NA | NA | NA | NA | NA |
| McPherson, 2013^93^ | AF: 3.25 | NA | AF: 1.45 | AF: 2.00 | NA | NA | NA | NA | NA |
| Meneses D, 2020^82^ | SF: 2.67 | SF: 0.98 | SF: 0.68 | SF: 3.00 | NA | NA | NA | NA | ? |
| Miao C, 2010^136^ | ? | NA | ? | ? | NA | NA | NA | NA | NA |
| Miele L, 2015^137^ | NA | NA | NA | NA | NA | NA | AF: 9.80 | NA | NA |
| Miele L, 2017^138^ | NA | NA | NA | NA | NA | NA |  | NA | NA |
| Miller A, 2019^139^ | AF: >3.25 | AF: >2.00 | AF: 0.67 | NA | NA | NA | NA | NA | NA |
| Miller M, 2010^23^ | ? | NA | ? | ? | NA | NA | NA | NA | NA |
| Munteanu M, 2016^64^ | ? | NA | ? | ? | NA | Cirrhosis: 0.74 | NA | NA | NA |
| Nascimbeni, 2015^33^ | ? | ? | ? | ? | NA | NA | NA | NA | NA |
| Nassif AT, 2017^69^ | NA | NA | NA | AF: >2.00 | NA | NA | NA | NA | NA |
| Okajima A, 2017^70^ | AnF: 1.44 | AnF: 0.49  SF: 0.69  AF: 0.78 | AnF: 1.85  SF: 1.94  AF: 0.11 | NA | NA | NA | NA | NA | NA |
| Pastor-Ramirez, 2017^140^ | ? | ? | ? | ? | NA | NA | NA | NA | NA |
| Pathik P, 2015^34^ | NA | AF: 0.68 | AF: 1.00 | NA | NA | NA | NA | NA | NA |
| Peleg N, 2017^141^ | AF: 1.30 | AF: 1.00 | NA | NA | NA | NA | NA | NA | NA |
| Pérez-Gutiérrez O, 2013^142^ | AF: ≥3.25 | AF: ≥1.00 | AF: ≥0.67 | AF: ≥2.00 | NA | NA | NA | NA | NA |
| Petta S, 2015^143^ | AF: >2.67 | NA | AF: >0.67 | NA | NA | NA | NA | NA | NA |
| Petta S, 2017^71^ | AF: 2.67 | NA | AF: 0.68 | NA | NA | NA | NA | NA | NA |
| Pimentel S, 2010^55^ | NA | NA | AF: >0.67 | NA | NA | NA | NA | NA | NA |
| Polyzos S, 2019^144^ | ? | SF: 0.50 | ? | NA | NA | NA | SF: 9.00 | NA | NA |
| Prasad S, 2020^85^ | AF: 2.02 | AF: 1.09 | AF: 0.24 | NA | NA | NA | NA | NA | NA |
| Qureshi K, 2008^22^ | NA | NA | SF: 0.67  AF: 0.67 | NA | NA | NA | NA | NA | NA |
| Raszeja-Wyszomirska J, 2010^48^ | NA | NA | NA | SF: 2.00 | NA | NA | NA | NA | NA |
| Rath M, 2016^145^ | NA | AF: 0.56 | AF: 1.46 | ? | NA | NA | NA | NA | NA |
| Ratziu C, 2006^146^ | NA | NA | NA | NA | NA | AF: 0.70 | NA | NA | NA |
| Ratziu V, 2004^53^ | NA | NA | NA | NA | NA | SF: 0.30 | NA | NA | NA |
| Ruffillo G, 2011^147^ | NA | NA | AF: 0.67 | AF: >2.00 | NA | NA | NA | NA | NA |
| Saez E, 2017^41^ | NA | SF: 0.45 | SF: 0.36 | ? | NA | NA | NA | NA | NA |
| Sebastiani G, 2011^148^ | NA | SF: 1.50 | NA | NA | NA | SF: 0.48 | NA | NA | NA |
| Seth A, 2016^149^ | ? | ? | ? | ? | NA | NA | NA | NA | NA |
| Shah A, 2009^150^ | AF: 2.67 | NA | NA | NA | NA | NA | NA | NA | NA |
| Shaheen A, 2016^65^ | AnF: 1.87 | ? | ? | NA | NA | NA | NA | NA | NA |
| Shima T, 2019^78^ | AF: 1.87 | AnF: 0.39  AF: 0.55 | AnF: 1.54  AF: 0.58 | NA | NA | NA |  | NA | NA |
| Shoji H, 2016^151^ | SF: 1.37  AF: 2.25 | SF: 0.75  AF: 0.88 | SF: 1.10  AF: 0.53 | ? | NA | NA | NA | NA | NA |
| Shukla A, 2015^152^ | AF: 2.64 | NA | NA | NA | NA | NA | NA | NA | NA |
| Siddiqui M, 2016^153^ | SF: 1.31  AF: 1.96 | AF: 0.71 | AF: 0.16 | AnF: 2.00  AF: 4.00 | AnF: 0.15 | NA | NA | NA | NA |
| Siddiqui M, 2019^90^ | AF: 1.37 | AnF: 0.74  SF: 0.84  AF: 0.84 | AnF: 1.51  SF: 0.80  AF: -0.76 | NA | NA | NA | NA | NA | NA |
| Simo K, 2014^154^ | NA | NA | SF: 0.68 | NA | NA | NA | NA | NA | NA |
| Singh A. 2020^155^ | AF: 2.67 | AF: 1.50 | AF: 0.68 | NA | NA | NA | NA | NA | NA |
| Singh T, 2018^156^ | AF: >2.67 | AF: >1.5 | AF: >0.67 | NA | NA | NA | NA | NA | NA |
| Sjowall C, 2015^61^ | NA | ? | ? | ? | NA | NA | NA | NA | NA |
| Stauber R, 2018^43^ | NA | NA | NA | NA | NA | NA | AF: 9.10 | NA | NA |
| Staufer K, 2019^157^ | SF: 1.30  AF: 1.30 | NA | NA | NA | SF: 0.31 | NA | SF: 9.10  AF: 9.80 | NA | NA |
| Subasi C, 2015^158^ | AF: 3.00 | AF: 3.00 | AF: 3.00 | AF: 3.00 | ? | NA | NA | NA | NA |
| Sumida Y, 2012^50^ | AF: 1.45 | AF: 1.00 | AF: 1.45 | AF: 2.00 | NA | NA | NA | NA | NA |
| Takeuchi H, 2018^73^ | SF: 1.72  AF: 1.41 | NA | NA | NA | NA | NA | NA | NA | NA |
| Tanwar S, 2006^54^ | ? | ? | ? | ? | NA | NA | ? | NA | NA |
| Thanapirom K, 2017^72^ | ? | ? | NA | NA | NA | NA | NA | NA | NA |
| Tomeno W, 2019^159^ | AF: 3.25 | NA | NA | NA | NA | NA | NA | NA | NA |
| Treeprasertsuk S, 2016^160^ | AF: 1.30 | NA | AF: 1.46 | AF: 2.00 | NA | NA | NA | NA | NA |
| Uy D, 2011^26^ | ? | ? | NA | ? | NA | NA | NA | NA | NA |
| Wong V, 2010^56^ | AF: 0.50 | AF: 1.30 | AF: 1.46 | AF: 2.00 | NA | NA | NA | NA | NA |
| Xun Y, 2012^58^ | AF: 1.30 | AF: 0.50 | AF: 1.46 | AF: 2.00 | NA | NA | NA | NA | NA |
| Yang M, 2019^161^ | AF: 2.67 | AF: 0.98 | AF: 0.675 | AF: 2.00 | ? | NA | NA | NA | ? |
| Yoneda M, 2013^29^ | AF: 2.67 | NA | AF: 0.73 | AF: 3.00 | NA | NA | NA | NA | NA |
| Younes R, 2021^162^ | ? | ? | ? | ? | NA | NA | NA | ? | NA |
| Zhou Y, 2019^79^ | ? | ? | ? | ? | NA | NA | NA | NA | NA |
| Zou C, 2019^19^ | ? | ? | ? | ? | NA | NA | NA | NA | NA |

*AF advanced fibrosis, AnF any fibrosis; APRI aspartate aminotransferase to platelet ratio index; BARD score body mass index, aspartate aminotransferase/alanine aminotransferase ratio, diabetes score; ELF enhanced liver fibrosis; FIB-4 fibrosis index-4; NFS Non-alcoholic fatty liver disease fibrosis score; NA not applicable, SF significant fibrosis;? not responded*

# **Supplementary Table 5.** Risk of bias assessment of included studies using the QUADAS 2 tool.

| **First author (year publication) ^ref^** | **Patients’ selection** | **Index test** | **Reference standard** | **Flow and timing** |
| --- | --- | --- | --- | --- |
| Abe M, 2014(131) | Unclear | Unclear | Low | Unclear |
| Adams L, 2008(112) | Unclear | Low | Low | Unclear |
| Adams L, 2011(16) | Low | Low | Unclear | Low |
| Ahmed Z, 2016(37) | Unclear | Unclear | Low | Unclear |
| Aida Y, 2015(39) | Unclear | Low | High | Unclear |
| Alkhouri N, 2015(111) | Unclear | Low | High | Low |
| Anam M, 2017(118) | Unclear | Unclear | Low | High |
| Angelidi A, 2017(88) | Low | Low | Unclear | Unclear |
| Angulo P, 2014(127) | Low | Low | High | Low |
| Angulo P, 2007(48) | Low | Low | Low | Unclear |
| Anstee Q, 2019(71) | Unclear | Low | Low | Low |
| Amernia B, 2021(2) | Low | Low | Low | Low |
| Arora S, 2016(113) | Low | Unclear | Unclear | Unclear |
| Aykut U, 2014(34) | Unclear | Low | Low | Unclear |
| Balakrishnan M, 2018(10) | Low | Low | Low | High |
| Balakrishnan M, 2021(3) | Low | Low | Low | Low |
| Barritt A, 2019(11) | Unclear | Low | Unclear | Low |
| Boursier J, 2016(51) | Low | Low | Unclear | Low |
| Boursier J, 2017(89) | Unclear | Low | Low | Unclear |
| Boursier J, 2019(90) | Unclear | Low | Low | Unclear |
| Brandman D, 2017(124) | Unclear | Low | Low | Unclear |
| Bril F, 2020(123) | Unclear | Low | Low | Low |
| Broussier T, 2020(80) | Low | Low | Low | Low |
| Cales P, 2009(5) | Low | High | Low | Unclear |
| Cales P, 2010(8) | Low | Low | High | Unclear |
| Cebreiros I, 2014(21) | Low | Unclear | Low | Unclear |
| Cengiz M, 2015(40) | Unclear | Low | Low | Low |
| Chan W, 2014(132) | Unclear | Unclear | Low | Low |
| Chowdhury S, 2013(27) | Low | Low | Unclear | Low |
| Cichoz-Lach H, 2012(22) | Low | Low | Low | Low |
| Cui J, 2015(41) | Unclear | Unclear | Low | Low |
| de Carli M, 2020(134) | Unclear | Low | Unclear | Low |
| de Cleva R, 2016(52) | Low | Low | Low | Low |
| Demir M, 2011(24) | Unclear | Low | Low | Unclear |
| Demir M,2013(28) | Low | Low | Low | Low |
| Dincses E, 2015(42) | Low | Low | Low | Low |
| Drolz A, 2017(35) | Unclear | Low | Low | Low |
| Dvorak K, 2014(36) | Unclear | Unclear | Low | Low |
| Eddowes P, 2019(114) | Unclear | Low | Low | High |
| Fagan K, 2015(115) | High | Low | Unclear | High |
| Francque S, 2012(23) | Low | Unclear | Low | Low |
| Fujii H, 2009(6) | Unclear | Low | Unclear | Low |
| Fujii H, 2009(135) | Low | Low | Unclear | Low |
| Gallego-Duran R, 2012(91) | Unclear | High | Low | Unclear |
| Guha I, 2008(59) | Unclear | Low | Low | Low |
| Guillaume M, 2019(130) | Low | Low | Low | Unclear |
| Guturu P, 2008(119) | Low | High | Low | Unclear |
| Harrison S, 2008(70) | High | Low | Unclear | High |
| Hagström H, 2019(87) | Low | Low | Low | Low |
| Huang C, 2019(103) | Unclear | Low | Low | Unclear |
| Inadomi C, 2020(136) | Low | Low | Low | Unclear |
| Isgro M, 2014(50) | Unclear | Low | Low | Low |
| Itoh Y, 2018(66) | Unclear | Low | Low | Low |
| Joo S, 2017(104) | Low | Low | High | Low |
| Joo, S 2015(92) | Unclear | Unclear | Low | Unclear |
| Jouness R, 2016(93) | Unclear | Low | Low | High |
| Kao W, 2020(105) | Low | Low | Unclear | Low |
| Kawamura, 2015(43) | Low | Unclear | Low | Low |
| Kim D, 2011(106) | Unclear | Unclear | Low | Low |
| Kim D, 2013(137) | Low | Unclear | Low | Unclear |
| Kobayashi N, 2017(58) | Low | Low | High | Low |
| Kolhe K, 2019(72) | Low | Low | Unclear | Low |
| Kosick H, 2019(116) | Unclear | Low | Unclear | Low |
| Kruger F, 2008(120) | Low | Low | Low | Low |
| Kruger F, 2011(125) | Low | Low | Low | High |
| Kumar R, 2013(33) | Unclear | High | Low | Unclear |
| Labenz C, 2018(67) | Low | Low | Low | Low |
| Lambrecht J, 2019(94) | Unclear | Low | Low | High |
| Lang S, 2020(83) | Low | Low | Unclear | Low |
| Lardi L, 2020(139) | Low | Low | Low | Low |
| Lassailly G, 2011(17) | Unclear | Low | Low | Unclear |
| Le P, 2018(128) | Unclear | Unclear | Low | High |
| Lee T, 2013(29) | Unclear | Low | Low | Low |
| Liu W, 2020(84) | Low | Low | Unclear | Low |
| Loaeza-del-Castillo, 2008(81) | Low | Low | Low | Unclear |
| Loong T, 2017(60) | Low | Low | Unclear | Low |
| Luger M, 2016(95) | Unclear | Low | Low | High |
| Mahadeva S, 2013(30) | Unclear | High | Low | Low |
| Marella H, 2019(129) | Unclear | Low | Low | Low |
| McPherson S, 2010(9) | Low | Low | Low | Unclear |
| McPherson S, 2017(61) | Low | Unclear | Low | Low |
| McPherson, 2013(31) | Low | Low | Unclear | Low |
| Meneses D, 2020(82) | Low | Low | Unclear | Low |
| Miao C, 2010(117) | Low | Low | Low | Unclear |
| Miele L, 2015(96) | Low | Low | Low | Unclear |
| Miele L, 2017(62) | Low | Low | Low | Low |
| Miller A, 2019(57) | Low | Low | Low | Unclear |
| Miller M, 2010(15) | Unclear | High | Low | Unclear |
| Munteanu M, 2016(97) | Low | Low | Unclear | Unclear |
| Nascimbeni, 2015(98) | Unclear | Low | Unclear | Unclear |
| Nassif AT, 2017(63) | Low | Low | Unclear | Unclear |
| Okajima A, 2017(64) | Low | Low | Unclear | Low |
| Pastor-Ramirez, 2017(99) | Low | Low | Low | Low |
| Pathik P, 2015(44) | Unclear | Low | Low | Unclear |
| Peleg N, 2017(65) | Low | Low | Low | Unclear |
| Pérez-Gutiérrez O, 2013(32) | Low | Unclear | Low | Unclear |
| Petta S, 2015(45) | Unclear | Low | Low | Low |
| Petta S, 2017(138) | Low | Low | Unclear | Low |
| Pimentel S, 2010(12) | Unclear | Low | Unclear | Low |
| Polyzos S, 2019(73) | Low | Low | Low | Low |
| Prasad S, 2020(107) | Low | Low | Unclear | Low |
| Qureshi K, 2008(86) | Unclear | Low | Low | Unclear |
| Raszeja-Wyszomirska J, 2010(13) | Unclear | Low | Low | Low |
| Rath M, 2016(53) | Low | Low | Low | Unclear |
| Ratziu C, 2006(26) | Low | Unclear | Unclear | Unclear |
| Ratziu V, 2004(1) | Low | Low | Low | Low |
| Ruffillo G, 2011(18) | Low | Low | Low | Low |
| Saez E, 2017(100) | Unclear | Low | Low | High |
| Sebastiani G, 2011(19) | Low | Low | Low | Unclear |
| Seth A, 2016(68) | Unclear | Low | Low | Low |
| Shah A, 2009(7) | Unclear | Unclear | Low | Low |
| Shaheen A, 2016(121) | Low | Low | Unclear | Unclear |
| Shima T, 2019(74) | Low | Low | Unclear | Low |
| Shoji H, 2016(54) | Unclear | Low | High | Low |
| Shukla A, 2015(46) | Low | Low | Low | Unclear |
| Siddiqui M, 2016(55) | Low | Low | Low | Low |
| Siddiqui M, 2019(75) | Low | Low | High | Unclear |
| Simo K, 2014(38) | Unclear | Unclear | Low | Low |
| Singh A. 2020(85) | Low | Low | Low | Low |
| Singh T, 2018(126) | Unclear | Low | Low | Low |
| Sjowall C, 2015(47) | Low | Low | Unclear | Low |
| Stauber R, 2018(101) | Unclear | Low | Low | Unclear |
| Staufer K, 2019(76) | Low | Low | Low | Low |
| Subasi C, 2015(49) | Low | Low | Low | Unclear |
| Sumida Y, 2012(20) | Unclear | Low | Unclear | Low |
| Takeuchi H, 2018(69) | Low | Low | Unclear | Unclear |
| Tanwar S, 2006(102) | Low | Low | Unclear | High |
| Thanapirom K, 2017(122) | Low | Low | Unclear | Low |
| Tomeno W, 2019(77) | Low | Low | Low | Low |
| Treeprasertsuk S, 2016(56) | Low | Low | Low | Low |
| Uy D, 2011(108) | Unclear | Low | Low | Unclear |
| Wong V, 2010(14) | Low | Unclear | Unclear | Low |
| Xun Y, 2012(25) | Low | Low | Unclear | Unclear |
| Yang M, 2019(78) | Low | Low | Low | Low |
| Yoneda M, 2013(109) | Low | Low | Low | Low |
| Younes R, 2021(4) | Unclear | Low | Low | Unclear |
| Zhou Y, 2019(110) | Low | Low | Unclear | Low |
| Zou C, 2019(133) | Unclear | Low | Low | Unclear |

**Supplementary Table 6.** Assessment of the applicability concerns of the included studies using

| **First author,** | **Patient selection** | **Index test** | **Reference standard** |
| --- | --- | --- | --- |
| **(year publication) ^ref^** |  |  |  |
| Abe M, 2014(131) | Unclear | Low | Unclear |
| Adams L, 2008(112) | Unclear | Low | Low |
| Adams L, 2011(16) | Unclear | Low | Low |
| Ahmed Z, 2016(37) | Unclear | Unclear | High |
| Aida Y, 2015(39) | Low | Low | Unclear |
| Alkhouri N, 2015(111) | Unclear | High | Low |
| Anam M, 2017(118) | Unclear | Low | Low |
| Angelidi A, 2017(88) | Low | Unclear | High |
| Angulo P, 2014(127) | Low | Low | Unclear |
| Angulo P, 2007(48) | Low | Unclear | Low |
| Anstee Q, 2019(71) | Unclear | Unclear | Low |
| Amernia B, 2021(2) | Low | Low | Low |
| Arora S, 2016(113) | Low | Unclear | Low |
| Aykut U, 2014(34) | Low | Unclear | Low |
| Balakrishnan M, 2018(10) | Unclear | Unclear | Low |
| Balakrishnan M, 2021(3) | Low | Low | Low |
| Barritt A, 2019(11) | Low | Low | Low |
| Boursier J, 2016(51) | Low | Low | Unclear |
| Boursier J, 2017(89) | Unclear | High | Low |
| Boursier J, 2019(90) | Low | Low | Unclear |
| Brandman D, 2017(124) | Low | Low | Unclear |
| Bril F, 2020(123) | Unclear | Unclear | Low |
| Broussier T, 2020(80) | Low | Low | Unclear |
| Cales P, 2009(5) | Unclear | High | Low |
| Cales P, 2010(8) | Low | Low | Low |
| Cebreiros I, 2014(21) | Unclear | Low | Low |
| Cengiz M, 2015(40) | Low | Low | Unclear |
| Chan W, 2014(132) | Low | Low | Unclear |
| Chowdhury S, 2013(27) | Low | Unclear | Low |
| Cichoz-Lach H, 2012(22) | Unclear | Low | Low |
| Cui J, 2015(41) | Low | Unclear | Low |
| de Carli M, 2020(134) | Unclear | Low | Unclear |
| de Cleva R, 2016(52) | Unclear | Low | Low |
| Demir M, 2011(24) | Unclear | Unclear | Low |
| Demir M,2013(28) | Low | Low | Low |
| Dincses E, 2015(42) | Low | Low | Unclear |
| Drolz A, 2017(35) | Unclear | Low | Low |
| Dvorak K, 2014(36) | Low | Unclear | Low |
| Eddowes P, 2019(114) | Unclear | Low | Low |
| Fagan K, 2015(115) | High | Low | Unclear |
| Francque S, 2012(23) | Unclear | Low | Low |
| Fujii H, 2009(6) | Low | Low | Low |
| Fujii H, 2009(135) | Unclear | Low | Low |
| Gallego-Duran R, 2012(91) | Unclear | High | Low |
| Guha I, 2008(59) | Low | Low | Low |
| Guillaume M, 2019(130) | Low | Low | Unclear |
| Guturu P, 2008(119) | Low | High | Low |
| Harrison S, 2008(70) | High | Low | High |
| Hagström H, 2019(87) | Low | Low | Unclear |
| Huang C, 2019(103) | Unclear | Low | Unclear |
| Inadomi C, 2020(136) | Unclear | Low | Unclear |
| Isgro M, 2014(50) | Unclear | Low | High |
| Itoh Y, 2018(66) | Unclear | Low | Low |
| Joo S, 2017(104) | Unclear | Low | Unclear |
| Joo, S 2015(92) | Unclear | Unclear | Low |
| Jouness R, 2016(93) | Unclear | Low | Unclear |
| Kao W, 2020(105) | Unclear | Low | Low |
| Kawamura, 2015(43) | Unclear | High | Low |
| Kim D, 2011(106) | Unclear | Unclear | Low |
| Kim D, 2013(137) | Unclear | Unclear | Low |
| Kobayashi N, 2017(58) | Low | Low | Unclear |
| Kolhe K, 2019(72) | Low | Low | Unclear |
| Kosick H, 2019(116) | Unclear | Unclear | Low |
| Kruger F, 2008(120) | Low | Low | Low |
| Kruger F, 2011(125) | Unclear | Low | Unclear |
| Kumar R, 2013(33) | Low | Unclear | High |
| Labenz C, 2018(67) | Low | Low | Low |
| Lambrecht J, 2019(94) | Low | Low | Low |
| Lang S, 2020(83) | Low | Low | Low |
| Lardi L, 2020(139) | Low | Low | Low |
| Lassailly G, 2011(17) | Unclear | Low | Low |
| Le P, 2018(128) | Unclear | Low | Unclear |
| Lee T, 2013(29) | Unclear | Low | Low |
| Liu W, 2020(84) | Low | Low | Low |
| Loaeza-del-Castillo, 2008(81) | Unclear | High | Low |
| Loong T, 2017(60) | Low | Low | Unclear |
| Luger M, 2016(95) | High | Low | Unclear |
| Mahadeva S, 2013(30) | Unclear | High | Low |
| Marella H, 2019(129) | Unclear | Low | Low |
| McPherson S, 2010(9) | Low | Low | Low |
| McPherson S, 2017(61) | Unclear | Low | Low |
| McPherson, 2013(31) | Low | Low | Low |
| Meneses D, 2020(82) | Low | Low | Unclear |
| Miao C, 2010(117) | Low | Low | Low |
| Miele L, 2015(96) | Low | Low | Unclear |
| Miele L, 2017(62) | Low | Low | Low |
| Miller A, 2019(57) | Unclear | Unclear | Low |
| Miller M, 2010(15) | Unclear | Unclear | Low |
| Munteanu M, 2016(97) | Low | Low | High |
| Nascimbeni, 2015(98) | Unclear | Low | High |
| Nassif AT, 2017(63) | Low | Low | High |
| Okajima A, 2017(64) | Low | Low | Unclear |
| Pastor-Ramirez, 2017(99) | Low | Unclear | Low |
| Pathik P, 2015(44) | Unclear | Low | Low |
| Peleg N, 2017(65) | Low | Low | Low |
| Pérez-Gutiérrez O, 2013(32) | Low | Unclear | Low |
| Petta S, 2015(45) | Unclear | Low | Unclear |
| Petta S, 2017(138) | Low | Low | Unclear |
| Pimentel S, 2010(12) | Unclear | Low | Low |
| Polyzos S, 2019(73) | Low | Low | Low |
| Prasad S, 2020(107) | Low | Low | Low |
| Qureshi K, 2008(86) | Low | Low | Low |
| Raszeja-Wyszomirska J, 2010(13) | Unclear | Unclear | Low |
| Rath M, 2016(53) | Low | Low | Low |
| Ratziu C, 2006(26) | Low | Unclear | Unclear |
| Ratziu V, 2004(1) | Low | Unclear | Low |
| Ruffillo G, 2011(18) | Low | Low | Low |
| Saez E, 2017(100) | Unclear | Low | High |
| Sebastiani G, 2011(19) | Unclear | Low | Low |
| Seth A, 2016(68) | Low | Low | Unclear |
| Shah A, 2009(7) | Low | Low | Low |
| Shaheen A, 2016(121) | Low | Unclear | Low |
| Shima T, 2019(74) | Low | Low | Unclear |
| Shoji H, 2016(54) | Unclear | Low | High |
| Shukla A, 2015(46) | Low | Unclear | Low |
| Siddiqui M, 2016(55) | Low | Low | Low |
| Siddiqui M, 2019(75) | Low | Low | High |
| Simo K, 2014(38) | Unclear | Unclear | Low |
| Singh A. 2020(85) | Low | Low | Low |
| Singh T, 2018(126) | Unclear | Low | Low |
| Sjowall C, 2015(47) | Low | Low | Unclear |
| Stauber R, 2018(101) | Low | Low | Low |
| Staufer K, 2019(76) | Low | Low | Low |
| Subasi C, 2015(49) | Low | Low | Low |
| Sumida Y, 2012(20) | Low | Low | Low |
| Takeuchi H, 2018(69) | Low | Unclear | Unclear |
| Tanwar S, 2006(102) | Unclear | Low | Unclear |
| Thanapirom K, 2017(122) | Unclear | Low | Low |
| Tomeno W, 2019(77) | Unclear | Unclear | Low |
| Treeprasertsuk S, 2016(56) | Low | Low | Low |
| Uy D, 2011(108) | Unclear | Low | Low |
| Wong V, 2010(14) | Low | Unclear | Low |
| Xun Y, 2012(25) | Unclear | Low | Unclear |
| Yang M, 2019(78) | Low | Low | Low |
| Yoneda M, 2013(109) | Unclear | Low | Low |
| Younes R, 2021(4) | Low | Low | Low |
| Zhou Y, 2019(110) | Low | Low | Unclear |


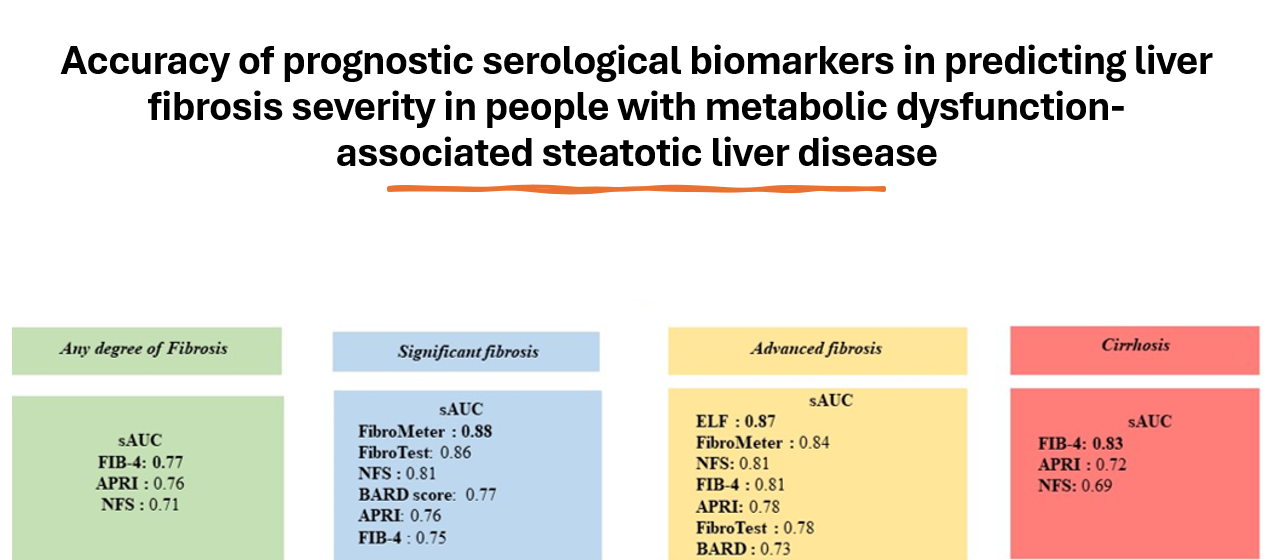


**Supplementary Figure 1.** Algorithm of the prognostic accuracy of serological biomarkers in predicting liver fibrosis severity in people with MASLD

**Meta-analysis figures**

**APRI**

***Diagnosis of any fibrosis (F0 vs F1-F4)***

**
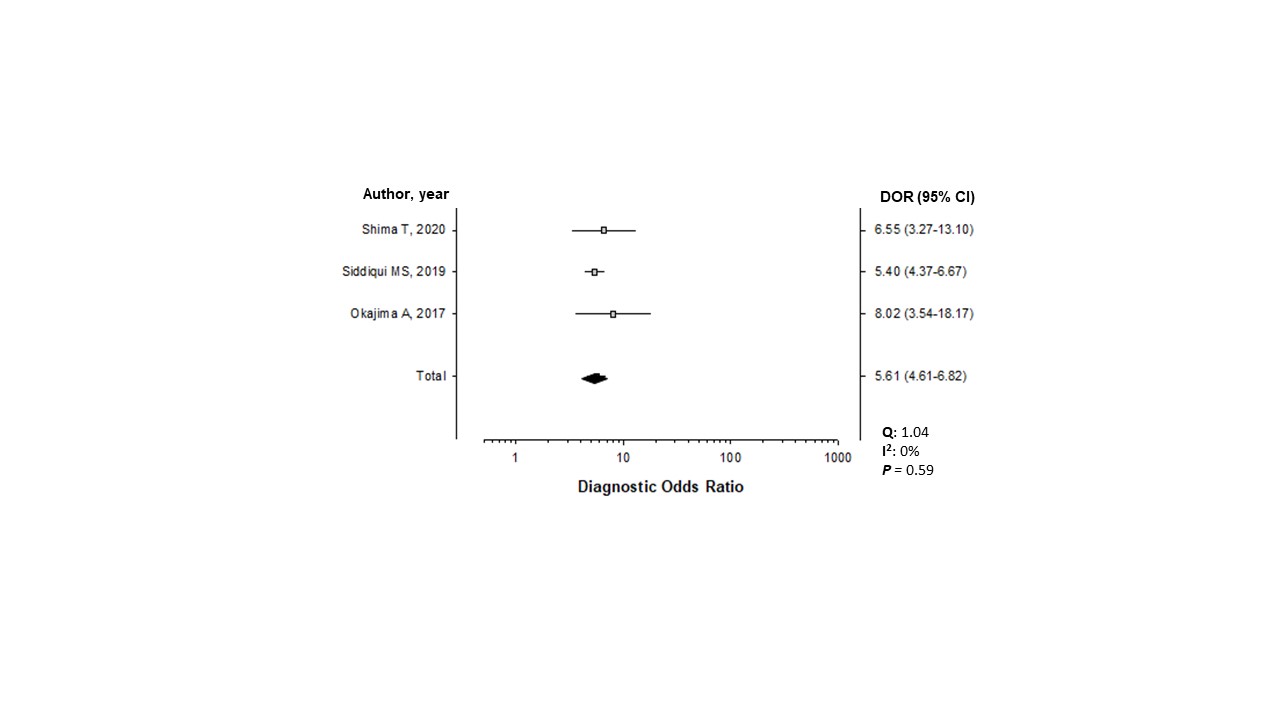
**

# **Supplementary Figure 2.** Forest plot of meta-analysis of diagnostic odds ratio for APRI values in any fibrosis.

**
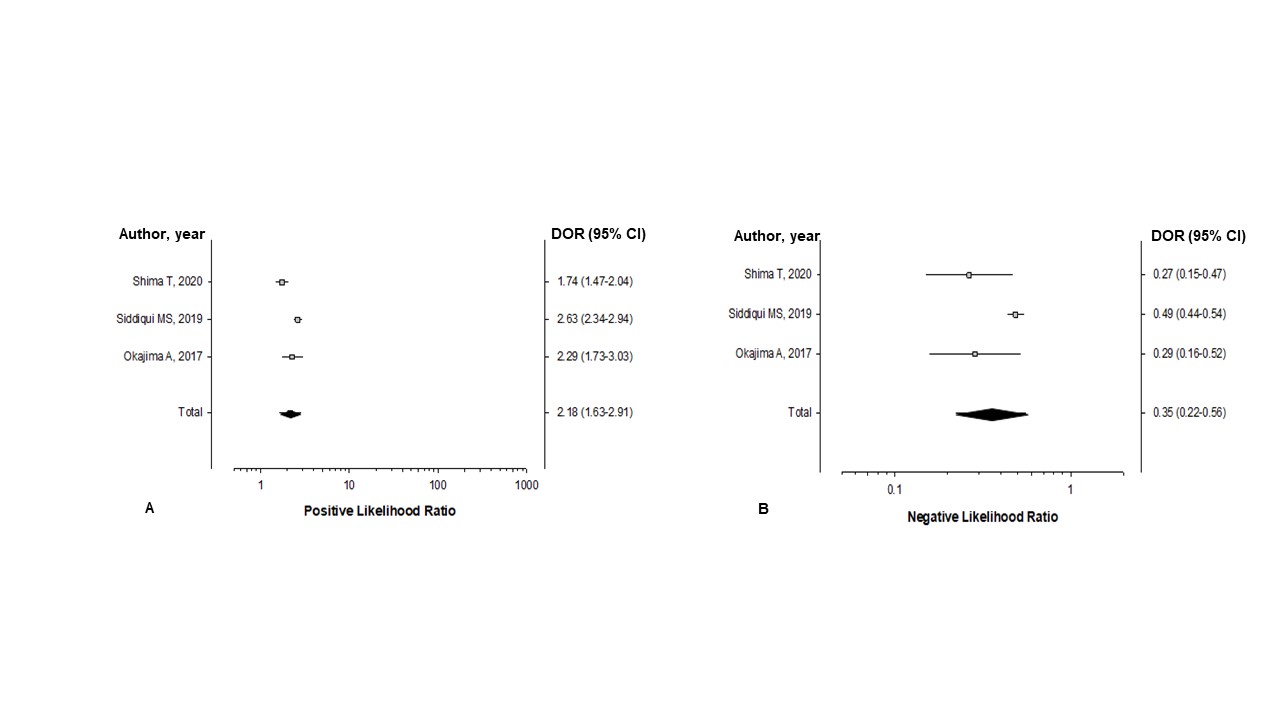
**

# **Supplementary Figure 3.** (A) Forest plot of meta-analysis of positive likelihood ratio and (B) negative likelihood ratio for APRI values in any fibrosis.

***Diagnosis of significant fibrosis (F0-1 vs F2-4)***

**
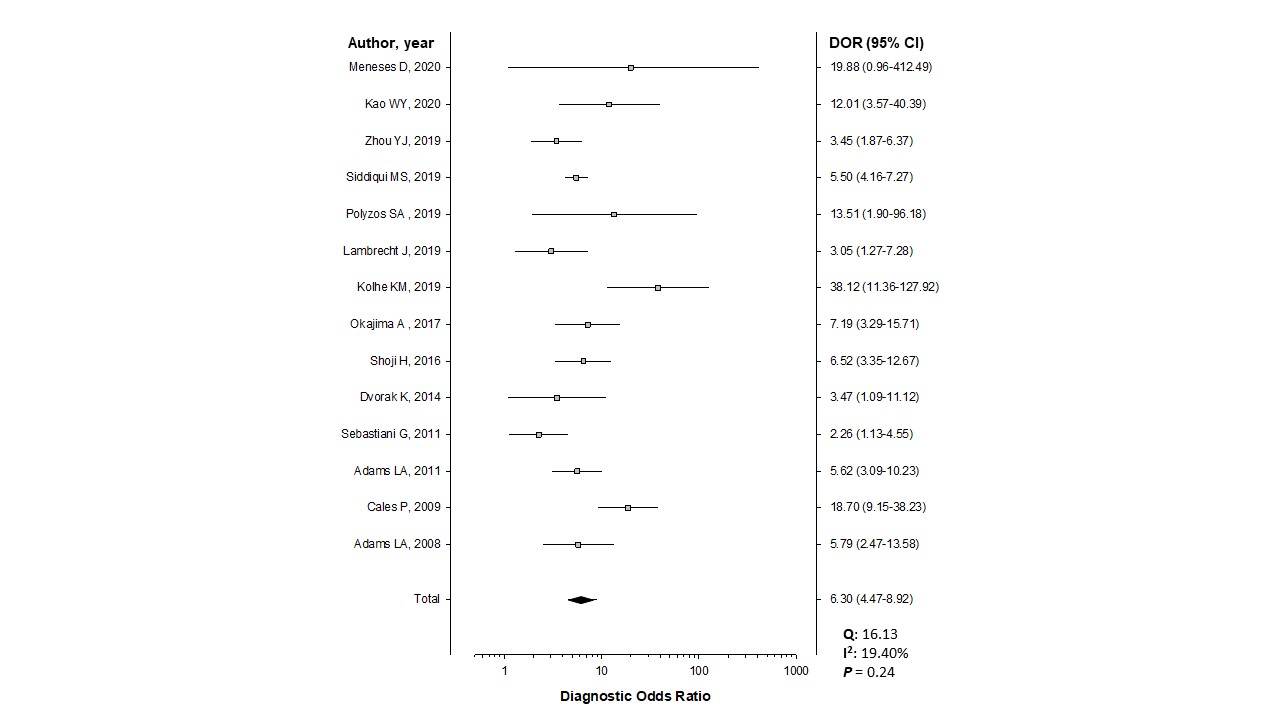
**

# **Supplementary Figure 4.** Forest plot of meta-analysis of diagnostic odds ratio for APRI values in significant fibrosis.

**
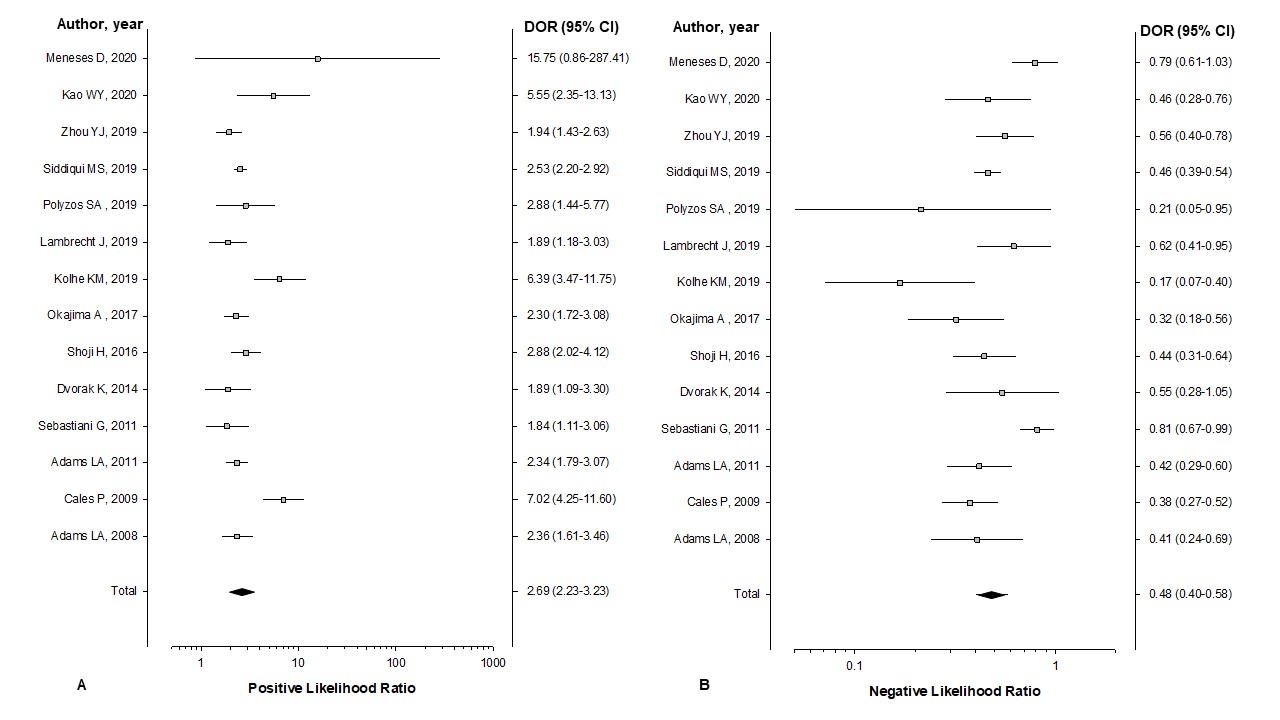
Supplementary Figure 5.** (A) Forest plot of meta-analysis of positive likelihood ratio and (B) negative likelihood ratio for APRI values in significant fibrosis.

***Diagnosis of advanced fibrosis (F0-2 vs F3-4)***

**
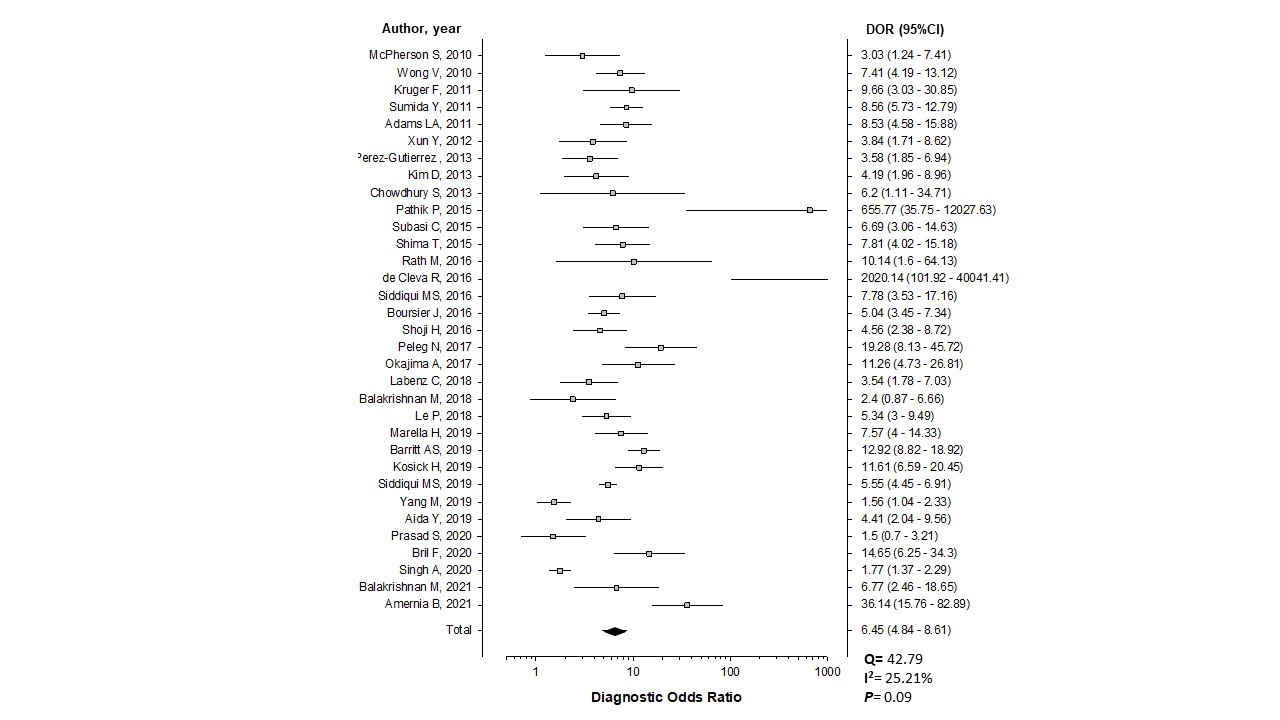
**

# **Supplementary Figure 6.** Forest plot of meta-analysis of diagnostic odds ratio for APRI values in advanced fibrosis.

**
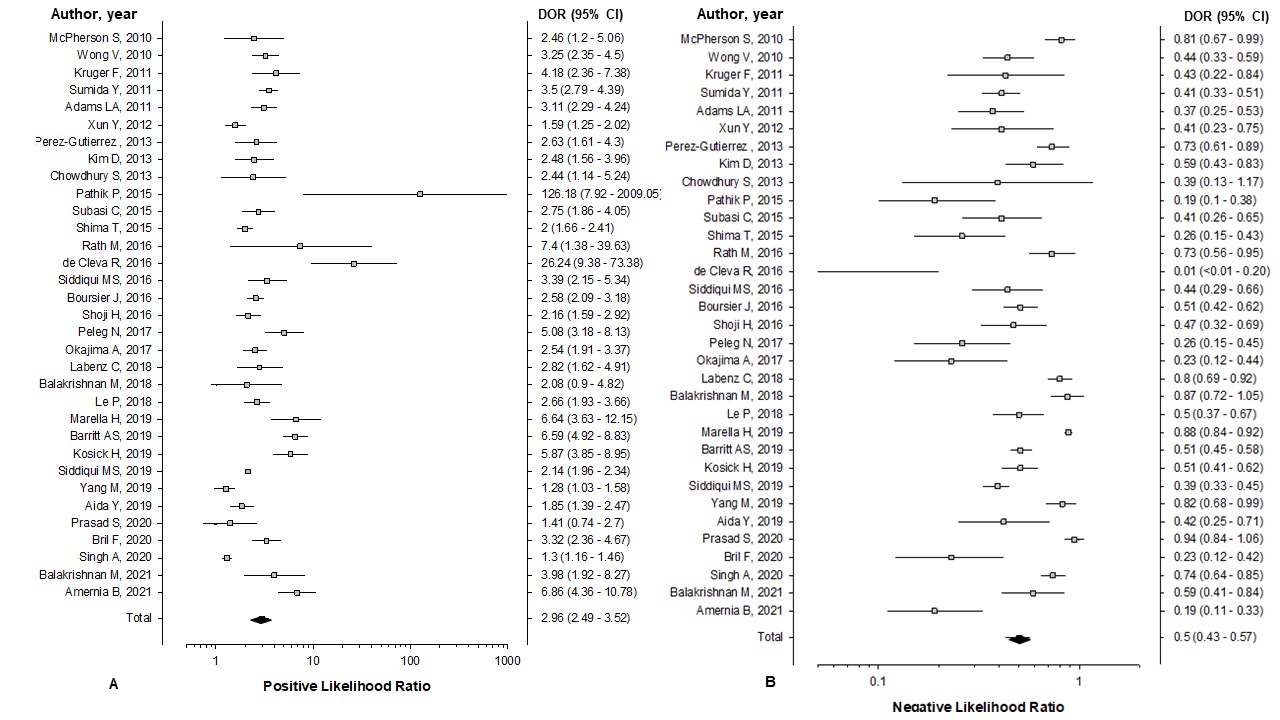
**

# **Supplementary Figure 7.** (A) Forest plot of meta-analysis of positive likelihood ratio and (B) negative likelihood ratio for APRI values in advanced fibrosis.

***Diagnosis of cirrhosis (F0-3 vs F4)***

**
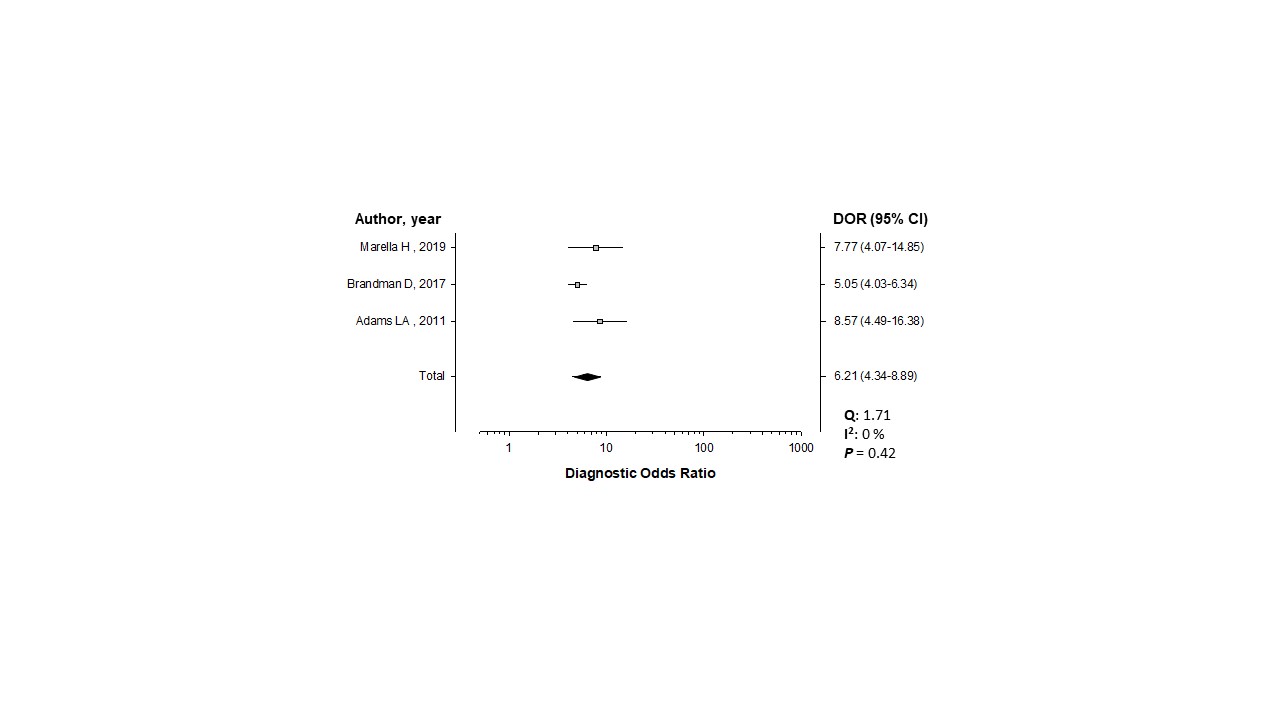
**

#

# **Supplementary Figure 8.** Forest plot of meta-analysis of diagnostic odds ratio for APRI values in cirrhosis.

**
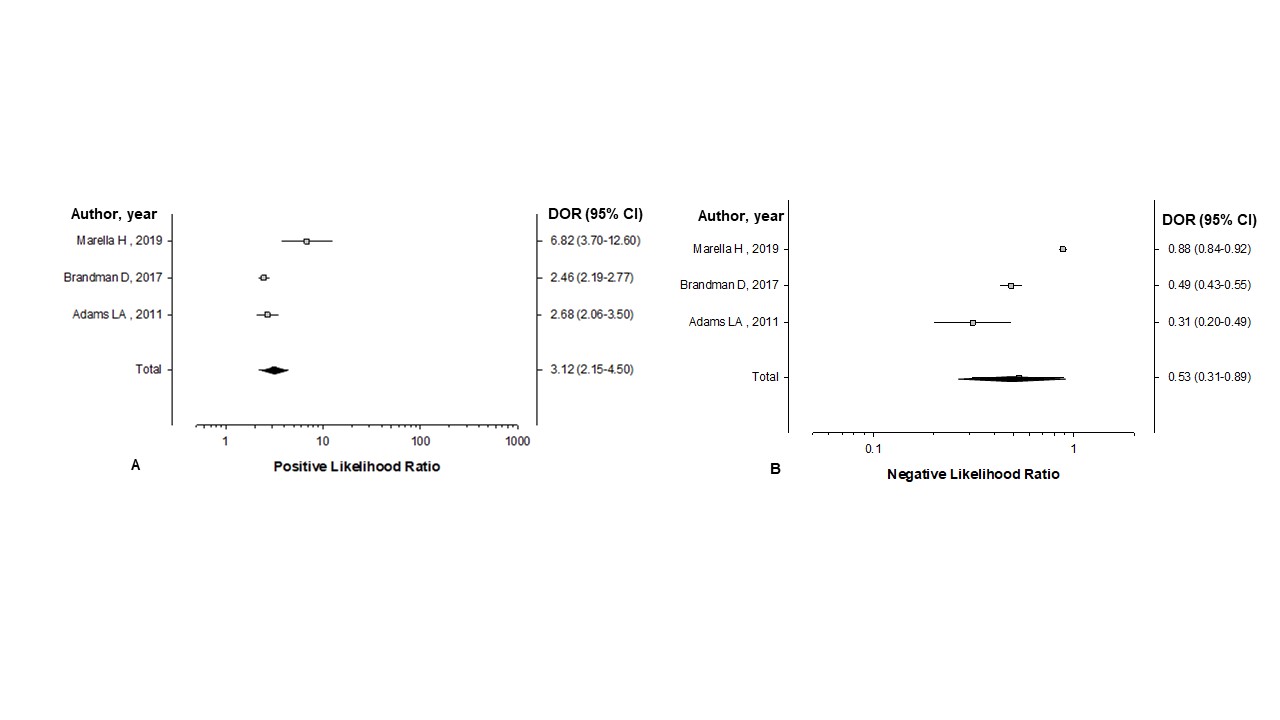
**

# **Supplementary Figure 9.** (A) Forest plot of meta-analysis of positive likelihood ratio and (B) negative likelihood ratio for APRI values in cirrhosis.

**FIB-4**

***Diagnosis of any fibrosis (F0 vs F1-F4)***

**
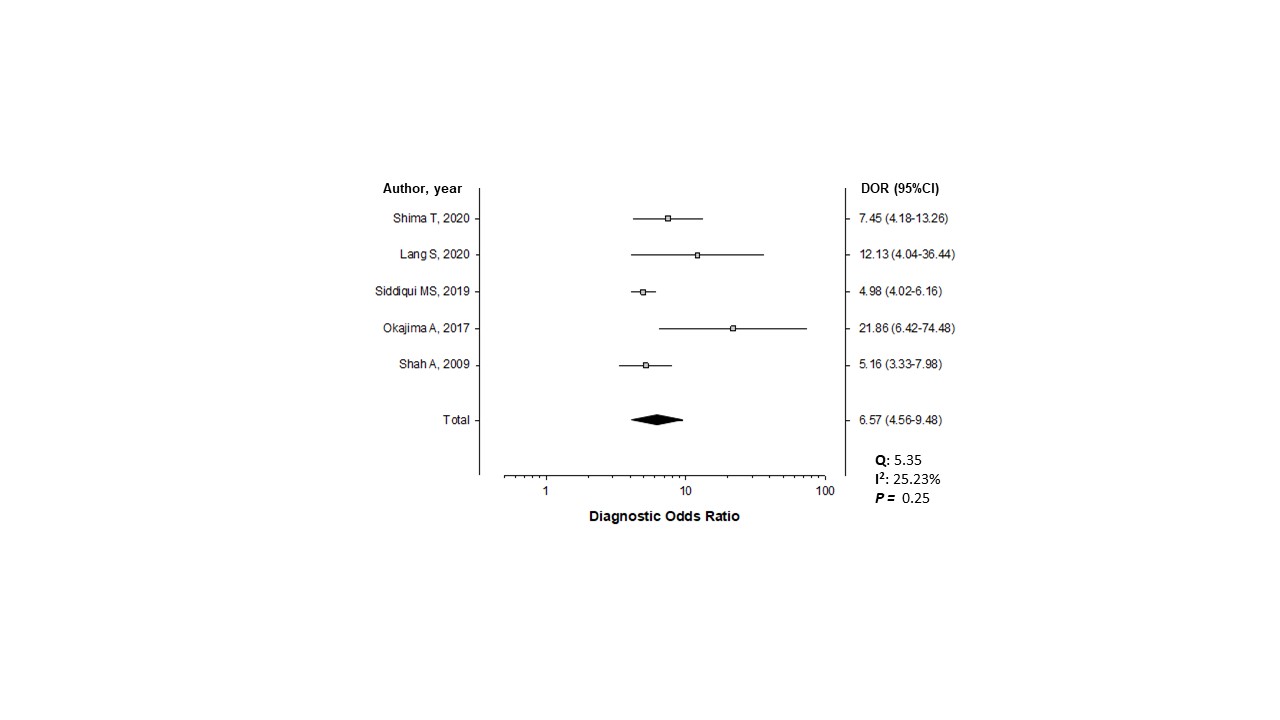
**

# **Supplementary Figure 10.** Forest plot of meta-analysis of diagnostic odds ratio for FIB-4 values in any fibrosis.

**
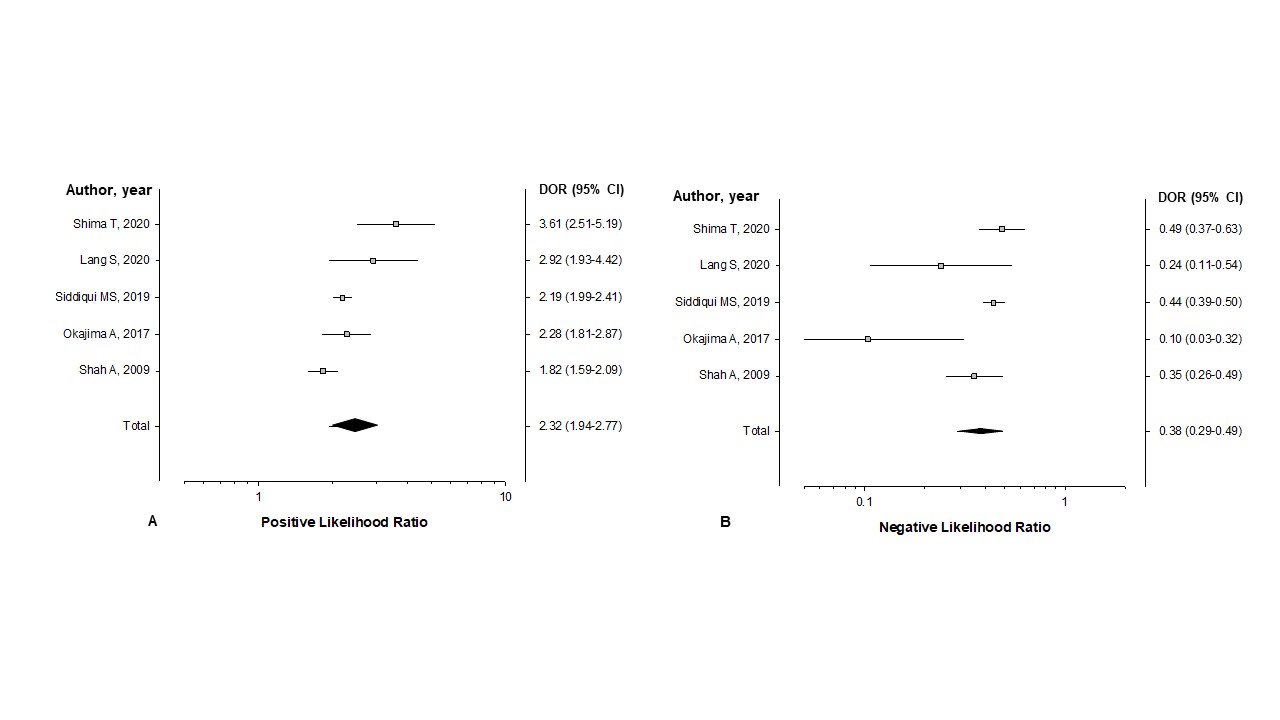
**

# **Supplementary Figure 11.** (A) Forest plot of meta-analysis of positive likelihood ratio and (B) negative likelihood ratio for FIB-4 values in any fibrosis.

***Diagnosis of significant fibrosis (F0-1 vs F2-4)***

**
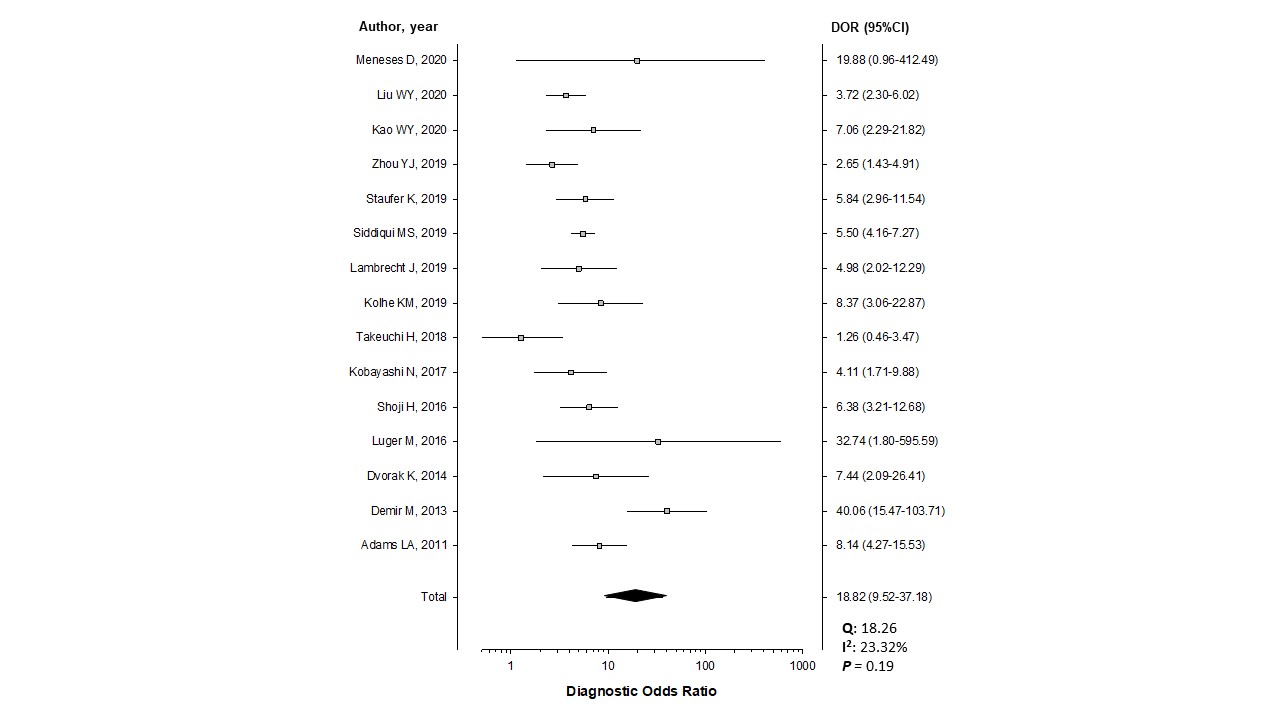
**

**Supplementary Figure 12.** Forest plot of meta-analysis of diagnostic odds ratio for FIB-4 values in significant fibrosis.

**
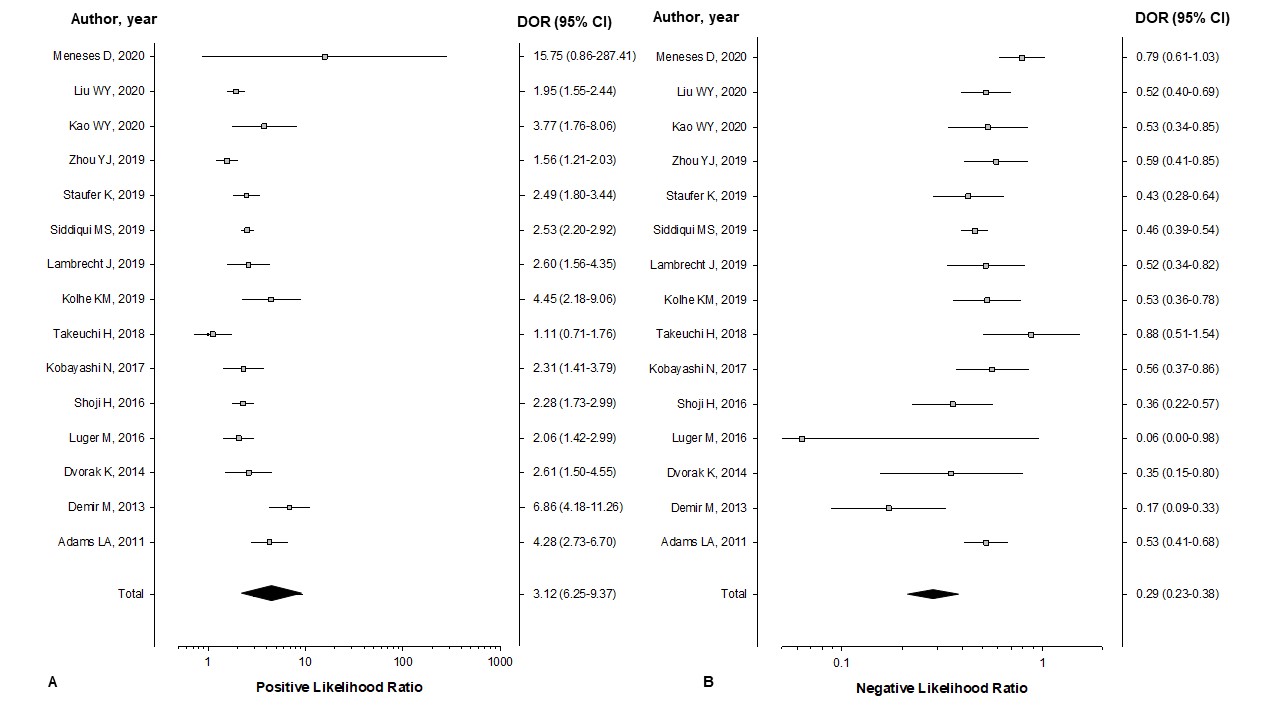
**

# **Supplementary Figure 13.** (A) Forest plot of meta-analysis of positive likelihood ratio and (B) negative likelihood ratio for FIB-4 values in significant fibrosis.

***Diagnosis of advanced fibrosis (F0-2 vs F3-4)***

**
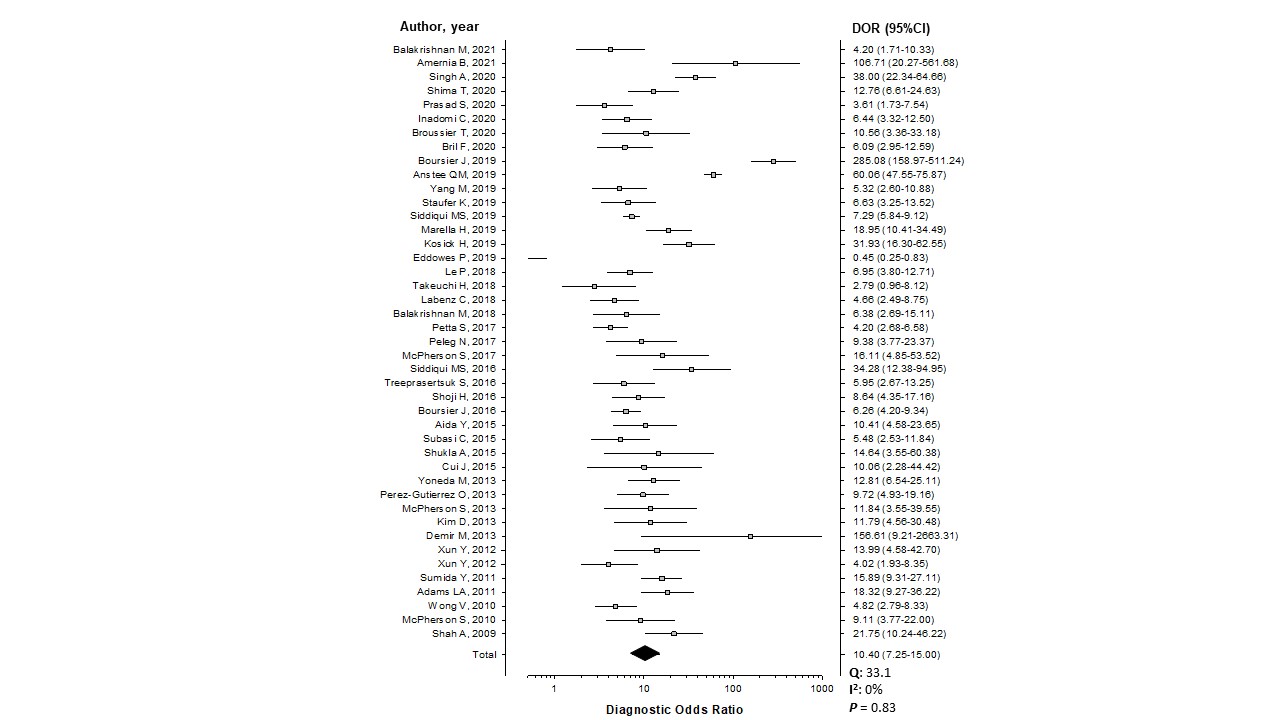
**

# **Supplementary Figure 14.** Forest plot of meta-analysis of diagnostic odds ratio for FIB-4 values in advanced fibrosis.

**
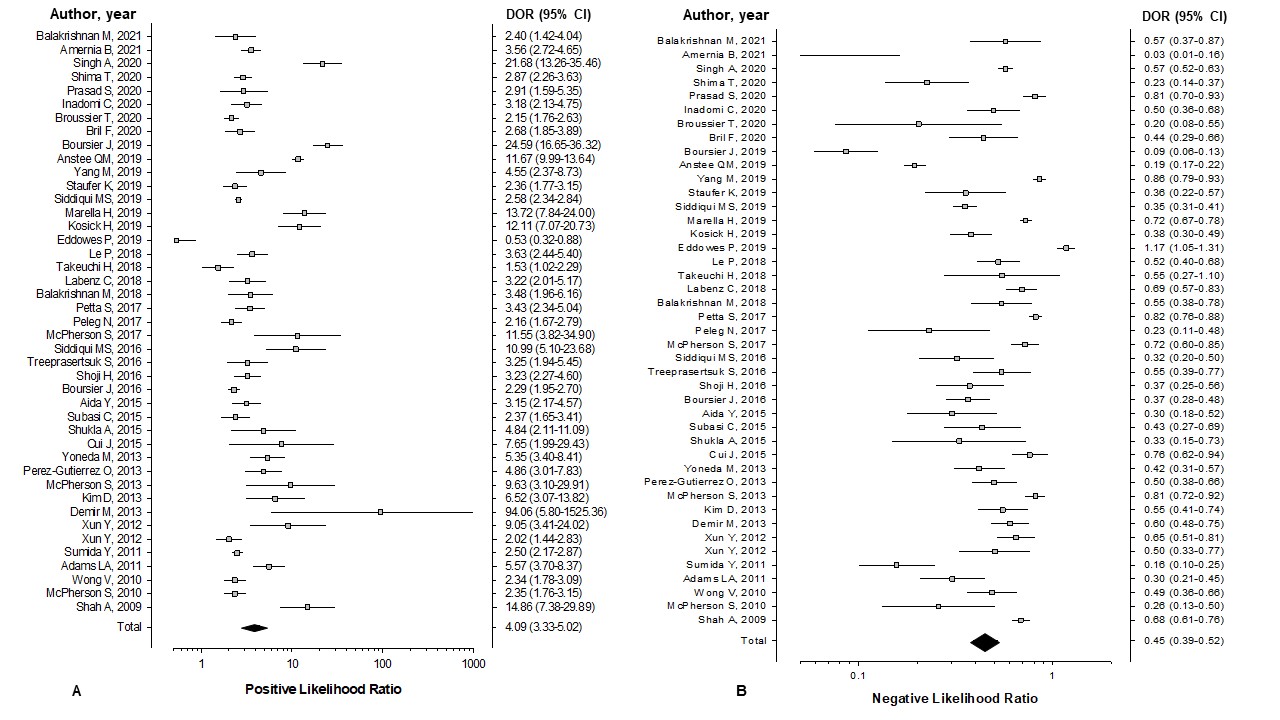
**

# **Supplementary Figure 15.** (A) Forest plot of meta-analysis of positive likelihood and (B) negative likelihood ratio for FIB-4 values in advanced fibrosis.

***Diagnosis of cirrhosis (F0-3 vs F4)***

**
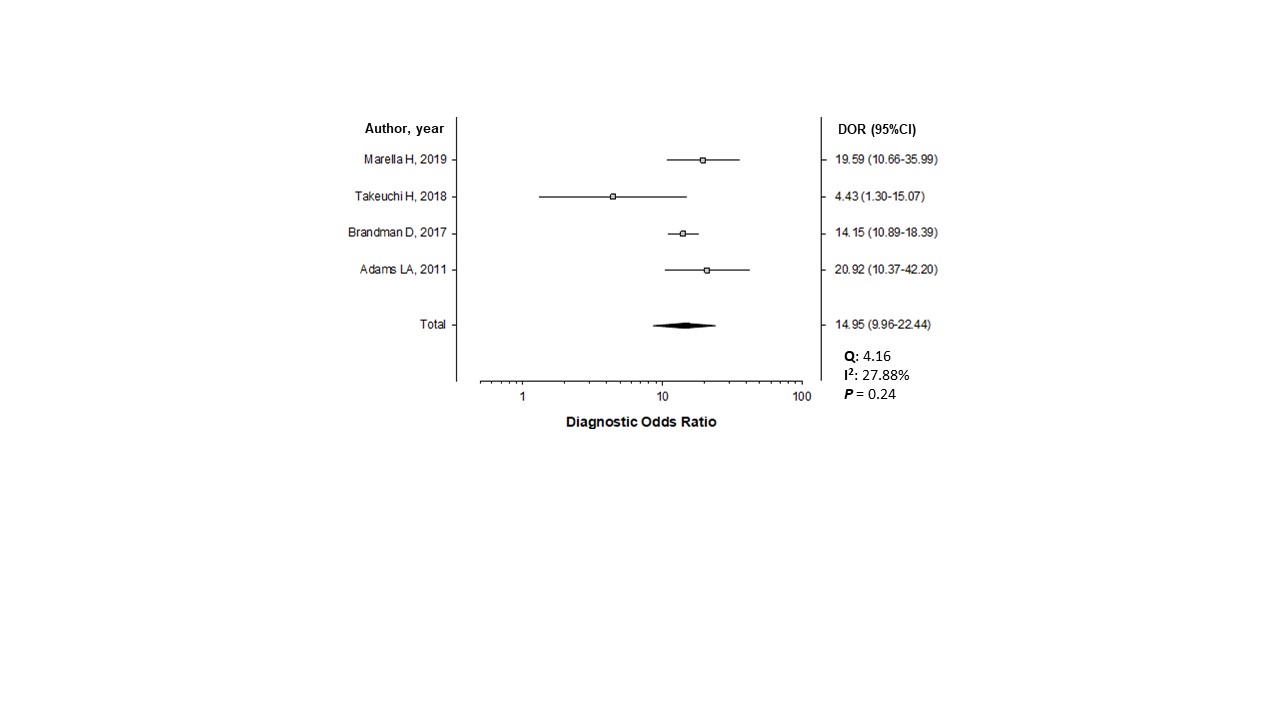
**

# **Supplementary Figure 16.** Forest plot of meta-analysis of diagnostic odds ratio for FIB-4 values in cirrhosis.

# **
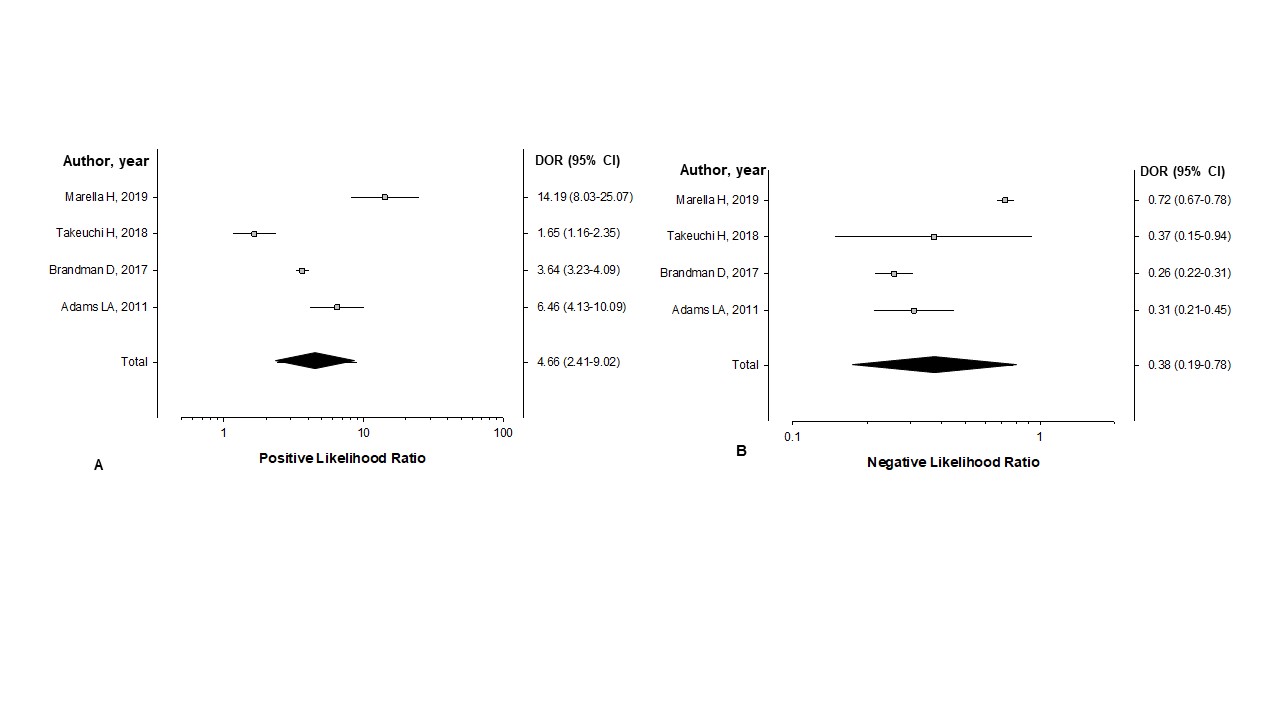
 Supplementary Figure 17.** (A) Forest plot of meta-analysis of positive likelihood ratio and (B) Forest plot of meta-analysis of negative likelihood ratio for FIB-4 values in cirrhosis.

**NFS**

***Diagnosis of any fibrosis (F0 vs F1-F4)***

**
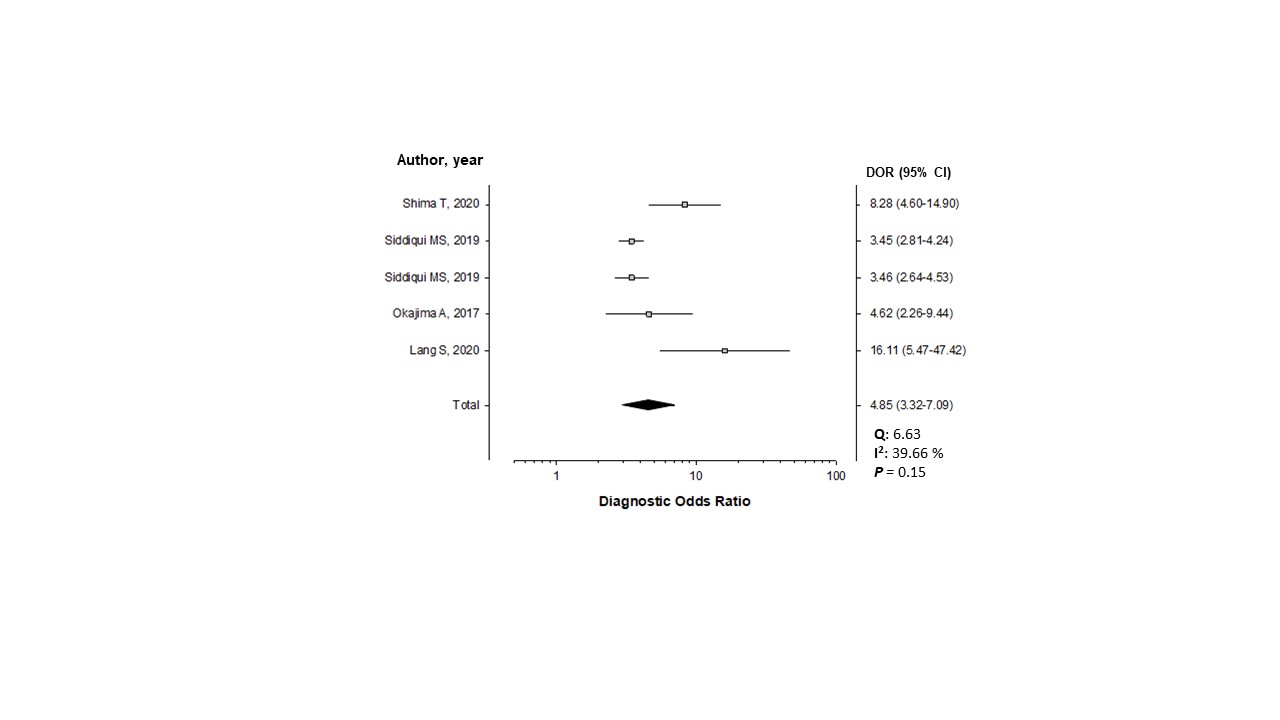
**

# **Supplementary Figure 18.** Forest plot of meta-analysis of diagnostic odds ratio for NFS values in any fibrosis.

**
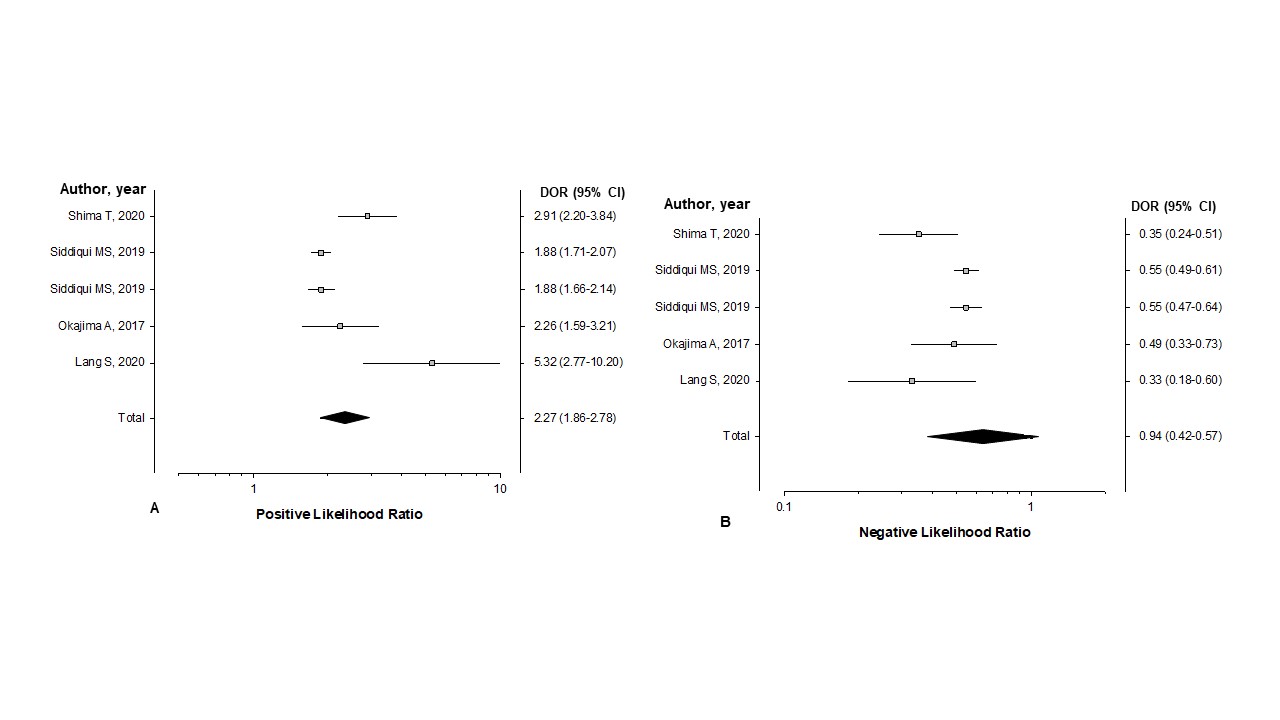
**

# **Supplementary Figure 19.** (A) Forest plot of meta-analysis of positive likelihood ratio and (B) negative likelihood ratio for NFS values in any fibrosis.

***Diagnosis of significant fibrosis (F0-1 vs F2-4)***

**
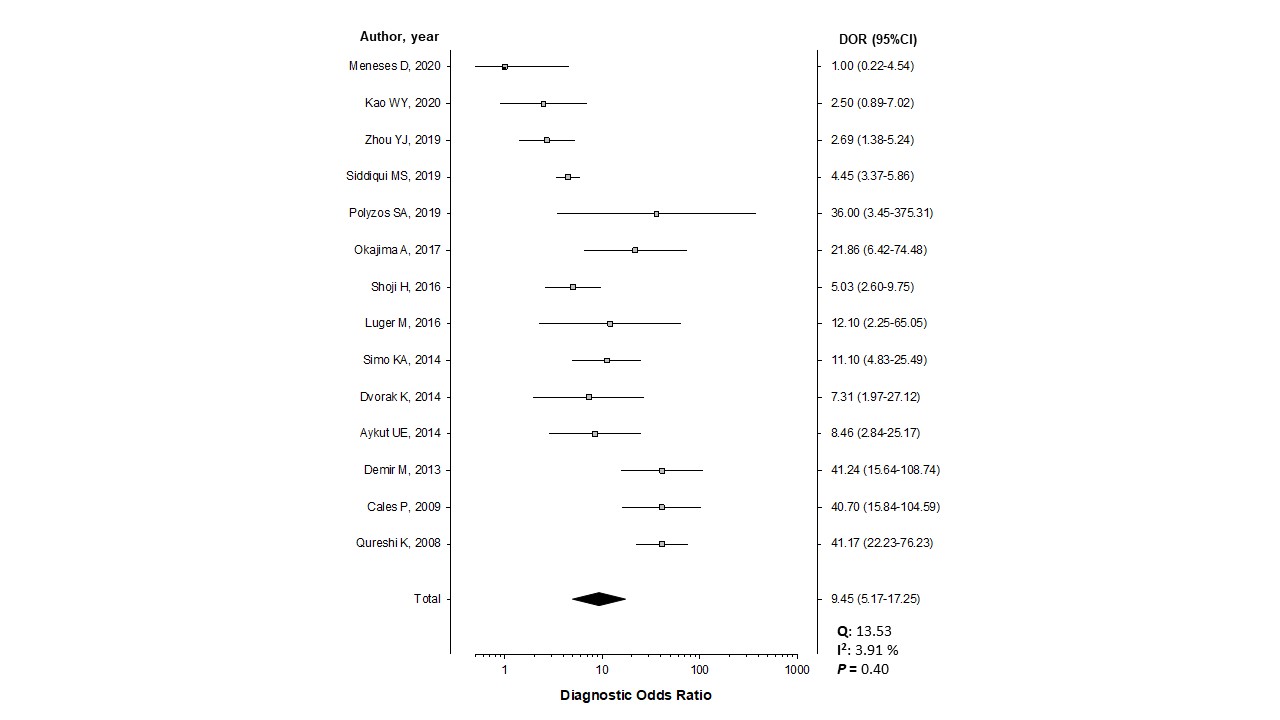
**

# **Supplementary Figure 20.** Forest plot of meta-analysis of diagnostic odds ratio for NFS values in significant fibrosis.

**
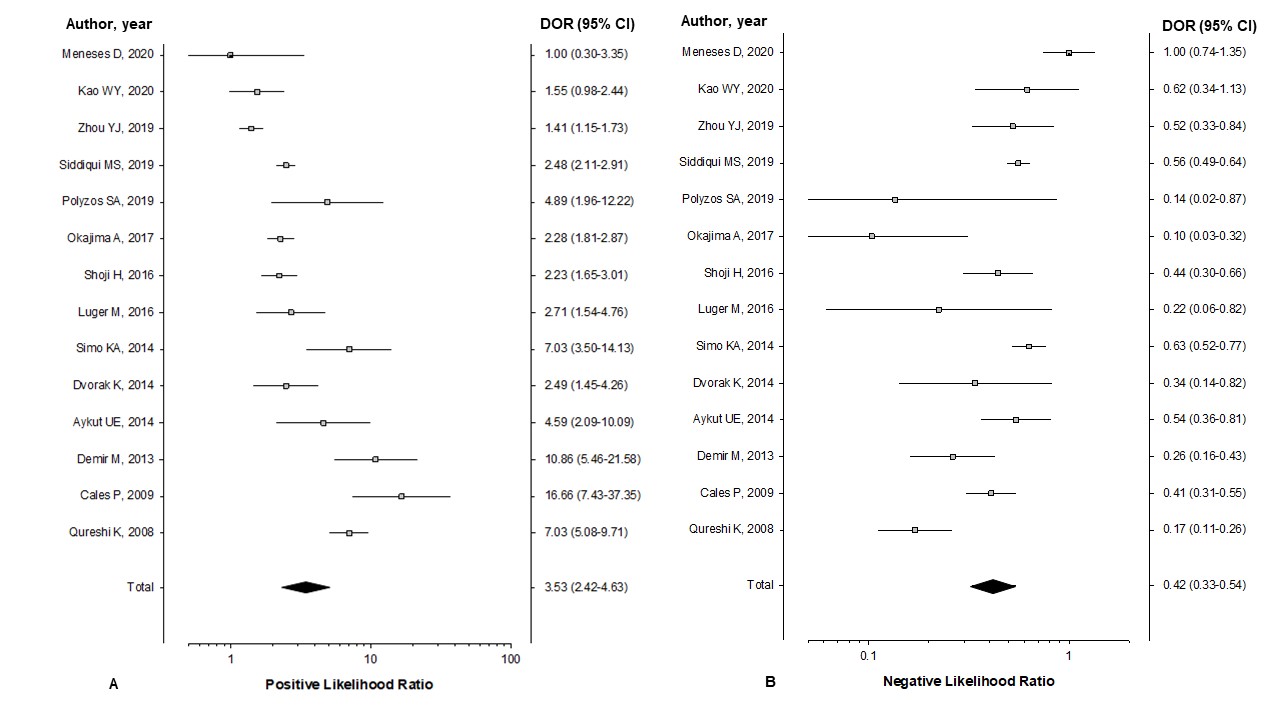
****Supplementary Figure 21.** (A) Forest plot of meta-analysis of positive likelihood ratio and (B) negative likelihood ratio for NFS values in significant fibrosis.

***Diagnosis of advanced fibrosis (F0-2 vs F3-4)***

**
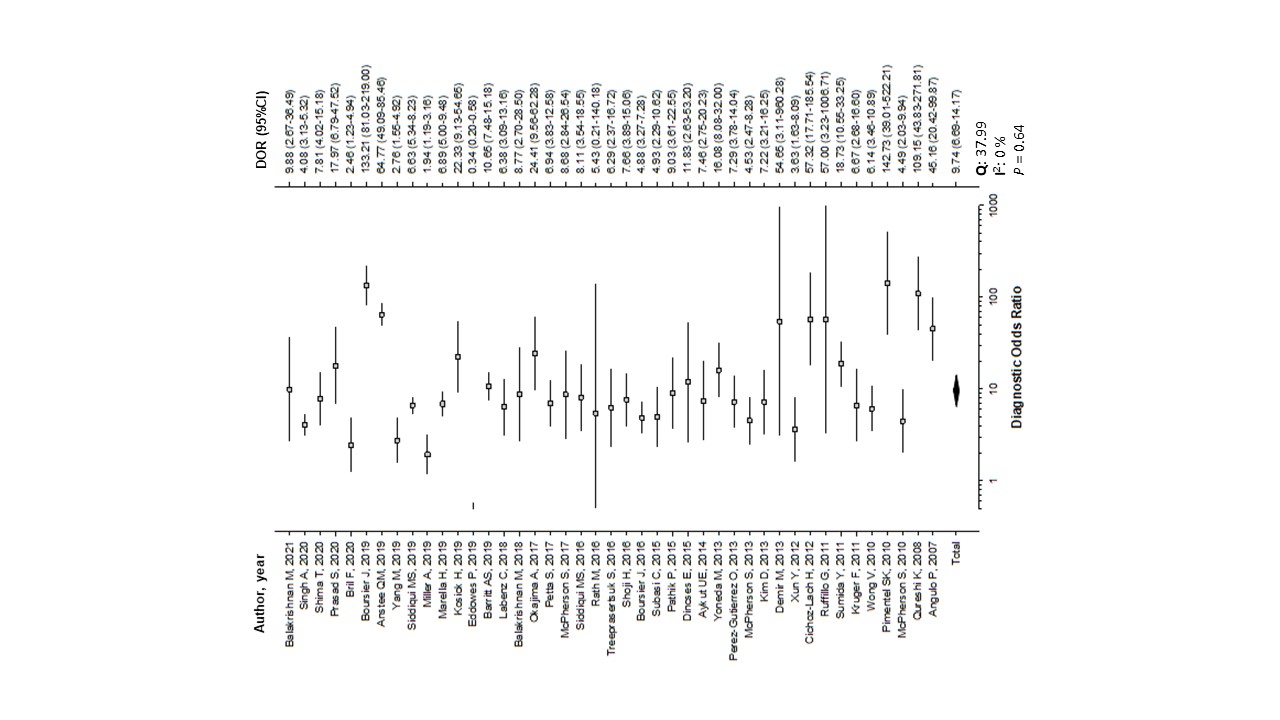
**

# **Supplementary Figure 22.** Forest plot of meta-analysis of diagnostic odds ratio for NFS values in advanced fibrosis.

**
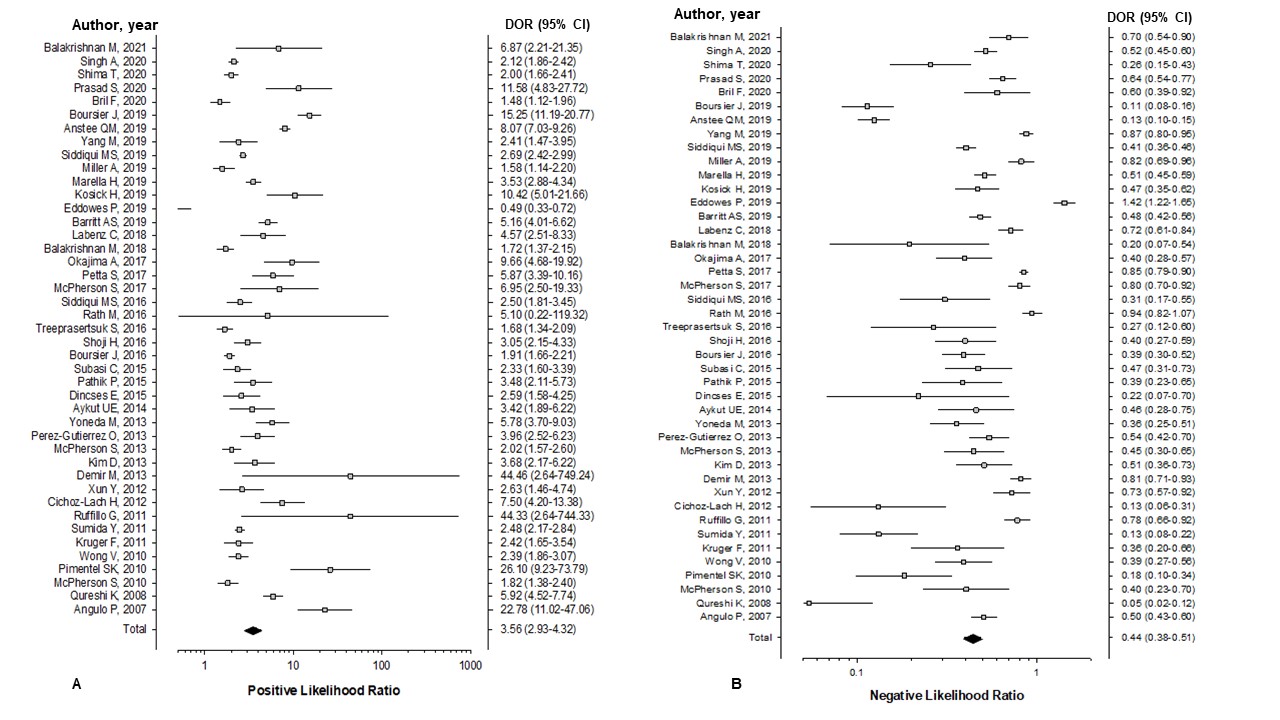
**

# **Supplementary Figure 23.** (A) Forest plot of meta-analysis of positive likelihood ratio and (B) negative likelihood ratio for NFS values in advanced fibrosis.

***Diagnosis of cirrhosis (F0-3 vs F4)***


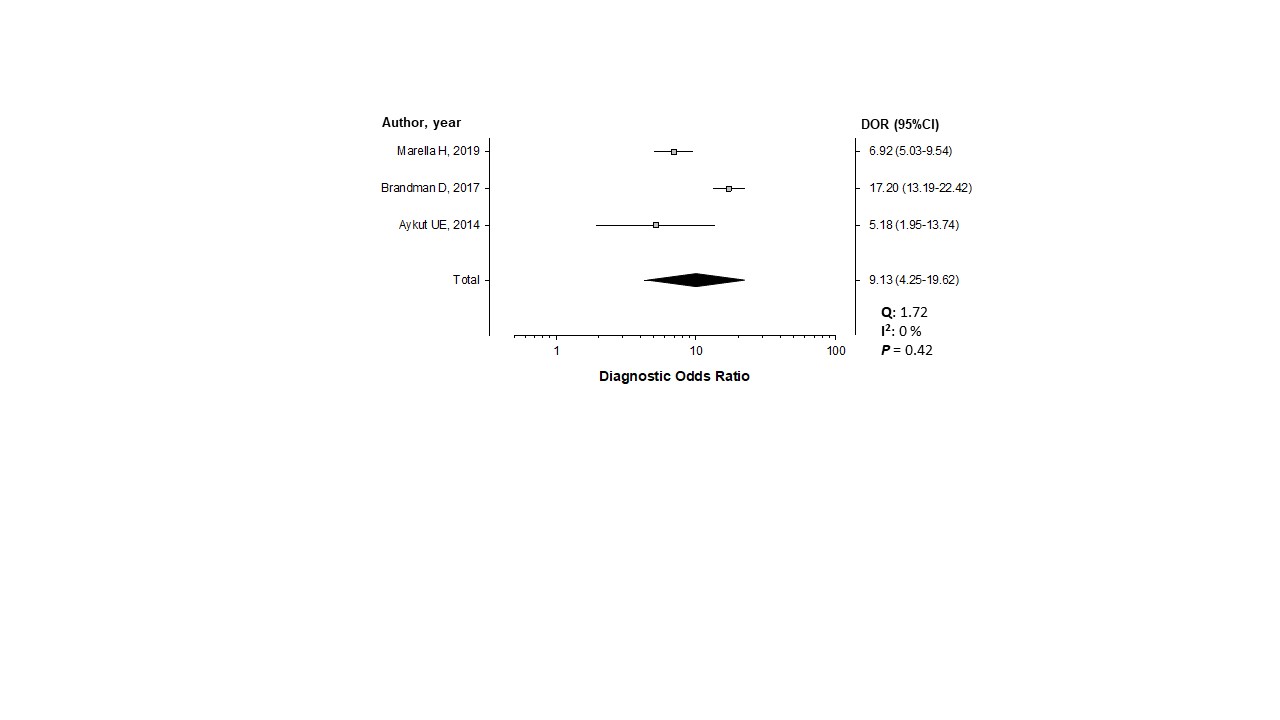


# **Supplementary Figure 24.** Forest plot of meta-analysis of diagnostic odds ratio for NFS values in cirrhosis.


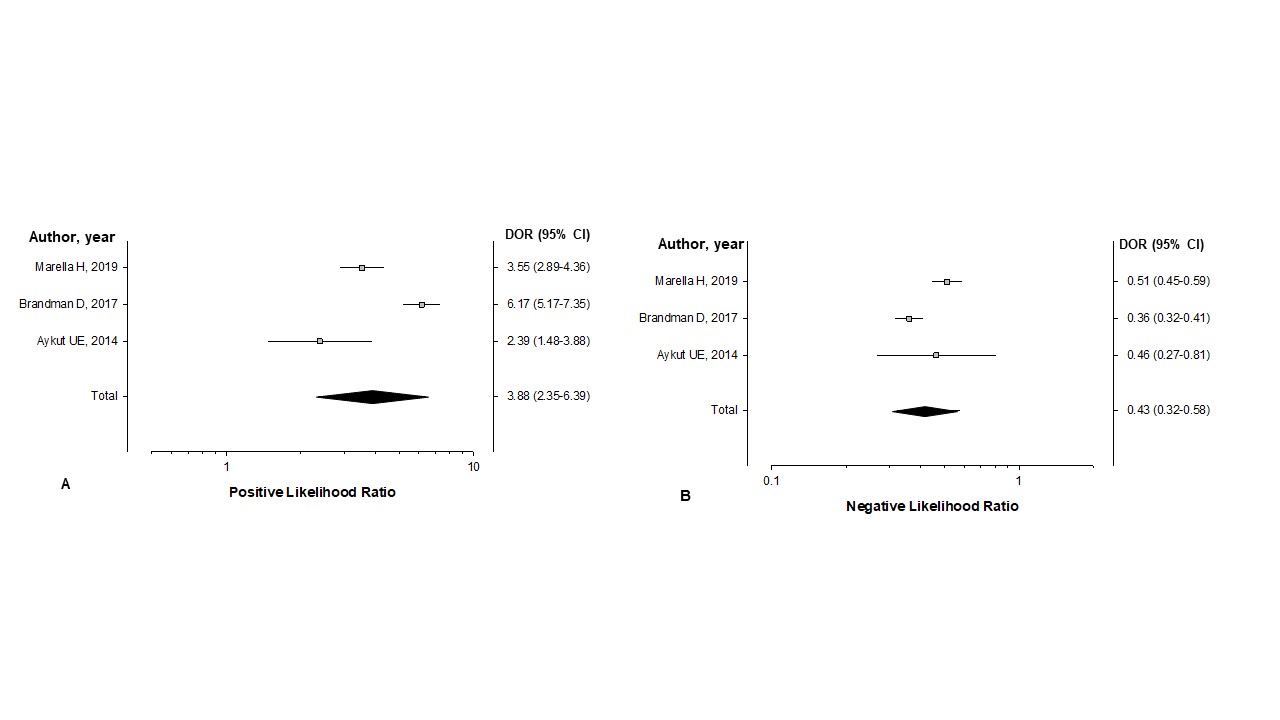


# **Supplementary Figure 25.** (A) Forest plot of meta-analysis of positive likelihood ratio and (B) Forest plot of meta-analysis of negative likelihood ratio for NFS values in cirrhosis.

**BARD score**

***Diagnosis of significant fibrosis (F0-1 vs F2-4)***

**
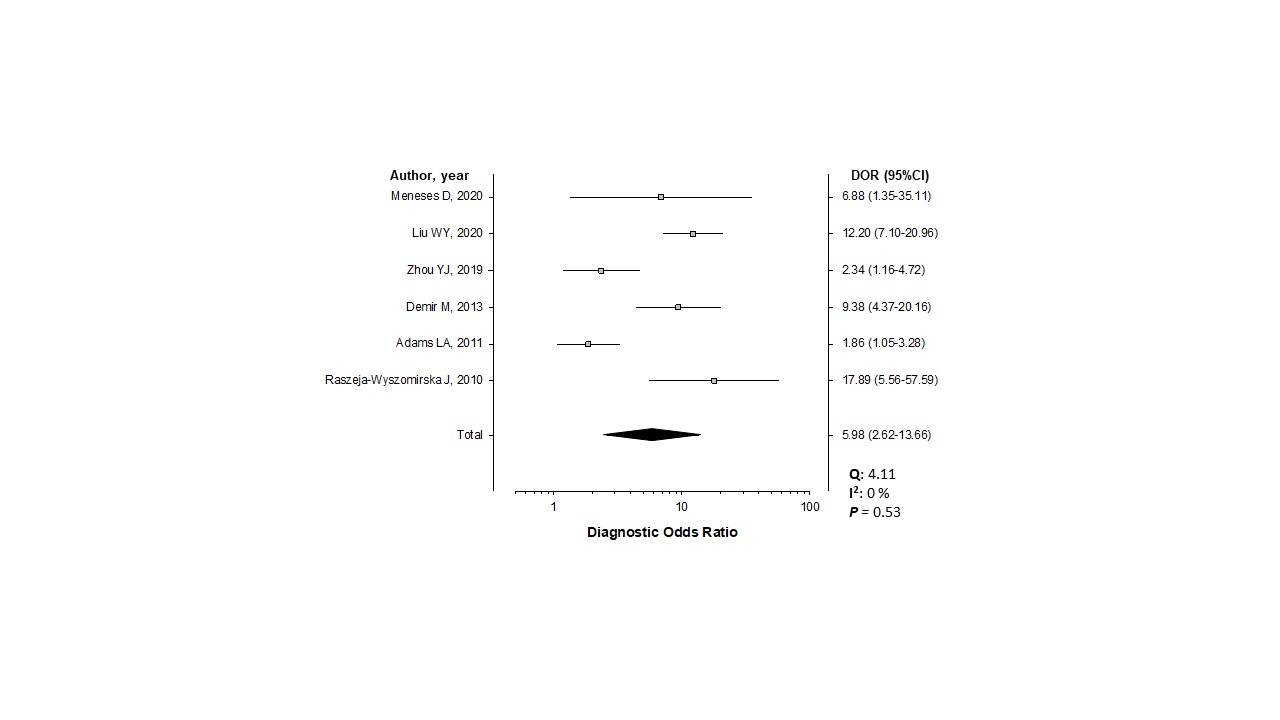
**

**Supplementary Figure 26.** Forest plot of meta-analysis of diagnostic odds ratio for BARD score values in significant fibrosis.

**
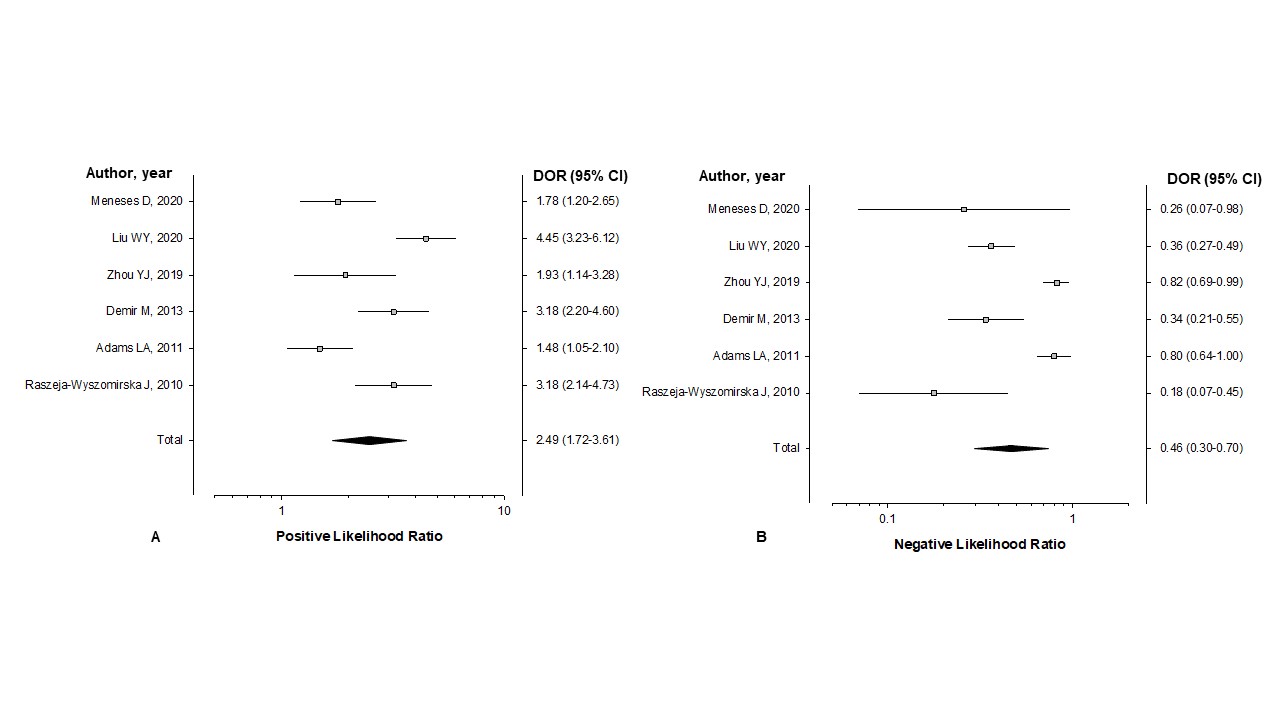
****Supplementary Figure 27.** (A) Forest plot of meta-analysis of positive likelihood ratio and (B) negative likelihood ratio for BARD score values in significant fibrosis.

***Diagnosis of advanced fibrosis (F0-2 vs F3-4)***

**
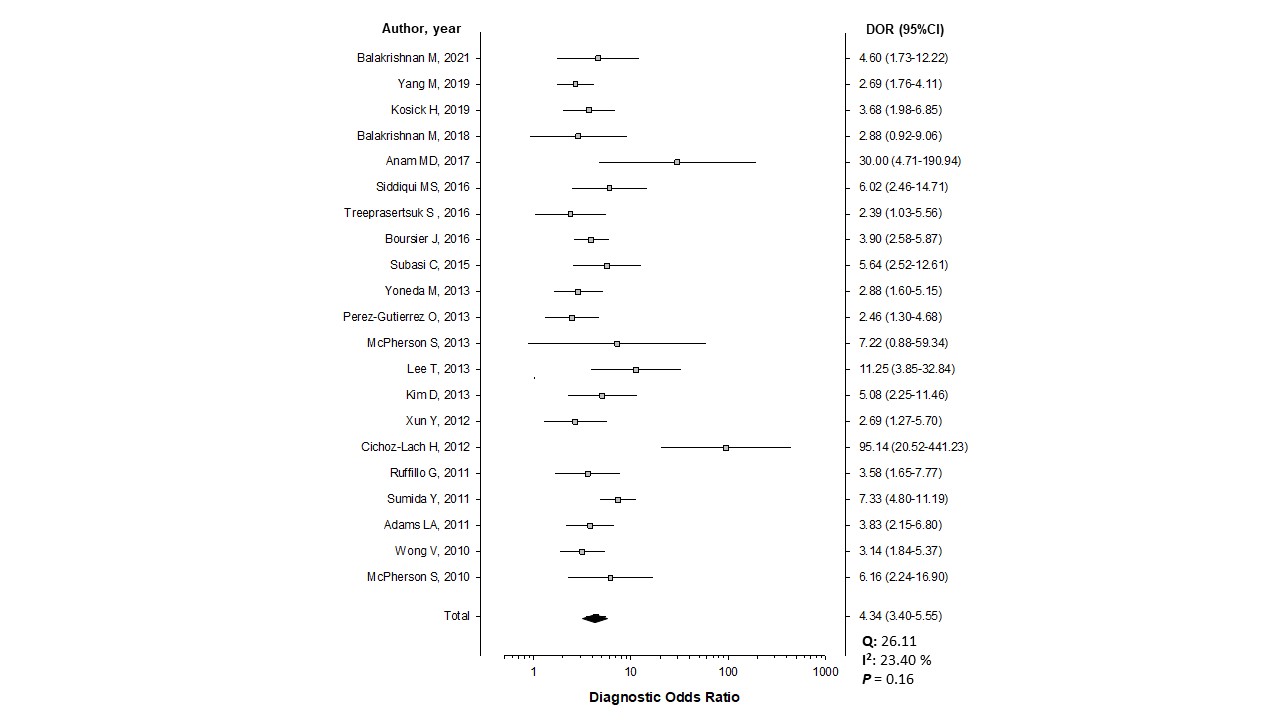
**

# **Supplementary Figure 28.** Forest plot of meta-analysis of diagnostic odds ratio for BARD score values in advanced fibrosis.

**
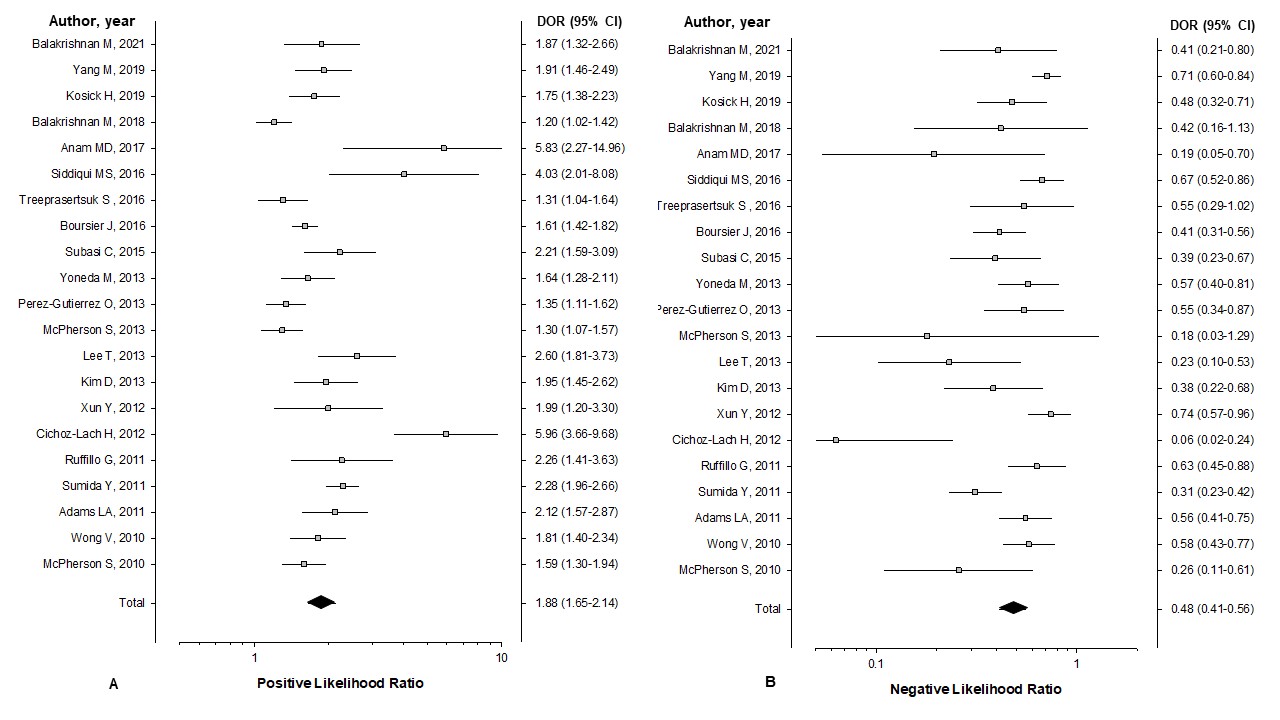
**

# **Supplementary Figure 29.** (A) Forest plot of meta-analysis of positive likelihood ratio and (B) negative likelihood ratio for BARD score values in advanced fibrosis.

**FibroMeter**

***Diagnosis of significant fibrosis (F0-1 vs F2-4)***

**
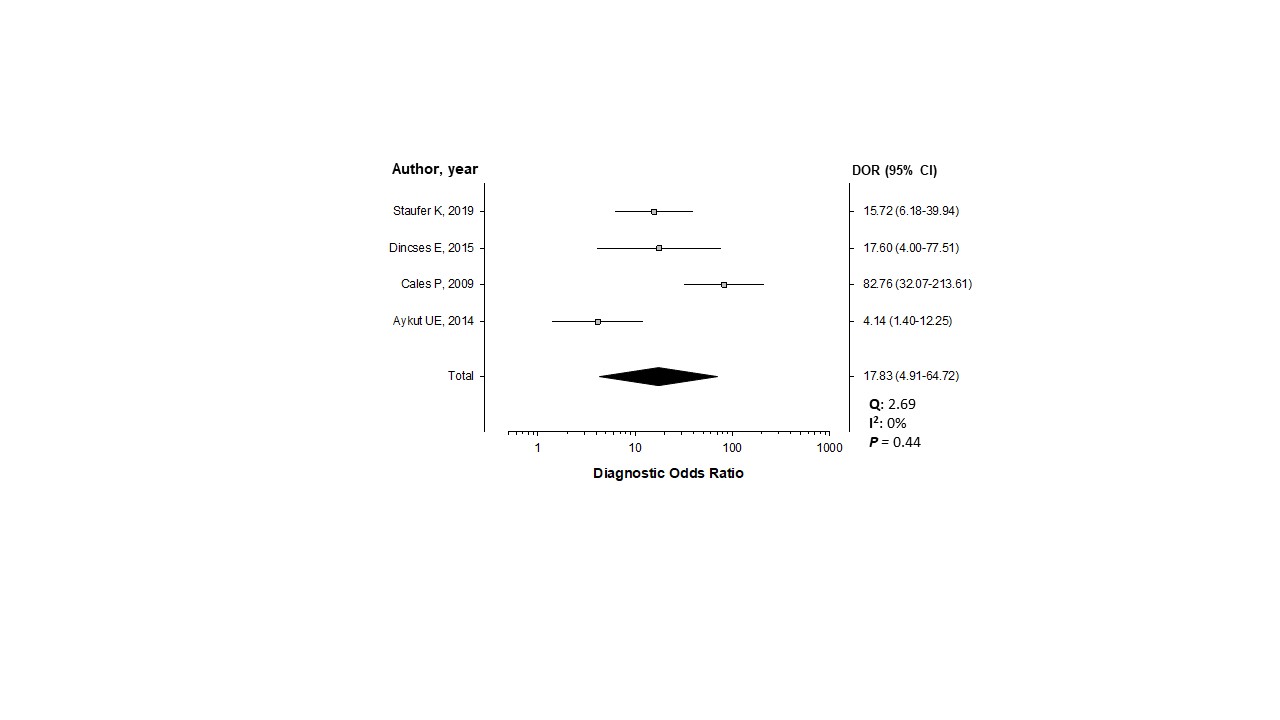
**

# **Supplementary Figure 30.** Forest plot of meta-analysis of diagnostic odds ratio for FibroMeter values in significant fibrosis.

**
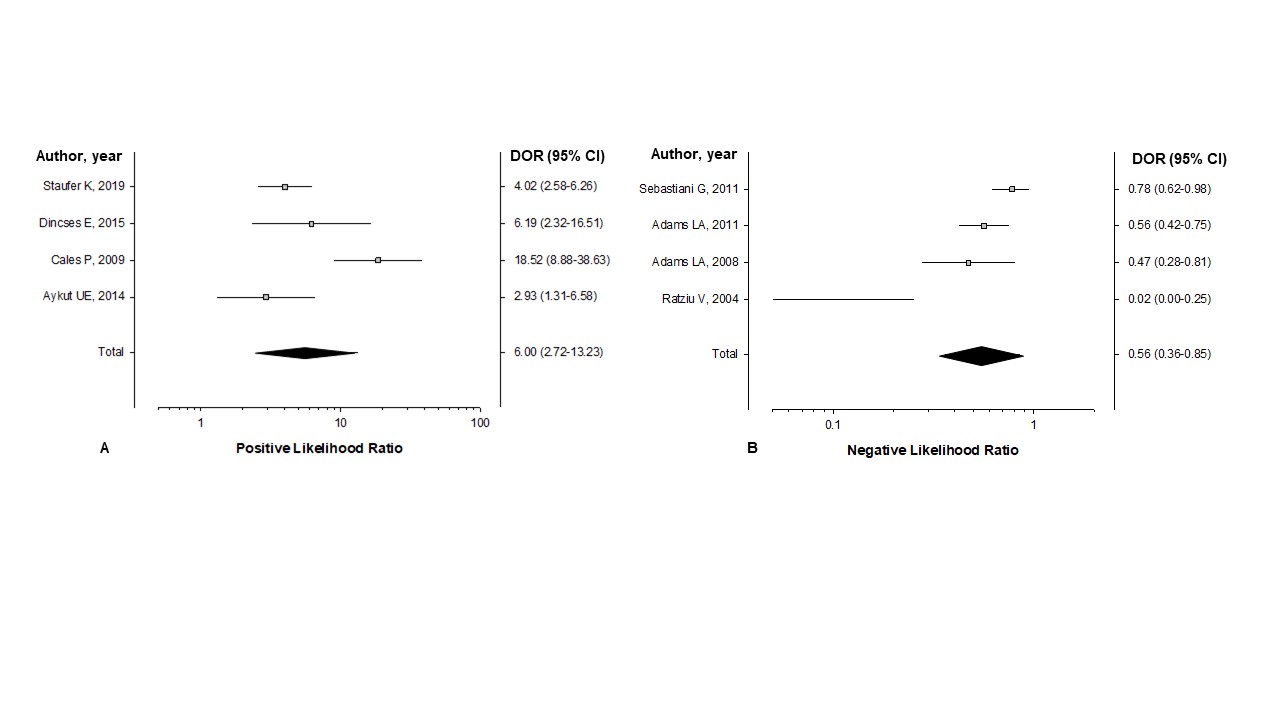
**

# **Supplementary Figure 31.** (A) Forest plot of meta-analysis of positive likelihood and (B) negative likelihood ratio for FibroMeter values in significant fibrosis.

***Diagnosis of advanced fibrosis (F0-2 vs F3-4)***

**
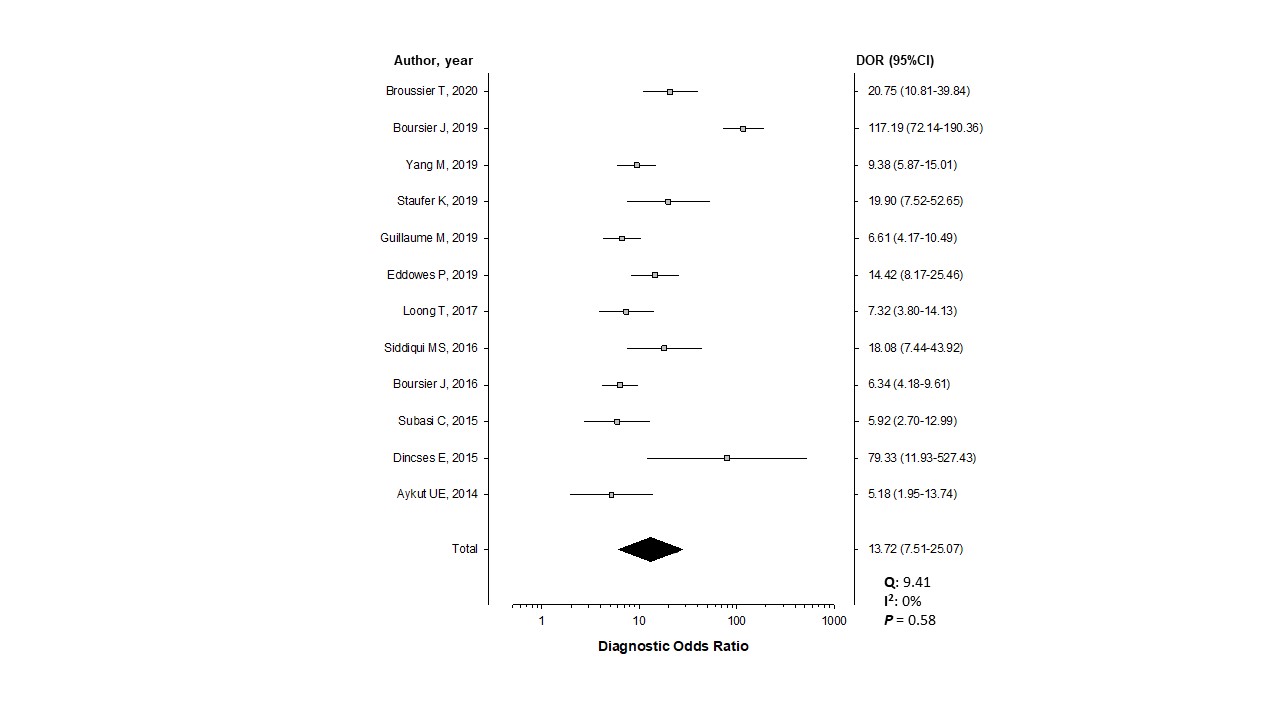
**

# **Supplementary Figure 32.** Forest plot of meta-analysis of diagnostic odds ratio for FibroMeter values in advanced fibrosis.

**
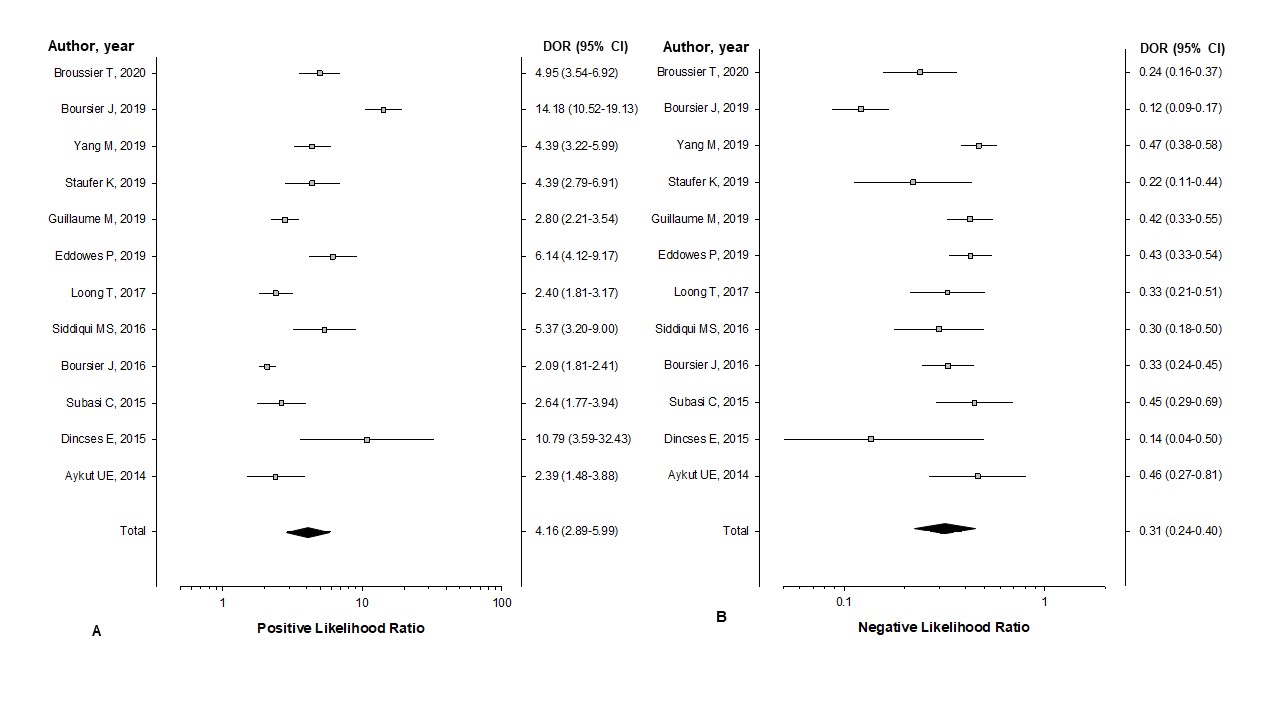
**

# **Supplementary Figure 33.** (A) Forest plot of meta-analysis of positive likelihood ratio and (B) negative likelihood ratio for FibroMeter values in advanced fibrosis.

**FibroTest**

***Diagnosis of significant fibrosis (F0-1 vs F2-4)***

**
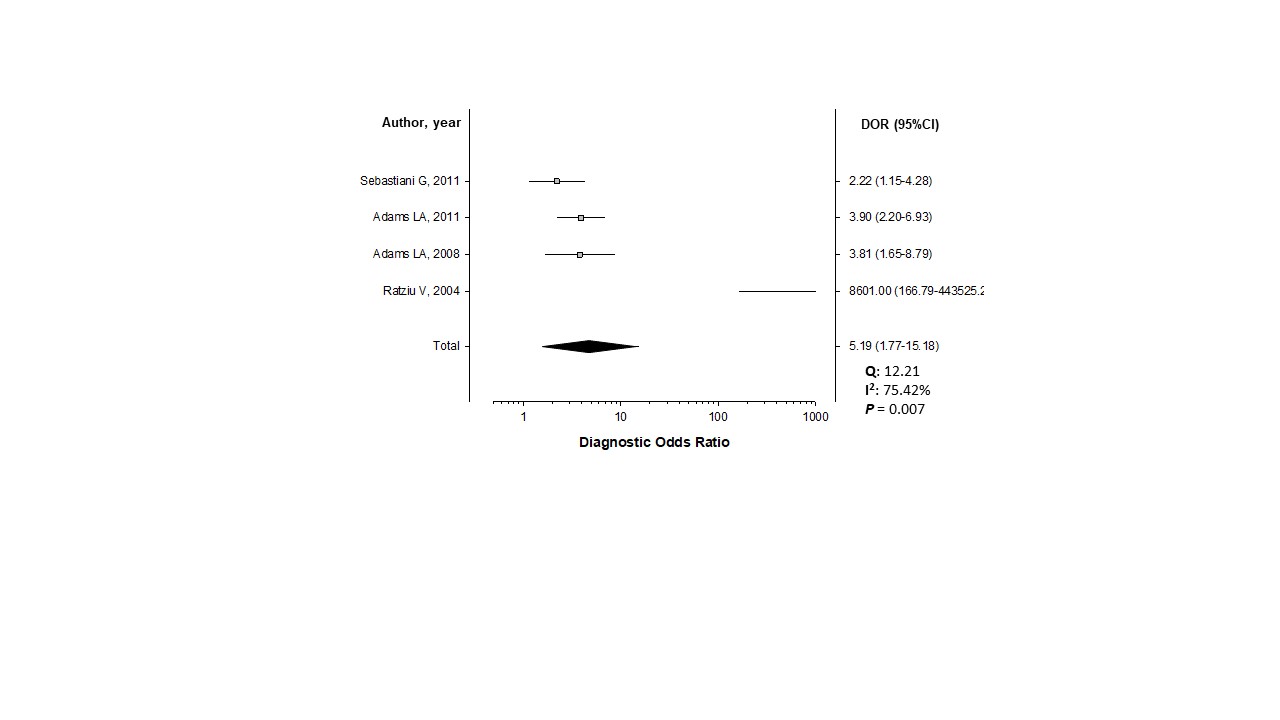
**

# **Supplementary Figure 34.** Forest plot of meta-analysis of diagnostic odds ratio for FibroTest values in significant fibrosis.

**
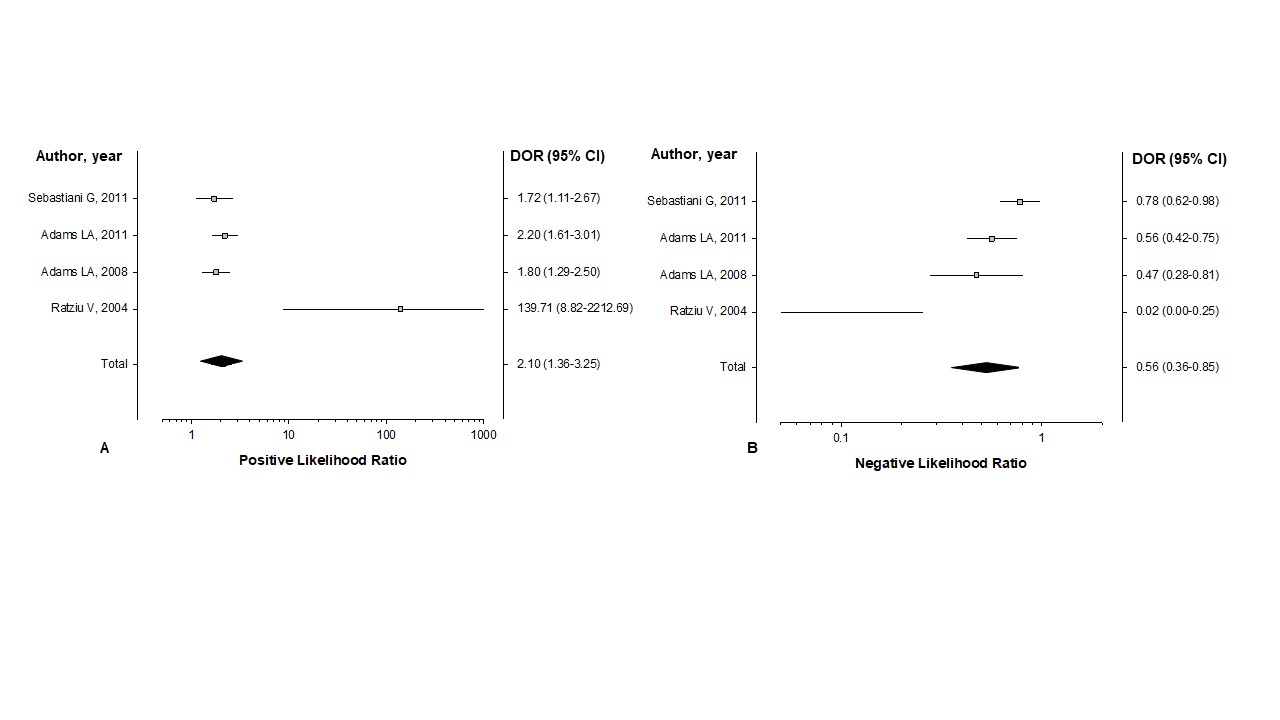
**

# **Supplementary Figure 35.** (A) Forest plot of meta-analysis of positive likelihood ratio and (B) negative likelihood ratio for FibroTest values in significant fibrosis.

***Diagnosis of advanced fibrosis (F0-2 vs F3-4)***

**
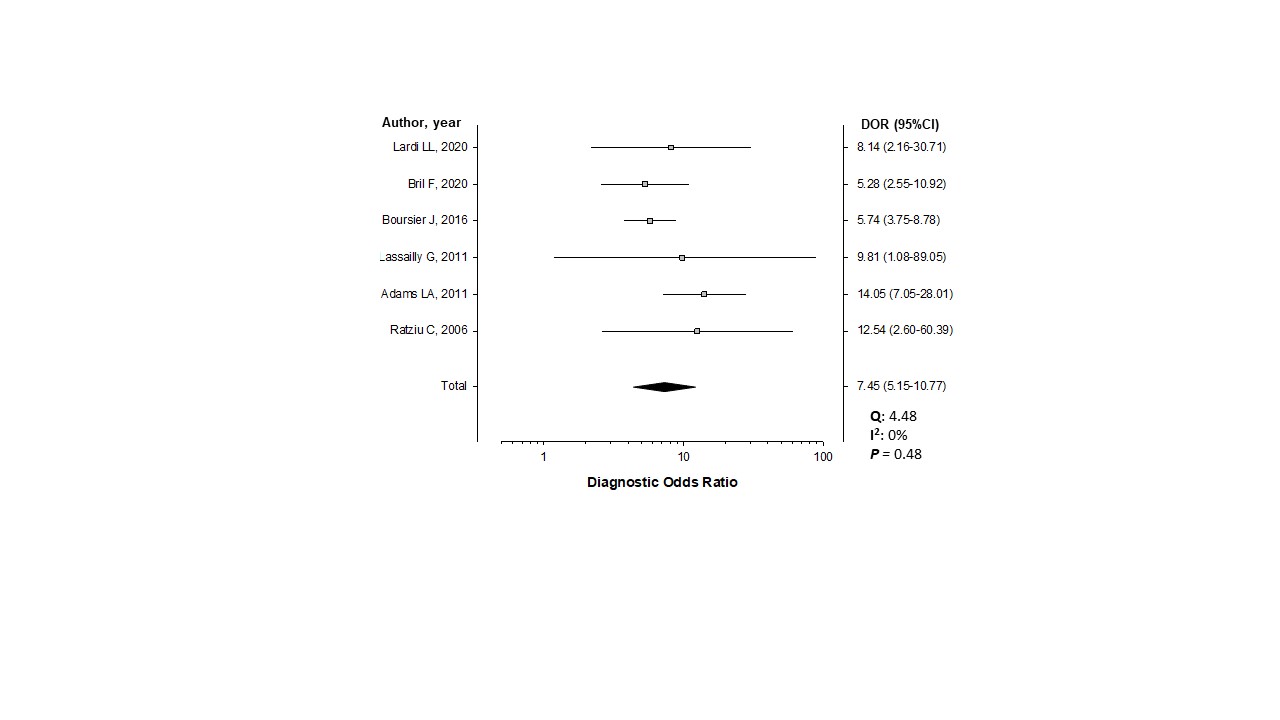
**

# **Supplementary Figure 36.** Forest plot of meta-analysis of diagnostic odds ratio for FibroTest values in advanced fibrosis.

**
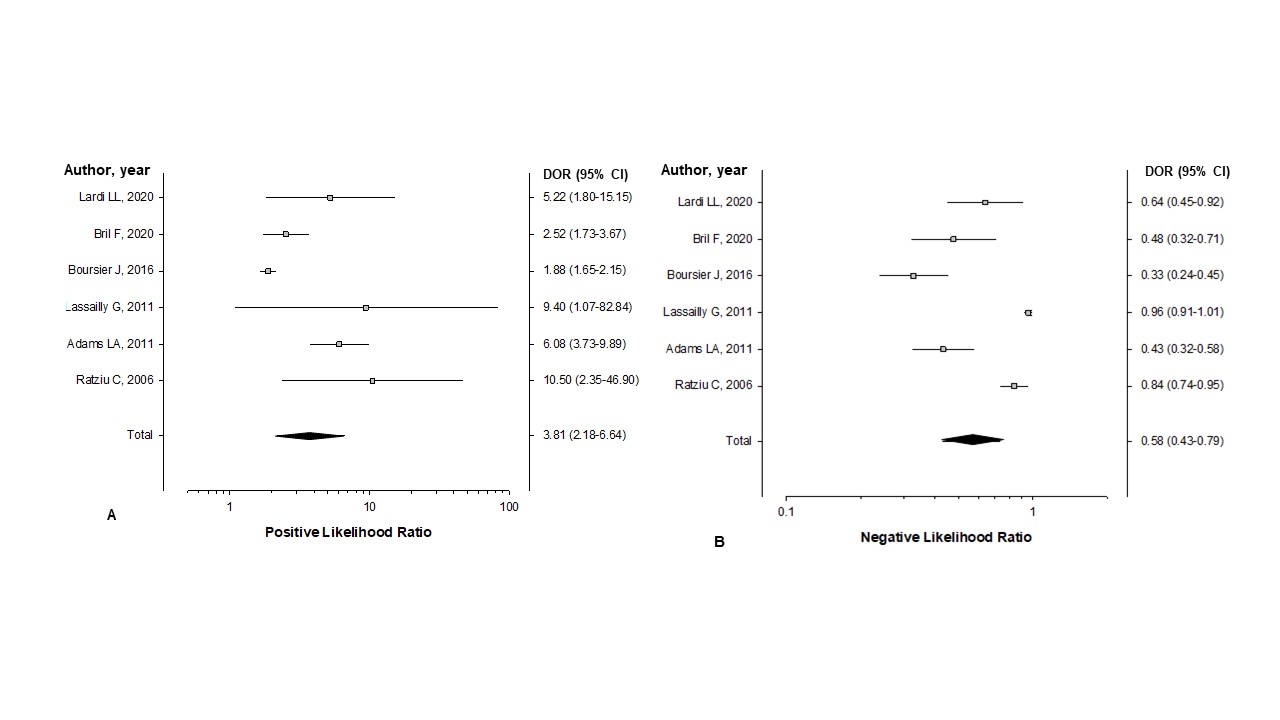
**

# **Supplementary Figure 37.** (A) Forest plot of meta-analysis of positive likelihood ratio and (B) negative likelihood ratio for FibroTest values in advanced fibrosis.

**ELF**

***Diagnosis of advanced fibrosis (F0-2 vs F3-4)***

**
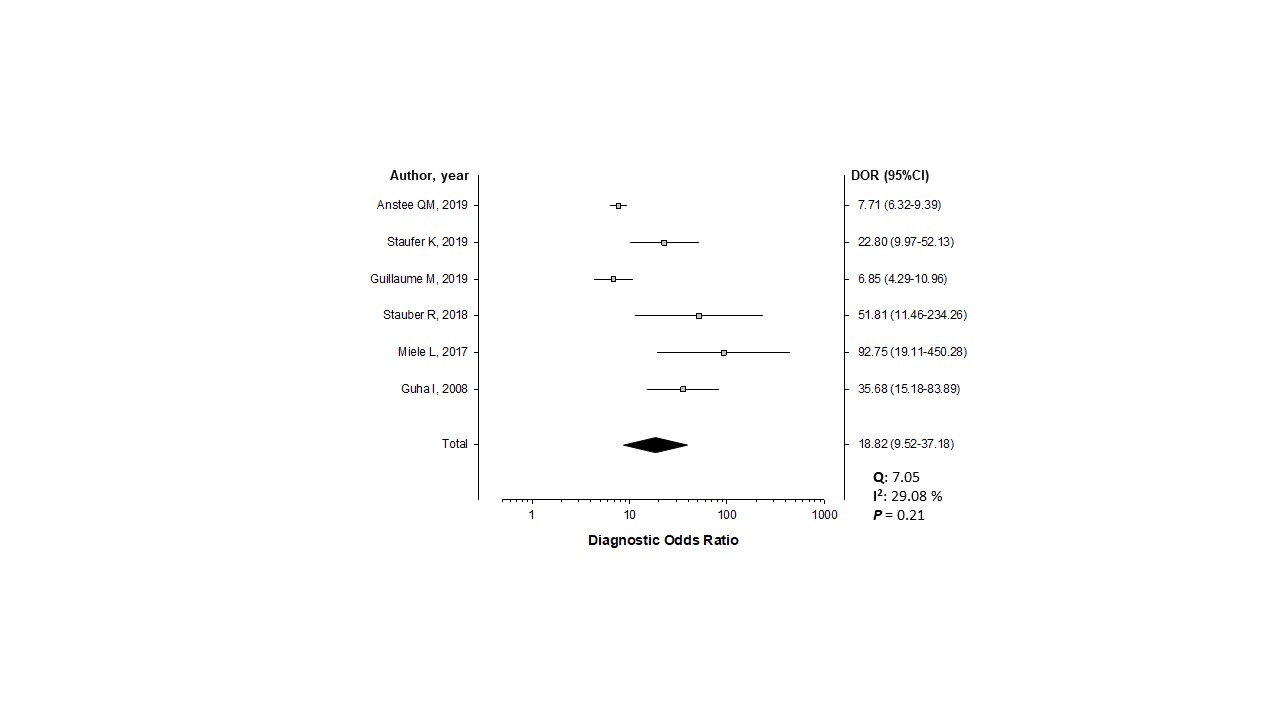
**

# **Supplementary Figure 38.** Forest plot of meta-analysis of diagnostic odds ratio for ELF values in advanced fibrosis.

**
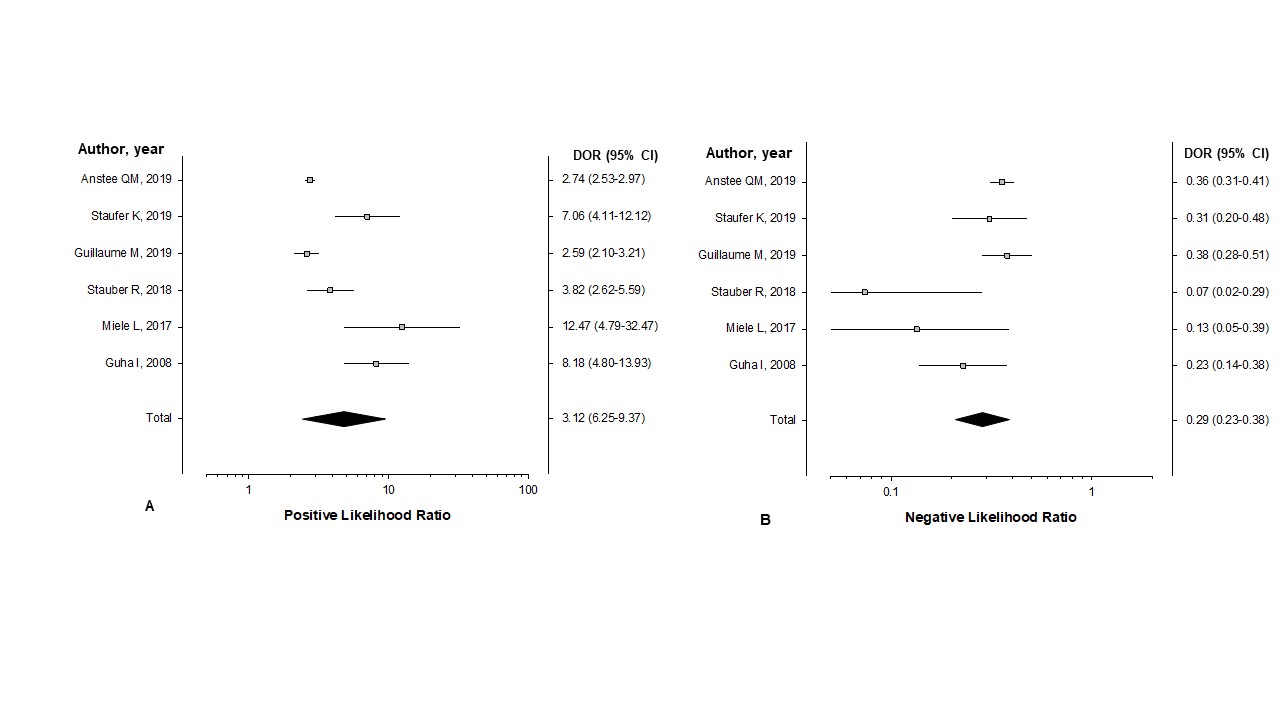
**

# **Supplementary Figure 39.** (A) Forest plot of meta-analysis of positive likelihood ratio and (B) negative likelihood ratio for ELF values in advanced fibrosis.

| **Supplementary Table 7.** Comparison of sAUC, sensitivity and specificity for serological biomarkers for in predicting liver fibrosis severity in people with MASLD. | | | | |
| --- | --- | --- | --- | --- |
| **Models** | **No. of studies/**  **patient*** | **AUC** | **Sensitivity rate %**  **(95% CI)** | **Specificity rate %**  **(95% CI)** |
| **APRI** | | | | |
| Any fibrosis | 3 (1535) | 0.76 | 77 (61-88) | 64 (48-78) |
| Significant fibrosis | 14 (4845) | 0.76 | 63 (53-72) | 79 (69-86) |
| Advanced fibrosis | 33 (10341) | 0.78 | 60 (50-69) | 82 (76-87) |
| Cirrhosis | 3 (2632) | 0.72 | 47 (3-84) | 87 (50-98) |
| **FIB-4** | | | | |
| Any fibrosis | 5 (2172) | 0.77 | 77 (61- 87) | 68 (57-78) |
| Significant fibrosis | 15 (5222) | 0.75 | 64 (52-74) | 76 (66-84) |
| Advanced fibrosis | 43 (16519) | 0.81 | 60 (52-68) | 87 (82-91) |
| Cirrhosis | 4 (1886) | 0.83 | 69 (43-86) | 87 (57-97) |
| **NFS** | | | | |
| Any fibrosis | 5 (2725) | 0.71 | 66 (62-70) | 73 (64-81) |
| Significant fibrosis | 14 (3031) | 0.81 | 69 (56-79) | 80 (71-88) |
| Advanced fibrosis | 43 (17946) | 0.81 | 62 (53-70) | 85 (79-90) |
| Cirrhosis | 3 (2478) | 0.69 | 63 (58-68) | 84 (73-91) |
| **BARD score** | | | | |
| Significant fibrosis | 6 (1275) | 0.77 | 66 (45-82) | 75 (65-83) |
| Advanced fibrosis | 21 (4911) | 0.73 | 72 (64-79) | 63 (54-71) |
| **FibroMeter** | | | | |
| Significant fibrosis | 4 (651) | 0.88 | 68 (48-82) | 89 (80-95) |
| Advanced fibrosis | 12 (3863) | 0.84 | 74 (68-79) | 82 (76-87) |
| **FibroTest** | | | | |
| Significant fibrosis | 4 (640) | 0.86 | 72 (28-94) | 85 (45-98) |
| Advanced fibrosis | 6 (1620) | 0.78 | 40 (15-72) | 93 (73-99) |
| **ELF** | | | | |
| Advanced fibrosis | 6 (4200) | 0.87 | 79 (68-87) | 84 (75-90) |

*APRI aspartate aminotransferase to platelet ratio index; 95% CI confidence interval; ELF enhanced liver fibrosis; FIB-4 fibrosis index-4; MASLD metabolic dysfunction-associated steatotic liver disease; NFS Non-alcoholic fatty liver disease fibrosis score; sAUC summary area under curve.*

#


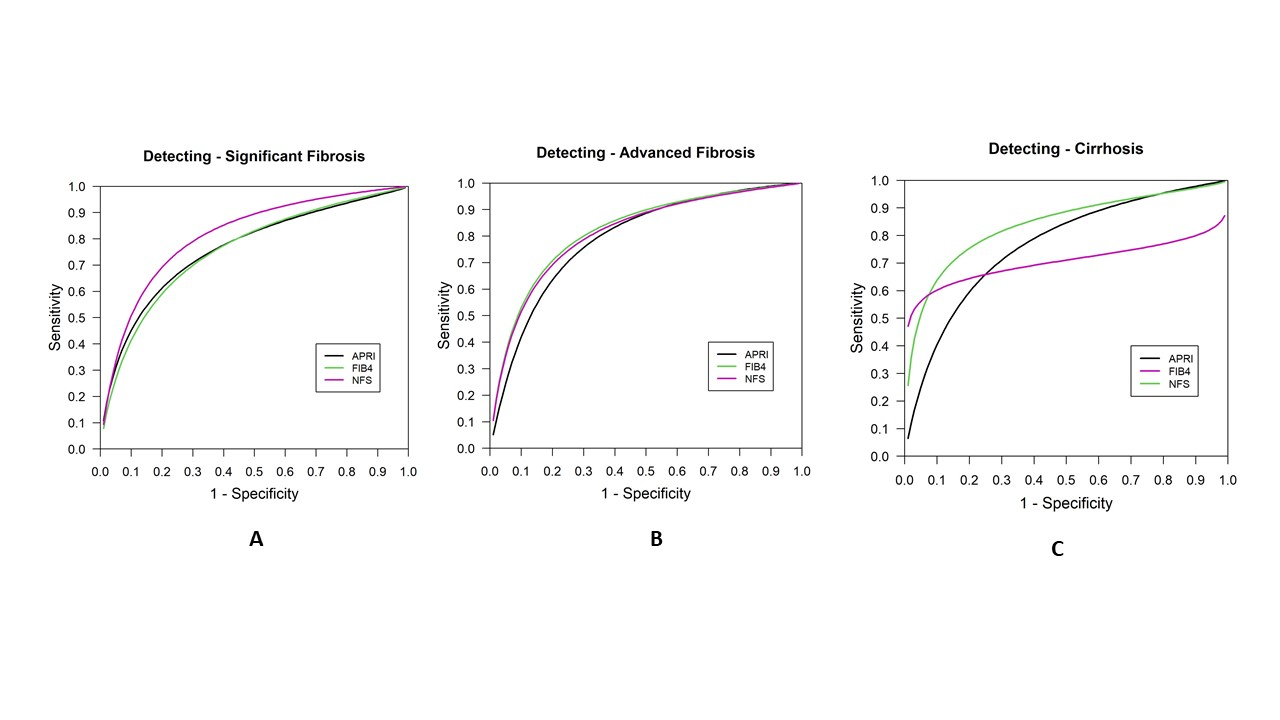


# **Supplementary Figure 40.** Sensitivity analysis with the most frequently used serological biomarkers and severities. (A) APRI, FIB-4, and NFS in detecting significant fibrosis, (B) APRI, FIB-4, and NFS in detecting advanced fibrosis, and (C) APRI, FIB-4, and NFS in detecting cirrhosis.

| **Supplementary Table 8.** Results of the meta-analysis of the most frequent serological biomarkers used in predicting liver fibrosis severity in people with MASLD. | | | | | | | | | | |
| --- | --- | --- | --- | --- | --- | --- | --- | --- | --- | --- |
| **Serological biomarkers** | **No. of studies/**  **patient*** | **AUC** | **Sen rate %**  **(95% CI)** | **Spe rate %**  **(95% CI)** | **DOR**  **(95% CI)** | **Cochran’s Q** | ***p*** | **I^2^** | **LR+ (95% CI)** | **LR-**  **(95% CI)** |
| **APRI** | | | | |  |  |  |  |  |  |
| Significant fibrosis | 14 (4845) | 0.76 | 63 (53-72) | 79 (69-86) | 6.29 (4.47-8.92) | 16.13 | 0.24 | 19.4 | 2.69 (2.23-3.23) | 0.48 (0.40-0.58) |
| Advanced fibrosis | 33 (10341) | 0.78 | 60 (50-69) | 82 (76-87) | 6.45 (4.83-8.60) | 42.78 | 0.009 | 25.21 | 2.96 (2.49-3.52) | 0.50 (0.43-0.57) |
| Cirrhosis | 3 (2632) | 0.72 | 47 (3-84) | 87 (50-98) | 6.21 (4.34-8.89) | 1.71 | 0.42 | 0 | 3.11 (2.15-4.50) | 0.53 (0.31-0.89) |
| **FIB-4** | | | | |  |  |  |  |  |  |
| Significant fibrosis | 15 (5222) | 0.75 | 64 (52-74) | 76 (66-84) | 5.75 (4.11-8.05) | 18.26 | 0.19 | 23.33 | 2.51 (2.07-3.05) | 0.50 (0.43-0.59) |
| Advanced fibrosis | 43 (16519) | 0.81 | 60 (52-68) | 87 (82-91) | 10.43 (7.25-15.02) | 33.1 | 0.83 | 0 | 4.09 (3.33-5.02) | 0.45 (0.39-0.52) |
| Cirrhosis | 4 (1886) | 0.83 | 69 (43-86) | 87 (57-97) | 14.95 (9.96-22.44) | 4.16 | 0.24 | 27.88 | 4.66 (2.41-9.02) | 0.38 (0.19-0.78) |
| **NFS** | | | | |  |  |  |  |  |  |
| Significant fibrosis | 14 (3031) | 0.81 | 69 (56-79) | 80 (71-88) | 9.45 (5.17-17.5) | 13.53 | 0.40 | 3.91 | 3.35 (2.42-4.63) | 0.42 (0.33-0.54) |
| Advanced fibrosis | 43 (17946) | 0.81 | 62 (53-70) | 85 (79-90) | 9.74 (6.69-14.17) | 37.99 | 0.64 | 0 | 3.56 (2.93-4.32) | 0.44 (0.38-0.51) |
| Cirrhosis | 3 (2478) | 0.69 | 63 (58-68) | 84 (73-91) | 9.13 (4.25-19.62) | 1.72 | 0.42 | 0 | 3.88 (2.35-6.39) | 0.43 (0.32-0.58) |

*APRI aspartate aminotransferase to platelet ratio index; 95% CI confidence interval; ELF enhanced liver fibrosis; FIB-4 fibrosis index-4; NFS Non-alcoholic fatty liver disease fibrosis score; AUC, area under curve, Sen sensitivity Spe specificity, MASLD* *metabolic dysfunction-associated steatotic liver disease, I^2^ heterogeneity; LR+ positive likelihood ratio; LR- negative likelihood ratio, p statistically significant value.*

|  |  |
| --- | --- |
|  |  |
|  |  |
|  |  |

# **Supplementary reference**

1. Ratziu V, Le Calves S, Messous D, Charlotte F, Bonhay L, Munteanu M, Poynard T. Diagnostic value of biochemical markers (FIBROTEST) for the prediction of liver fibrosis in patients with non-alcoholic fatty liver disease (NAFLD). (2004) 84:100.

2. Amernia B, Moosavy SH, Banookh F, Zoghi G. FIB-4, APRI, and AST/ALT ratio compared to FibroScan for the assessment of hepatic fibrosis in patients with non-alcoholic fatty liver disease in Bandar Abbas, Iran. *BMC Gastroenterol* (2021) 21:453. doi: 10.1186/s12876-021-02038-3

3. Balakrishnan M, Seth A, Cortes-Santiago N, Jain S, Sood GK, El-Serag HB, Thrift AP. External Validation of Four Point-of-Care Noninvasive Scores for Predicting Advanced Hepatic Fibrosis in a Predominantly Hispanic NAFLD Population. *Dig Dis Sci* (2021) 66:2387–2393. doi: 10.1007/s10620-020-06501-1

4. Younes R, Caviglia GP, Govaere O, Rosso C, Armandi A, Sanavia T, Pennisi G, Liguori A, Francione P, Gallego-Durán R, et al. Long-term outcomes and predictive ability of non-invasive scoring systems in patients with non-alcoholic fatty liver disease. *J Hepatol* (2021) 75:786–794. doi: 10.1016/j.jhep.2021.05.008

5. Calès P, Lainé F, Boursier J, Deugnier Y, Moal V, Oberti F, Hunault G, Rousselet MC, Hubert I, Laafi J, et al. Comparison of blood tests for liver fibrosis specific or not to NAFLD. *J Hepatol* (2009) 50:165–173. doi: 10.1016/j.jhep.2008.07.035

6. Fujii H, Enomoto M, Fukushima W, Ohfuji S, Mori M, Kobayashi S, Iwai S, Morikawa H, Tamori A, Sakaguchi H, et al. Noninvasive laboratory tests proposed for predicting cirrhosis in patients with chronic hepatitis C are also useful in patients with non-alcoholic steatohepatitis. *J Gastroenterol* (2009) 44:608–614. doi: 10.1007/s00535-009-0046-6

7. Shah AG, Lydecker A, Murray K, Tetri BN, Contos MJ, Sanyal AJ, Abrams S, Arceo D, Espinosa D, Fairly L, et al. Comparison of noninvasive markers of fibrosis in patients with nonalcoholic fatty liver disease. *Clin Gastroenterol Hepatol* (2009) 7:1104–1112. doi: 10.1016/j.cgh.2009.05.033

8. Calès P, Boursier J, Chaigneau J, Lainé F, Sandrini J, Michalak S, Hubert I, Dib N, Oberti F, Bertrais S, et al. Diagnosis of different liver fibrosis characteristics by blood tests in non-alcoholic fatty liver disease. *Liver Int* (2010) 30:1346–1354. doi: 10.1111/j.1478-3231.2010.02314.x

9. McPherson S, Stewart SF, Henderson E, Burt AD, Day CP. Simple non-invasive fibrosis scoring systems can reliably exclude advanced fibrosis in patients with non-alcoholic fatty liver disease. *Gut* (2010) 59:1265–1269. doi: 10.1136/gut.2010.216077

10. Balakrishnan M, Gaba R, Jain S, Thrift AP. Clinical Fibrosis Prediction Scores Perform Poorly Among Mexican/Central American Patients with NAFLD. *Hepatology* (2018) 68:184–1353. doi: 10.1002/hep.30257

11. Barrit AS, Lok AS, Reddy KR, Weiss LM, Firpi RJ, Thuluvath PJ, Trihn HN, Djedjos S, Haubrich R, Billin A, et al. Routinely avaliable noninvasive tests performs well in identifying patients with advanced fibrosis due to NASH: data from the target-nash observational cohort. *Hepatology* (2019) 70:188–1382. doi: 10.1002/hep.30941

12. Pimentel SK, Strobel R, Gonçalves CG, Sakamoto DG, Ivano FH, Coelho CU. Evaluation of the nonalcoholic fat liver diasease fibrosis score for patients undergoing bariatric surgery. *Arq Gastroenterol* (2010) 47:170–173.

13. Raszeja-Wyszomirska J, Szymanik B, Ławniczak M, Kajor M, Chwist A, Milkiewicz P, Hartleb M. Validation of the BARD scoring system in Polish patients with nonalcoholic fatty liver disease (NAFLD). *BMC Gastroenterol* (2010) 10: doi: 10.1186/1471-230X-10-67

14. Wong VWS, Vergniol J, Wong GLH, Foucher J, Chan HLY, Le Bail B, Choi PCL, Kowo M, Chan AWH, Merrouche W, et al. Diagnosis of fibrosis and cirrhosis using liver stiffness measurement in nonalcoholic fatty liver disease. *Hepatology* (2010) 51:454–462. doi: 10.1002/hep.23312

15. Miller M, Jafferbhoy H, Walsh S, Dillon JF. Performance of Simple Algorithm Tests for the Detection of Fibrosis in Nalfd. *J Hepatol* (2010) 52:S59–S182. doi: 10.1016/s0168-8278(10)60363-7

16. Adams LA, George J, Bugianesi E, Rossi E, De Boer WB, van der Poorten D, Ching HLI, Bulsara M, Jeffrey GP. Complex non-invasive fibrosis models are more accurate than simple models in non-alcoholic fatty liver disease. *J Gastroenterol Hepatol* (2011) 26:1536–1543. doi: 10.1111/j.1440-1746.2011.06774.x

17. Lassailly G, Caiazzo R, Hollebecque A, Buob D, Leteurtre E, Arnalsteen L, Louvet A, Pigeyre M, Raverdy V, Verkindt H, et al. Validation of noninvasive biomarkers (FibroTest, SteatoTest, and NashTest) for prediction of liver injury in patients with morbid obesity. *Eur J Gastroenterol Hepatol* (2011) 23:499–506. doi: 10.1097/MEG.0b013e3283464111

18. Ruffillo G, Fassio E, Alvarez E, Landeira G, Longo C, Domínguez N, Gualano G. Comparison of NAFLD fibrosis score and BARD score in predicting fibrosis in nonalcoholic fatty liver disease. *J Hepatol* (2011) 54:160–163. doi: 10.1016/j.jhep.2010.06.028

19. Sebastiani G, Castera L, Halfon P, Pol S, Mangia A, Di Marco V, Pirisi M, Voiculescu M, Bourliere M, Alberti A. The impact of liver disease aetiology and the stages of hepatic fibrosis on the performance of non-invasive fibrosis biomarkers: An international study of 2411 cases. *Aliment Pharmacol Ther* (2011) 34:1202–1216. doi: 10.1111/j.1365-2036.2011.04861.x

20. Sumida Y, Yoneda M, Hyogo H, Itoh Y, Ono M, Fujii H, Eguchi Y, Suzuki Y, Aoki N, Kanemasa K, et al. Validation of the FIB4 index in a Japanese nonalcoholic fatty liver disease population. *BMC Gastroenterol* (2012) 12: doi: 10.1186/1471-230X-12-2

21. Cebreiros IL, Guzmán FA, Velasco J, Ruiz CR, Villanueva MM, Elízaga I de M, Ruiz AM, Bernal MF, Mompeán JL, Bernal AB. Clinical usefulness of ELF index in the assessment of non alcoholic fatty liver disease. *Clin Chem Lab Med* (2014) 52:205–379. doi: 10.1515/cclm-2014-0890

22. Cichoż-Lach H, Celiński K, Prozorow-Król B, Swatek J, Słomka M, Lach T. The BARD score and the NAFLD fibrosis score in the assessment of advanced liver fibrosis in nonalcoholic fatty liver disease. *Med Sci Monit* (2012) 18:CR735–CR740. doi: 10.12659/MSM.883601

23. Francque SMA, Verrijken A, Mertens I, Hubens G, Van Marck E, Pelckmans P, Michielsen P, Van Gaal L. Noninvasive Assessment of Nonalcoholic Fatty Liver Disease in Obese or Overweight Patients. *Clin Gastroenterol Hepatol* (2012) 10:1162–1168. doi: 10.1016/j.cgh.2012.06.019

24. Demir M, Lang S, Schulte S, Quasdorff M, Drebber U, Hardt A, Töx U, Steffen H-M. Prediction of Fibrosis in Nafld – Comparison of Different Scoring Systems Using Routine Laboratory Parameters. *J Hepatol* (2011) 54:S332. doi: 10.1016/s0168-8278(11)60831-3

25. Xun YH, Fan JG, Zang GQ, Liu H, Jiang YM, Xiang J, Huang Q, Shi JP. Suboptimal performance of simple noninvasive tests for advanced fibrosis in Chinese patients with nonalcoholic fatty liver disease. *J Dig Dis* (2012) 13:588–595. doi: 10.1111/j.1751-2980.2012.00631.x

26. Ratziu V, Massard J, Charlotte F, Messous D, Imbert-Bismut F, Bonyhay L, Tahiri M, Munteanu M, Thabut D, Cadranel JF, et al. Diagnostic value of biochemical markers (Fibro Test-FibroSURE) for the prediction of liver fibrosis in patients with non-alcoholic fatty liver disease. *BMC Gastroenterol* (2006) 6:1–13. doi: 10.1186/1471-230X-6-6

27. Chowdhury SD, Ramakrishna B, Eapen E, Goel A, Zachariah UG, Pugazendhi S, Siddartha B, Kurian G. Fibrosis in non-alcoholic fatty liver disease: correlation with simple blood indices and association with tumor necrosis factor-alpha polymorphisms. *Trop Gastroenterol* (2013) 34:31–35.

28. Demir M, Lang S, Nierhoff D, Drebber U, Hardt A, Wedemeyer I, Schulte S, Quasdorff M, Goeser T, Töx U, et al. Stepwise Combination of Simple Noninvasive Fibrosis Scoring Systems Increases Diagnostic Accuracy in Nonalcoholic Fatty Liver Disease. *J Clin Gastroenterol* (2013) 47:719–726. doi: 10.1097/MCG.0b013e3182819a89

29. Lee TH, Han SH, Yang JD, Kim D, Ahmed M. Prediction of Advanced Fibrosis in Nonalcoholic Fatty Liver Disease: An Enhanced Model of BARD Score. *Gut Liver* (2013) 7:323–328. doi: 10.5009/gnl.2014.8.2.228

30. Mahadeva S, Mahfudz AS, Vijayanathan A, Goh KL, Kulenthran A, Cheah PL. Performance of transient elastography (TE) and factors associated with discordance in non-alcoholic fatty liver disease. *J Dig Dis* (2013) 14:604–610. doi: 10.1111/1751-2980.12088

31. McPherson S, Anstee QM, Henderson E, Day CP, Burt AD. Are simple noninvasive scoring systems for fibrosis reliable in patients with NAFLD and normal ALT levels? *Eur J Gastroenterol Hepatol* (2013) 25:652–658. doi: 10.1097/MEG.0b013e32835d72cf

32. Pérez-Gutiérrez OZ, Hernández-Rocha C, Candia-Balboa RA, Arrese MA, Benítez C, Brizuela-Alcántara DC, Méndez-Sánchez N, Uribe M, Chávez-Tapia NC. Validation study of systems for noninvasive diagnosis of fibrosis in nonalcoholic fatty liver disease in Latin population. *Ann Hepatol* (2013) 12:416–424. doi: 10.1016/s1665-2681(19)31004-x

33. Kumar R, Rastogi A, Sharma MK, Bhatia V, Tyagi P, Sharma P, Garg H, Chandan Kumar KN, Bihari C, Sarin SK. Liver stiffness measurements in patients with different stages of nonalcoholic fatty liver disease: Diagnostic performance and clinicopathological correlation. *Dig Dis Sci* (2013) 58:265–274. doi: 10.1007/s10620-012-2306-1

34. Aykut UE, Akyuz U, Yesil A, Eren F, Gerin F, Ergelen R, Celikel CA, Yilmaz Y. A comparison of FibroMeter^TM^ NAFLD Score, NAFLD fibrosis score, and transient elastography as noninvasive diagnostic tools for hepatic fibrosis in patients with biopsy-proven non-alcoholic fatty liver disease. *Scand J Gastroenterol* (2014) 49:1343–1348. doi: 10.3109/00365521.2014.958099

35. Drolz A, Wehmeyer M, Diedrich T, zur Wiesch JS, Lohse AW, Kluwe J. Validation of non-invasive fibrosis assessments in biopsy-proven non-alcoholic fatty liver disease. *J Hepatol* (2017) 66:S150–S151. doi: 10.1016/s0168-8278(17)30573-1

36. Dvorak K, Stritesky J, Petrtyl J, Vitek L, Sroubkova R, Lenicek M, Smid V, Haluzik M, Bruha R. Use of Non-Invasive Parameters of Non-Alcoholic Steatohepatitis and Liver Fibrosis in Daily Practice - An Exploratory Case-Control Study. *PLoS One* (2014) 9:e111551. doi: 10.1371/journal.pone.0111551

37. Ahmed Z, Ren J, Martin D, Walayat S, Moole H, Yong S, Dhillon S. Sa1597 The Development and Validation of a Novel Serological Index to Predict Cirrhosis. *Gastroenterology* (2016) 150:S338. doi: 10.1016/s0016-5085(16)31187-8

38. Simo KA, McKillop IH, McMillan MT, Ahrens WA, Walters AL, Thompson KJ, Kuwada TS, Martinie JB, Iannitti DA, Gersin KS, et al. Does a calculated “nAFLD fibrosis score” reliably negate the need for liver biopsy in patients undergoing bariatric surgery? *Obes Surg* (2014) 24:15–21. doi: 10.1007/s11695-013-1044-6

39. Aida Y, Abe H, Tomita Y, Nagano T, Seki N, Sugita T, Itagaki M, Ishiguro H, Sutoh S, Aizawa Y. Serum Immunoreactive Collagen IV detected by Monoclonal Antibodies as a Marker of Severe Fibrosis in Patients with Non- Alcoholic Fatty Liver Disease. *J Gastrointest Liver Dis* (2015) 24:61–68. doi: 10.15403/jgld.2014.1121.yad

40. Cengiz M, Ozenirler S. Comparative diagnostic accuracy of red cell distribution width-to-platelet ratio versus noninvasive fibrosis scores for the diagnosis of liver fibrosis in biopsy-proven nonalcoholic fatty liver disease. *Eur J Gastroenterol Hepatol* (2015) 27:1293–1299. doi: 10.1097/MEG.0000000000000445

41. Cui J, Ang B, Haufe W, Hernandez C, Verna EC, Sirlin CB, Loomba R. Comparative diagnostic accuracy of magnetic resonance elastography vs. eight clinical prediction rules for non-invasive diagnosis of advanced fibrosis in biopsy-proven non-alcoholic fatty liver disease: a prospective study. *Aliment Pharmacol Ther* (2015) 41:1271–1280. https://doi.org/10.1111/apt.13196

42. Dincses E, Yilmaz Y. Diagnostic usefulness of FibroMeter VCTE for hepatic fibrosis in patients with nonalcoholic fatty liver disease. *Eur J Gastroenterol Hepatol* (2015) 27:1149–1153. doi: 10.1097/MEG.0000000000000409

43. Kawamura Y, Ikeda K, Arase Y, Sorin Y, Fukushima T, Kunimoto H, Hosaka T, Kobayashi M, Saitoh S, Sezaki H, et al. New discriminant score to predict the fibrotic stage of non-alcoholic steatohepatitis in Japan. *Hepatol Int* (2015) 9:269–277. doi: 10.1007/s12072-014-9605-x

44. Pathik P, Ravindra S, Ajay C, Prasad B, Jatin P, Prabha S. Fibroscan versus simple noninvasive screening tools in predicting fibrosis in high-risk nonalcoholic fatty liver disease patients from western India. *Ann Gastroenterol* (2015) 28:281–286. https://doi.org/

45. Petta S, Vanni E, Bugianesi E, Di Marco V, Cammà C, Cabibi D, Mezzabotta L, Craxì A. The combination of liver stiffness measurement and NAFLD fibrosis score improves the noninvasive diagnostic accuracy for severe liver fibrosis in patients with nonalcoholic fatty liver disease. *Liver Int* (2015) 35:1566–1573. doi: 10.1111/liv.12584

46. Shukla A, Kapileswar S, Gogtay N, Joshi A, Dhore P, Shah C, Abraham P, Bhatia S. Simple biochemical parameters and a novel score correlate with absence of fibrosis in patients with nonalcoholic fatty liver disease. *Indian J Gastroenterol* (2015) 34:281–285. doi: 10.1007/s12664-015-0580-5

47. Sjöwall C, Martinsson K, Cardell K, Ekstedt M, Kechagias S. Soluble urokinase plasminogen activator receptor levels are associated with severity of fibrosis in nonalcoholic fatty liver disease. *Transl Res* (2015) 165:658–666. doi: 10.1016/j.trsl.2014.09.007

48. Angulo P, Hui JM, Marchesini G, Bugianesi E, George J, Farrell GC, Enders F, Saksena S, Burt AD, Bida JP, et al. The NAFLD fibrosis score: A noninvasive system that identifies liver fibrosis in patients with NAFLD. *Hepatology* (2007) 45:846–854. doi: 10.1002/hep.21496

49. Subasi CF, Aykut UE, Yilmaz Y. Comparison of noninvasive scores for the detection of advanced fibrosis in patients with nonalcoholic fatty liver disease. *Eur J Gastroenterol Hepatol* (2015) 27:137–141. doi: 10.1097/MEG.0000000000000255

50. Isgro M, Miele L, Cefalo C, Giannace A, Morlacchi C, Rapaccini G, Gasbarrini A, Grieco A, Zuppi C, De Michele T. ELF test as a new non invasive diagnostic tool staging liver fibrosis: validation in a cohort of patients with nonalcoholic fatty liver diasease. *Clin Chem Lab Med* (2014) 52:S1 – S1760. doi: 10.1515/cclm-2014-4041

51. Boursier J, Vergniol J, Guillet A, Hiriart J-B, Lannes A, Le Bail B, Michalak S, Chermak F, Bertrais S, Foucher J, et al. Diagnostic accuracy and prognostic significance of blood fibrosis tests and liver stiffness measurement by FibroScan in non-alcoholic fatty liver disease. *J Hepatol* (2016) 65:570–578. doi: 10.1016/j.jhep.2016.04.023

52. de Cleva R, Duarte LF, Crenitte MRF, de Oliveira CPM, Pajecki D, Santo MA. Use of noninvasive markers to predict advanced fibrosis/cirrhosis in severe obesity. *Surg Obes Relat Dis* (2016) 12:862–867. doi: 10.1016/j.soard.2015.11.011

53. Rath MM, Panigrahi MK, Pattnaik K, Bhuyan P, Kar SK, Misra B, Misra D, Meher C, Agrawal O, Rath J, et al. Histological Evaluation of Non-alcoholic Fatty Liver Disease and Its Correlation with Different Noninvasive Scoring Systems with Special Reference to Fibrosis: A Single Center Experience. *J Clin Exp Hepatol* (2016) 6:291–296. doi: 10.1016/j.jceh.2016.08.006

54. Shoji H, Yoshio S, Mano Y, Kumagai E, Sugiyama M, Korenaga M, Arai T, Itokawa N, Atsukawa M, Aikata H, et al. Interleukin-34 as a fibroblast-derived marker of liver fibrosis in patients with non-alcoholic fatty liver disease. *Sci Rep* (2016) 6:1–11. doi: 10.1038/srep28814

55. Siddiqui MS, Patidar KR, Boyett S, Luketic VA, Puri P, Sanyal AJ. Performance of non-invasive models of fibrosis in predicting mild to moderate fibrosis in patients with non-alcoholic fatty liver disease. *Liver Int* (2016) 36:572–579. doi: 10.1111/liv.13054

56. Treeprasertsuk S, Piyachaturawat P, Soontornmanokul T, Wisedopas-Klaikaew N, Komolmit P, Tangkijavanich P. Accuracy of noninvasive scoring systems to assess advanced liver fibrosis in Thai patients with nonalcoholic fatty liver disease. *Asian Biomed* (2016) 10:S49–S55. doi: 10.5372/1905-7415.1000.521

57. Miller A, Li N, Hinton A, Mumtaz K. Performance of Lab-Based Scoring Tests for the Assessment ofHepatic Fibrosis Compared to the Liver Biopsy Among Patients With Non-Alcoholic Fatty Liver Disease. *Am J Gastroenterol* (2019) 38:1–46.

58. Kobayashi N, Kumada T, Toyoda H, Tada T, Ito T, Kage M, Okanoue T, Kudo M. Ability of Cytokeratin-18 Fragments and FIB-4 Index to Diagnose Overall and Mild Fibrosis Nonalcoholic Steatohepatitis in Japanese Nonalcoholic Fatty Liver Disease Patients. *Dig Dis* (2017) 35:521–530. doi: 10.1159/000480142

59. Guha IN, Parkes J, Roderick P, Chattopadhyay D, Cross R, Harris S, Kaye P, Burt AD, Ryder SD, Aithal GP, et al. Noninvasive markers of fibrosis in nonalcoholic fatty liver disease: Validating the European Liver Fibrosis Panel and exploring simple markers. *Hepatology* (2008) 47:455–460. doi: 10.1002/hep.21984

60. Loong TCW, Wei JL, Leung JCF, Wong GLH, Shu SST, Chim AML, Chan AWH, Choi PCL, Tse YK, Chan HLY, et al. Application of the combined FibroMeter vibration-controlled transient elastography algorithm in Chinese patients with non-alcoholic fatty liver disease. *J Gastroenterol Hepatol* (2017) 32:1363–1369. doi: 10.1111/jgh.13671

61. McPherson S, Hardy T, Dufour JF, Petta S, Romero-Gomez M, Allison M, Oliveira CP, Francque S, Van Gaal L, Schattenberg JM, et al. Age as a Confounding Factor for the Accurate Non-Invasive Diagnosis of Advanced NAFLD Fibrosis. *Am J Gastroenterol* (2017) 112:740–751. doi: 10.1038/ajg.2016.453

62. Miele L, De Michele T, Marrone G, Antonietta Isgrò M, Basile U, Cefalo C, Biolato M, Maria Vecchio F, Lodovico Rapaccini G, Gasbarrini A, et al. Enhanced liver fibrosis test as a reliable tool for assessing fibrosis in nonalcoholic fatty liver disease in a clinical setting. *Int J Biol Markers* (2017) 32:e397–e402. doi: 10.5301/ijbm.5000292

63. Nassif AT, Nagano TA, Okayama S, Nassif LS, Branco Filho A, Sampaio Neto J. Performance of the Bard Scoring System in Bariatric Surgery Patients with Nonalcoholic Fatty Liver Disease. *Obes Surg* (2017) 27:394–398. doi: 10.1007/s11695-016-2284-z

64. Okajima A, Sumida Y, Taketani H, Hara T, Seko Y, Ishiba H, Nishimura T, Umemura A, Nishikawa T, Yamaguchi K, et al. Liver stiffness measurement to platelet ratio index predicts the stage of liver fibrosis in non-alcoholic fatty liver disease. *Hepatol Res* (2017) 47:721–730. doi: 10.1111/hepr.12793

65. Peleg N, Issachar A, Sneh-Arbib O, Shlomai A. AST to Platelet Ratio Index and fibrosis 4 calculator scores for non-invasive assessment of hepatic fibrosis in patients with non-alcoholic fatty liver disease. *Dig Liver Dis* (2017) 49:1133–1138. doi: 10.1016/j.dld.2017.05.002

66. Itoh Y, Seko Y, Shima T, Nakajima T, Mizuno K, Kawamura Y, Akuta N, Ito K, Kawanaka M, Hiramatsu A, et al. Accuracy of non-invasive scoring systems for diagnosing non-alcoholic steatohepatitis-related fibrosis: Multicenter validation study. *Hepatol Res* (2018) 48:1099–1107. doi: 10.1111/hepr.13226

67. Labenz C, Huber Y, Kalliga E, Nagel M, Ruckes C, Straub BK, Galle PR, Wörns MA, Anstee QM, Schuppan D, et al. Predictors of advanced fibrosis in non-cirrhotic non-alcoholic fatty liver disease in Germany. *Aliment Pharmacol Ther* (2018) 48:1109–1116. doi: 10.1111/apt.14976

68. Seth A, Balakrishnan M, Thrift A, Sood GK. Utility of four non-invasive scores in predicting advanced fibrosis in a predominantly hispanic population. *Am J Gastroenterol* (2016) 111:S374. http://www.embase.com/search/results?subaction=viewrecord&from=export&id=L612870197%0Ahttp://dx.doi.org/10.1038/ajg.2016.359

69. Takeuchi H, Sugimoto K, Oshiro H, Iwatsuka K, Kono S, Yoshimasu Y, Kasai Y, Furuichi Y, Sakamaki K, Itoi T. Liver fibrosis: noninvasive assessment using supersonic shear imaging and FIB4 index in patients with non-alcoholic fatty liver disease. *J Med Ultrason* (2018) 45:243–249. doi: 10.1007/s10396-017-0840-3

70. Harrison SA, Oliver D, Arnold HL, Gogia S, Neuschwander-Tetri BA. Development and validation of a simple NAFLD clinical scoring system for identifying patients without advanced disease. *Gut* (2008) 57:1441–1447. doi: 10.1136/gut.2007.146019

71. Anstee QM, Lawitz EJ, Alkhouri N, Wong VW, Romero‐Gomez M, Okanoue T, Trauner M, Kersey K, Li G, Han L, et al. Noninvasive Tests Accurately Identify Advanced Fibrosis due to NASH: Baseline Data From the STELLAR Trials. *Hepatology* (2019) 70:1521–1530. doi: 10.1002/hep.30842

72. Kolhe KM, Amarapurkar A, Parikh P, Chaubal A, Chauhan S, Khairnar H, Walke S, Ingle M, Pandey V, Shukla A. Aspartate transaminase to platelet ratio index (APRI) but not FIB-5 or FIB-4 is accurate in ruling out significant fibrosis in patients with non-alcoholic fatty liver disease (NAFLD) in an urban slum-dwelling population. *BMJ Open Gastroenterol* (2019) 6:1–6. doi: 10.1136/bmjgast-2019-000288

73. Polyzos SA, Slavakis A, Koumerkeridis G, Katsinelos P, Kountouras J. Noninvasive Liver Fibrosis Tests in Patients with Nonalcoholic Fatty Liver Disease: An External Validation Cohort. *Horm Metab Res* (2019) 51:134–140. doi: 10.1055/a-0713-1330

74. Shima T, Sakai K, Oya H, Katayama T, Mitsumoto Y, Mizuno M, Kanbara Y, Okanoue T. Diagnostic accuracy of combined biomarker measurements and vibration-controlled transient elastography (VCTE) for predicting fibrosis stage of non-alcoholic fatty liver disease. *J Gastroenterol* (2019) 55:100–112. doi: 10.1007/s00535-019-01626-1

75. Siddiqui MS, Yamada G, Vuppalanchi R, Van Natta M, Loomba R, Guy C, Brandman D, Tonascia J, Chalasani N, Neuschwander-Tetri B, et al. Diagnostic Accuracy of Noninvasive Fibrosis Models to Detect Change in Fibrosis Stage. *Clin Gastroenterol Hepatol* (2019) 17:1877-1885.e5. doi: 10.1016/j.cgh.2018.12.031

76. Staufer K, Halilbasic E, Spindelboeck W, Eilenberg M, Prager G, Stadlbauer V, Posch A, Munda P, Marculescu R, Obermayer-Pietsch B, et al. Evaluation and comparison of six noninvasive tests for prediction of significant or advanced fibrosis in nonalcoholic fatty liver disease. *United Eur Gastroenterol J* (2019) 7:1113–1123. doi: 10.1177/2050640619865133

77. Tomeno W, Imajo K, Kuwada Y, Ogawa Y, Kikuchi M, Honda Y, Kato T, Kessoku T, Kirikoshi H, Yoneda M, et al. Distribution of liver stiffness in non-alcoholic fatty liver disease with higher fibrosis-4 index than low cut-off index. *J Gastroenterol Hepatol* (2019) 34:1411–1416. doi: 10.1111/jgh.14559

78. Yang M, Jiang L, Wang Y, Li X, Zhou G, Zou Z, Han T, Nan Y, Lu F, Zhao J. Step layered combination of noninvasive fibrosis models improves diagnostic accuracy of advanced fibrosis in nonalcoholic fatty liver disease. *J Gastrointest Liver Dis* (2019) 28:289–296. doi: 10.15403/jgld-420

79. Bril F, McPhaul MJ, Caulfield MP, Castille J-M, Poynard T, Soldevila-Pico C, Clark VC, Firpi-Morell RJ, Lai J, Cusi K. Performance of the SteatoTest, ActiTest, NashTest and FibroTest in a multiethnic cohort of patients with type 2 diabetes mellitus. *J Investig Med* (2019) 67:303–311. doi: 10.1136/jim-2018-000864

80. Broussier T, Lannes A, Zuberbuhler F, Oberti F, Fouchard I, Hunault G, Cales P, Boursier J. Simple blood fibrosis tests reduce unnecessary referrals for specialized evaluations of liver fibrosis in NAFLD and ALD patients. *Clin Res Hepatol Gastroenterol* (2020) 44:349–355. doi: 10.1016/j.clinre.2019.07.010

81. Loaeza-del-Castillo A, Paz-Pineda F, Oviedo-Cárdenas E, Sánchez-Ávila F, Vargas-Vorácková F. AST to platelet ratio index (APRI) for the noninvasive evaluation of liver fibrosis. *Ann Hepatol* (2008) 7:350–357. doi: 10.1016/s1665-2681(19)31836-8

82. Meneses D, Olveira A, Corripio R, del Carmen Méndez M, Romero M, Calvo-Viñuelas I, Herranz L, Vicent D, De-Cos-Blanco AI. Performance of Noninvasive Liver Fibrosis Scores in the Morbid Obese Patient, Same Scores but Different Thresholds. *Obes Surg* (2020) 30:2538–2546. doi: 10.1007/s11695-020-04509-0

83. Lang S, Farowski F, Martin A, Wisplinghoff H, Vehreschild MJGT, Krawczyk M, Nowag A, Kretzschmar A, Scholz C, Kasper P, et al. Prediction of advanced fibrosis in non-alcoholic fatty liver disease using gut microbiota-based approaches compared with simple non-invasive tools. *Sci Rep* (2020) 10:1–9. doi: 10.1038/s41598-020-66241-0

84. Liu WY, Zheng KI, Pan XY, Ma HL, Zhu PW, Wu XX, Rios RS, Targher G, Byrne CD, Wang XD, et al. Effect of PNPLA3 polymorphism on diagnostic performance of various noninvasive markers for diagnosing and staging nonalcoholic fatty liver disease. *J Gastroenterol Hepatol* (2020) 35:1057–1064. doi: 10.1111/jgh.14894

85. Singh A, Gosai F, Siddiqui MT, Gupta M, Lopez R, Lawitz E, Poordad F, Carey W, McCullough A, Alkhouri N. Accuracy of noninvasive fibrosis scores to detect advanced fibrosis in patients with type-2 diabetes with biopsy-proven nonalcoholic fatty liver disease. *J Clin Gastroenterol* (2020) 54:891–897. doi: 10.1097/MCG.0000000000001339

86. Qureshi K, Clements RH, Abrams GA. The utility of the “NAFLD fibrosis score” in morbidly obese subjects with NAFLD. *Obes Surg* (2008) 18:264–270. doi: 10.1007/s11695-007-9295-8

87. Hagström H, Nasr P, Ekstedt M, Stål P, Hultcrantz R, Kechagias S. Accuracy of Noninvasive Scoring Systems in Assessing Risk of Death and Liver-Related Endpoints in Patients With Nonalcoholic Fatty Liver Disease. *Clin Gastroenterol Hepatol* (2019) 17:1148-1156.e4. doi: 10.1016/j.cgh.2018.11.030

88. Angelidi A, Angelidi M, Papazafiropoulou A, Anagnostopoulou K, Vagena E, Velissaris V, Markakis V, Kamaratos A, Melidonis ; Evaluation of different scores to predict nonalcoholic fatty liver disease in overweight or obese patients with type 2 diabetes. *Obes Facts* (2017) 10:1–259. doi: 10.1159/000468958

89. Boursier J, Vergniol J, Lannes A, Hiriart J-B, Oberti F, Le Bail B, Michalak S, Chermak F, Bertrais S, Foucher J, et al. The combination of Fibroscan with blood markers in the fibrometerVCTE significantly reduces the use of liver biopsy for the assessment of advanced fibrosis in non-alcoholic fatty liver disease. *J Hepatol* (2017) 66:S161–S162. doi: 10.1016/S0168-8278(17)30597-4

90. Boursier J, Guillaume M, Leroy V, Irlès M, Roux M, Lannes A, Foucher J, Zuberbuhler F, Delabaudière C, Barthelon J, et al. New sequential combinations of non-invasive fibrosis tests provide an accurate diagnosis of advanced fibrosis in NAFLD. *J Hepatol* (2019) 71:389–396. doi: 10.1016/j.jhep.2019.04.020

91. Gallego-Durán R, Pareja MJ, Ranchal I, Ampuero J, Camacho IM, Chaves P, Fenandez G, López RA, García-Monzón C, Andrade RJ, et al. Validation of FibroMax and NAFLDscore in a cohort of NAFLD Spanish patients. *Hepatology* (2012) 56:191–1144.

92. Joo SK, Kim JH, Oh S, Kim BG, Lee KL, Kim HY, Jung YJ, Woo HS, Moon MH, Chang MS, et al. Prospective comparison of noninvasive fibrosis assessment to predict advanced fibrosis or cirrhosis in Asian patients with hepatitis C. *J Clin Gastroenterol* (2015) 49:697–704. doi: 10.1097/MCG.0000000000000215

93. Jouness RIK, Rosso C, Petta S, Cucco M, Marietti M, Caviglia GP, Abate ML, Cammà C, Smedile A, Craxì A, et al. The combination of Index of NASH score and liver stiff- ness improves the noninvasive diagnostic accuracy for severe liver fibrosis in patients with Non-Alcoholic Fatty Liver Disease. *Hepatology* (2016) 64:

94. Lambrecht J, Verhulst S, Reynaert H, van Grunsven LA. The miRFIB-Score: A Serological miRNA-Based Scoring Algorithm for the Diagnosis of Significant Liver Fibrosis. *Cells* (2019) 8:

95. Luger M, Kruschitz R, Kienbacher C, Traussnigg S, Langer FB, Schindler K, Würger T, Wrba F, Trauner M, Prager G, et al. Prevalence of Liver Fibrosis and its Association with Non-invasive Fibrosis and Metabolic Markers in Morbidly Obese Patients with Vitamin D Deficiency. *Obes Surg* (2016) 26:2425–2432. doi: 10.1007/s11695-016-2123-2

96. Miele L, De Michele T, Isgrò M, Marrone G, Cefalo C, Biolato M, Rapaccini G, Gasgarrini A, Zuppi C, Grieco A. ELF test is a reliable non invasive test for fibrosis in NAFLD subjects. *J Hepatol* (2015) 62:S736.

97. Munteanu M, Tiniakos D, Anstee Q, Charlotte F, Marchesini G, Bugianesi E, Trauner M, Romero Gomez M, Oliveira C, Day C, et al. Diagnostic performance of FibroTest, SteatoTest and ActiTest in patients with NAFLD using the SAF score as histological reference. *Aliment Pharmacol Ther* (2016) 44:877–889. doi: 10.1111/apt.13770

98. Nascimbeni F, Petta S, Romero-Gomez M, Marchesini G, Buagianesi E, Bellantani S, Day CP, Bedossa P, Oliveira CP, Trauner, et al. Substantial variability of the diagnostic accuracy of serum fibrosis markers across multiple cohorts of nash patients in various centers : the case for the negative predictive value. *Hepatology* (2015) 2:1252–1305.

99. Pastor-Ramírez H, Aller R, Gallego-Durán R, Bañales J, Arias-Lotes M, García-Monzón C, Ampuero J, Vilar-Gómez E, Escudero A, Caballería J, et al. Validation of Non-Invasive Methods for Advanced Fibrosis Detection in NAFLD Patients. *Inflamm Intest Dis* (2017) 2:1–92. doi: 10.1159/000478719

100. Saez E, Conde I, Blazquez T, Garcia-Morales N, Perez J, Tenias JM, Saiz V, Zaragoza A. Performance of the fibroscan and other noninvasive scales for detecting hepatic fibrosis in patients with nonalcoholic fatty liver disease. *J Hepatol* (2017) 66:S156. doi: 10.1016/s0168-8278(17)30585-8

101. Stauber RE, Staufer K, Stift J, Marculescu R, Obermayer-Pietsch B, Trauner M, Lackner K. Enhanced liver fibrosis (ELF) score accurately detects advanced fibrosis in nonalcoholic fatty liver disease (NAFLD). *J Hepatol* (2018) 68:S563. doi: 10.1016/s0168-8278(18)31383-7

102. Tanwar S, Trembilng P, Thorbun D, Guha I, Parkes J, Kaye P, Burt A, Ryder S, Aithal G, Day C, et al. Direct serum markers are more accurate than simple marker panels for the detection of fibrosis in non-alcoholic fatty liver disease (NAFLG). *Gut* (2012) 61:A414.2-A415. doi: 10.1136/gutjnl-2012-302514d.287

103. Huang CYW, Seah JJ, Kam JW, Ang TL, Fock KM, Teo EK, Tan J, Ang DSW, Ong JPL, Kwek A, et al. For prediction of Non-alcoholic Fatty Liver Disease (NAFLD) induced Liver Cirrhosis, modified AST to Platelet Ratio Index (m- APRI) performs better than other non-invasive scores. *Hepatol Int* (2019) 13:S1–S266.

104. Joo SK, Kim W, Jung YJ. Obesity and steatosis severity affect diagnostic performances of noninvasive fibrosis tests in nonalcoholic fatty liver disease. *J Hepatol* (2017) 66:S590–S591. doi: 10.1016/s0168-8278(17)31607-0

105. Kao WY, Chang IW, Chen CL, Su CW, Fang SU, Tang JH, Chang CC, Chang YJ, Wang W. Fibroscan-Based Score to Predict Significant Liver Fibrosis in Morbidly Obese Patients with Nonalcoholic Fatty Liver Disease. *Obes Surg* (2020) 30:1249–1257. doi: 10.1007/s11695-019-04192-w

106. Kim D, Yu S, Chung G, Lee J, Kim W, Kim Y, Yoon J-H, Lee H-S. Comparison of Non-Invasive Markers of Fibrosis in the Asian Non-Alcoholic Fatty Liver Disease’S Population With Low Prevalence of Advanced Fibrosis. *J Hepatol* (2011) 54:S209–S361. doi: 10.1016/s0168-8278(11)60850-7

107. Prasad SS, Ranjan KC, Kanta SS, Dinesh M, Gautam N, Resu K, Preetam N, Debakanta M, Subhendu P, Kumar BR, et al. Utility of noninvasive scoring systems of fibrosis for detecting advanced fibrosis in patients with leaner nonalcoholic fatty liver disease (NAFLD). *Hepatol Int* (2020) 14:S1–S470.

108. Uy D, Cua I, Bocobo J, Cervantes J, Edano J. FIB-4: more accurate non-invasive assessment for advanced fi brosis among patients with NAFLD. *J Gastroenterol Hepatol* (2011) 26:16–288.

109. Yoneda M, Imajo K, Eguchi Y, Fujii H, Sumida Y, Hyogo H, Ono M, Suzuki Y, Kawaguchi T, Aoki N, et al. Noninvasive scoring systems in patients with nonalcoholic fatty liver disease with normal alanine aminotransferase levels. *J Gastroenterol* (2013) 48:1051–1060. doi: 10.1007/s00535-012-0704-y

110. Zhou YJ, Ye FZ, Li YY, Pan XY, Chen YX, Wu XX, Xiong JJ, Liu WY, Xu SH, Chen YP, et al. Individualized risk prediction of significant fibrosis in non-alcoholic fatty liver disease using a novel nomogram. *United Eur Gastroenterol J* (2019) 7:1124–1134. doi: 10.1177/2050640619868352

111. Alkhouri N, Allende D, Guirguis J, Shaker M, Yeriaj L, Lopez R, Youssef D, Nobili V. Commonly Used Hepatic Fibrosis Scores Have Poor Performance in Young Adult With Nonalcoholic Fatty Liver Disease. *Am J Gastroenterol* (2015) 110:S847. doi: 10.1038/ajg.2015.277

112. Adams LA, George J, Rossi E, van der Poorten D, Kench J, DeBoer B, Macquillan GC, Jeffrey GP. Non-invasive prediction of liver fibrosis in nonalcoholic fatty liver disease. *Hepatology* (2008) 48:506–608.

113. Arora S, Young S, Singal A. Comparison of Fibrosis Scoring Tools in predicting Liver Fibrosis in Nonalcoholic Fatty Liver Disease. *Hepatology* (2016) 64:

114. Eddowes P, Allison M, Tsochatzis E, Anstee Q, Sheridan D, Guha IN, Cobbold J, Paradis V, Newsome PBP. Combination of FibroScan and FibroMeter (FibroMeter VCTE) improves identification of patients with advanced fibrosis in patients with NAFLD. *J Hepatol* (2019) 70:e766–e767. doi: 10.1016/s0618-8278(19)31525-7

115. Fagan KJ, Pretorius CJ, Horsfall LU, Irvine KM, Wilgen U, Choi K, Fletcher LM, Tate J, Melino M, Nusrat S, et al. ELF score ≥9.8 indicates advanced hepatic fibrosis and is influenced by age, steatosis and histological activity. *Liver Int* (2015) 35:1673–1681. doi: 10.1111/liv.12760

116. Kosick H, Cerocchi O, Sebastiani G, Patel K. Non-invasive prediction of advanced fibrosis in NAFLD—A stepwise, algorithmic approach. *Can Liver J* (2019) 2:

117. Miao CL, Pineda-Bonilla JJ, Smith RE, Zhanna V, Vladimir Proudan Proudan V, Pogrebnaya Z V, Wood C, Still CD. Independent predictors and non-invansive markers of nonalcoholic steatohepatitis and fibrosis in morbidly obese patients. *Hepatology* (2010) 52:627–724.

118. Anam MK, Alam S, Ahmad N. Validation of the BARD (BMI, AST/ALT ratio, DMt2) scoring system for detection of fibrosis in patients with nonalcoholic fatty liver disease. *Hepatol Int* (2017) 11:1–1093. doi: 10.1007/s12072-016-9783-9

119. Guturu P, Steffer K, Petersen JR, Snyder N. A new risk index for the estimation of fibrosis in non alcoholic fatty liver disease (NAFLD): Comparison with the mayo score and the ast platelet ration index (APRI). *Hepatology* (2008) 48:506–608.

120. Kruger F, Daniels C, Kidd M, Swart G, Brundyn K, van Rensburg C, Kotze M. APRI, a non-invasive for advanced fibrosis in NASH, and new proposed algorithm for the detection of advanced fibrosis. *SAMJ* (2008) 98:633–648.

121. Shaheen A, Rye P, Urbanski S, Swain M, Jayakumar S. Sequential Algorithm of Non-Invasive Tools to Assess Severe Fibrosis in Non Alcoholic Fatty Liver Disease. *Can J Gastroenterol Hepatol* (2016)1–204. doi: 10.1155/2016/4792898

122. Thanapirom K, Treeprasertsuk S, Chaopathomkul B, Tanpowpong N, Suksawatamnuay S, Thaimai P, Sittisomwong S, Sonsiri K, Komolmit P. Correlation of Magnetic Resonance Elastography, Fibroscan, Shear Wave Elastography, APRI and FIB-4 for Staging of Liver Fibrosis. *Gastroenterology* (2017) 152:S1107–S1108. doi: 10.1016/s0016-5085(17)33731-9

123. Bril F, McPhaul MJ, Caulfield MP, Clark VC, Soldevilla-Pico C, Firpi-Morell RJ, Lai J, Shiffman D, Rowland CM, Cusi K. Performance of plasma biomarkers and diagnostic panels for nonalcoholic steatohepatitis and advanced fibrosis in patients with type 2 diabetes. *Diabetes Care* (2020) 43:290–297. doi: 10.2337/dc19-1071

124. Brandman D, Boyle M, McPherson S, Van Natta M, Sanyal A, Kowdley K, Tetri B, Chalassani N, Abdelmalek M, Terrault NA, et al. Comparison of clinical prediction rules for detection of cirrhosis in non-alcoholic fatty liver disease: a multicenter, international, collaborative study- NASH CRN (USA) and Newcastle (UK) Cohort. *J Hepatol* (2017) 66:S69–S70. doi: 10.1016/s0168-8278(17)30400-2

125. Kruger FC, Daniels CR, Kidd M, Swart G, Brundyn K, van Rensburg C, Kotze M. APRI: A simple bedside marker for advanced fibrosis that can avoid liver biopsy in patients with NAFLD/NASH. *South African Med J* (2011) 101:477–480.

126. Singh T, Frakes CM, Lopez R, McCullough A. Comparison of Prediction Models of Advanced Fibrosis in Morbidly and Non-Morbidly Obese Patients with Nonalcoholic Fatty Liver Disease. *Gastroenterology* (2018) 154:S-1359. doi: 10.1016/s0016-5085(18)34445-7

127. Angulo P, George J, Day CP, Vanni E, Russell L, De la Cruz AC, Liaquat H, Mezzabotta L, Lee E, Bugianesi E. Serum Ferritin Levels Lack Diagnostic Accuracy for Liver Fibrosis in Patients With Nonalcoholic Fatty Liver Disease. *Clin Gastroenterol Hepatol* (2014) 12:1163-1169.e1. doi: 10.1016/j.cgh.2013.11.035

128. Le P, Yu P-C, Singh A, Nguyen C, Singh T, McCullough A, Rothberg M. Validation of Noninvasive Fibrosis Scores in Prediabetic Patients with Nonalcoholic Fatty Liver Disease. *Gastroenterology* (2018) 154:S-1169-S-1170. doi: 10.1016/s0016-5085(18)33874-5

129. Marella HK, Reddy YK, Jiang Y, Ganguli S, Podila PSB, Snell PD, Kovalic AJ, Cholankeril G, Singal AK, Nair S, et al. Accuracy of noninvasive fibrosis scoring systems in african american and white patients with nonalcoholic fatty liver disease. *Clin Transl Gastroenterol* (2020) 11:1–12. doi: 10.14309/ctg.0000000000000165

130. Guillaume M, Moal V, Delabaudiere C, Zuberbuhler F, Robic MA, Lannes A, Metivier S, Oberti F, Gourdy P, Fouchard-Hubert I, et al. Direct comparison of the specialised blood fibrosis tests FibroMeterV2G and Enhanced Liver Fibrosis score in patients with non-alcoholic fatty liver disease from tertiary care centres. *Aliment Pharmacol Ther* (2019) 50:1214–1222. doi: 10.1111/apt.15529

131. Abe M, Miyake T, Kuno A, Imai Y, Sawai Y, Hino K, Hara Y, Hige S, Sakamoto M, Yamada G, et al. Association between Wisteria floribunda agglutinin-positive Mac-2 binding protein and the fibrosis stage of non-alcoholic fatty liver disease. *J Gastroenterol* (2014) 50:776–784. https://doi.org/10.1007/s00535-014-1007-2

132. Chan W-K, Nik Mustapha NR, Mahadeva S. A novel 2-step approach combining the NAFLD fibrosis score and liver stiffness measurement for predicting advanced fibrosis. *Hepatol Int* (2014) 9:594–602. doi: 10.1007/s12072-014-9596-7

133. Zou C, Wang Q, Ou X, Zhao X, Wang M, Wang P, Wu S, Kong Y, Jia J. A noninvasive diagnostic system for cirrhosis in patients with non-alcoholic fatty liver disease. *Hepatol Int* (2019) 13:S1–S266.

134. de Carli M AL, de Carli LA, Correa MB, Junqueira G, Tovo CV, Coral GP. Performance of noninvasive scores for the diagnosis of advanced liver fibrosis in morbidly obese with nonalcoholic fatty liver disease. *Eur J Gastroenterol Hepatol* (2020) 32:420–425. doi: 10.1097/MEG.0000000000001519

135. Fujii H, Enomoto M, Fukushima W, Tamori A, Sakaguchi H, Kawada N. Applicability of BARD score to Japanese patients with NAFLD. *Gut* (2009) 58:1566–7. doi: 10.1136/gut.2009

136. Inadomi C, Takahashi H, Ogawa Y, Oeda S, Imajo K, Kubotsu Y, Tanaka K, Kessoku T, Okada M, Isoda H, et al. Accuracy of the Enhanced Liver Fibrosis test, and combination of the Enhanced Liver Fibrosis and non-invasive tests for the diagnosis of advanced liver fibrosis in patients with non-alcoholic fatty liver disease. *Hepatol Res* (2020) 50:682–692. doi: 10.1111/hepr.13495

137. Kim D, Kim WR, Talwalkar JA, Kim HJ, Ehman RL. Advanced Fibrosis in Nonalcoholic Fatty Liver Disease: Noninvasive Assessment with MR Elastography. *Radiology* (2013) 268:411–419. doi: 10.1148/radiol.13121193

138. Petta S, Wong VWS, Cammà C, Hiriart JB, Wong GLH, Vergniol J, Chan AWH, Di Marco V, Merrouche W, Chan HLY, et al. Serial combination of non-invasive tools improves the diagnostic accuracy of severe liver fibrosis in patients with NAFLD. *Aliment Pharmacol Ther* (2017) 46:617–627. doi: 10.1111/apt.14219

139. Lardi L, Lul R, Port G, Coral G, Peres A, Dornelles G, Branco F, Fernandes S, Leães C, Mattos A, et al. Fibromax and inflamatory markers cannot replace liver biopsy in the evaluation of non-alcoholic fatty liver disease. *Minerva Gastroenterol* (2020) 68:85–90. doi: 10.23736/s2724-5985.20.02746-4
